# Supplementary material for: Two-edge-resolved three-dimensional non-line-of-sight imaging with an ordinary camera
Source: Nat Commun. 2024 Feb 7;15:1162. doi: 10.1038/s41467-024-45397-7 (PMC11258226; doi:10.1038/s41467-024-45397-7)
Supplement: Supplementary file 1 — Supplementary Information [file 41467_2024_45397_MOESM1_ESM.pdf]

Supplementary Information for

Two-edge-resolved three-dimensional non-line-of-sight imaging with an ordinary camera

Robinson Czajkowski & John Murray-Bruce

Department of Computer Science and Engineering,
University of South Florida,
4202 E. Fowler Avenue,
Tampa, Florida 33620, USA

This supplemental document contains the following supplementary notes:

1 Acquisition Configuration and Light Transport Model **3**
1.1 Acquisition Configuration 3
1.2 Projected-Elevation Spherical Coordinates 3
1.3 Hidden Scene Representation Model 5
1.4 The Projected-Elevation Spherical Coordinates Light Transport Model 6
1.5 Computational Field of View 8

2 Fisher Information and Cramér-Rao Bound Analyses **11**
2.1 Special case I: No-edge configuration 12
2.2 Special case II: One-edge configuration 13
2.3 One-edge vs Two-edge Configurations: A Crámer-Rao Bound Comparison 13
2.4 Comparing Per Cluster Range vs Per Elemental Surface Range Estimation 15

3 Fisher Information Orthogonality of the Projected-Elevation Spherical Coordinate System **20**
3.1 Proof of Fisher information orthogonality for an arbitrary hidden scene point 20
3.2 Example I: Fisher information matrices for a hidden scene point 22
3.3 Example II: CRB matrices for a hidden scene point 23

4 Inversion method: Proposed TERI reconstruction algorithm **24**
4.1 Accelerated project gradient algorithm for range reconstruction 25
4.2 Variant of TERI algorithm I: No total variation refinement 25

5 Additional Reconstructions **28**
5.1 Reconstructions from Single Snapshots 28
5.2 Reconstructions in Visible Side Ambient Light: Experimental details and additional experiment 31
5.3 Single Object at Varying Ranges 35
5.4 Information orthogonality: An experimental demonstration 37
5.5 Scenes that irradiate light from all angles 40

6 Alternative reconstruction algorithms **43**
6.1 Inverting a Non-linear Model for Range without Clustering 43
6.2 Linear Model Inversion: Sequential Reconstruction 44
6.3 Linear Model Inversion: Joint Non-sequential Reconstruction 47

7 Towards principled cluster refinements: Feasibility of splitting and merging clusters **52**

Supplementary Note 1

S1 Acquisition Configuration and Light Transport Model

The proposed passive non-line-of-sight (NLOS) imaging approach consists of a new measurement acquisition configuration, a complementary hidden scene representation, and an efficient 3D reconstruction strategy. This section details the acquisition configuration and highlights some of its important benefits (Section S1.1), details the proposed coordinate system (Section S1.2), along with the corresponding light transport model (Section S1.3) which relates the unknown hidden scene to the intensity measurements in an observation photograph, through the tailored hidden scene representation. The hidden scene representation model results from discretising the hidden scene volume in the proposed projected-elevation spherical coordinates.

The chosen coordinate system also facilitates the computation of a closed-form expression for modelling the potential contribution of each surface element in an observation photograph. Section S1.3 also shows a derivation of the aforementioned closed-form expression before concluding with the discrete forward model required for hidden scene reconstruction.

S1.1 Acquisition Configuration

This NLOS imaging approach exploits two orthogonal edges of a visible occluding structure, such as a doorway's horizontal and vertical edges. Because the horizontal edge of such two-edge occluders is ubiquitous at the top of doorways, penumbra due to the light from the hidden scene being occluded by the doorway head (i.e., the top wall of the doorway containing the horizontal edge) will mostly appear on the ceiling plane. Hence, photographs of the penumbra formed on the ceiling plane are used instead of the floor. The resulting measurement acquisition configuration is shown in Supplementary Figure 1 (together with the conventional corner camera configuration [1]).

Most indoor scenes are typically illuminated by overhead, ceiling, illumination. These overhead light sources will almost always be blocked by the top of the doorway from reaching the observation plane. This has the additional benefit that extremely bright overhead light fixtures will not overwhelm the contributions from the hidden scene objects of interest to the ceiling plane. Supplementary Figure 4 shows the region of the hidden scene that is completely occluded by the door head. This region is formed from all the points that are close to the hidden scene's ceiling plane and as such cannot illuminate the visible scene ceiling due to being occluded by the doorway head. Most overhead illumination for many indoor scenes will likely be overhead lights and lie in this region.

S1.2 Projected-Elevation Spherical Coordinates

A specialized coordinate system, dubbed *projected-elevation spherical coordinates*, for identifying an arbitrary hidden scene point that follows from the geometry of the occluding structure was developed and utilized in this work. This specially-crafted coordinate system provides the basis for an *information orthogonal* [7] 3D representation of arbitrary hidden scenes as described in Section S1.3. In this new coordinate system, an arbitrary 3D location in the hidden scene is identified by: *(i)* its distance ρ from the origin; *(ii)* its azimuthal angle θ relative to the positive x-axis; and, *(iii)* its projected-elevation angle ψ. While ρ and θ are the usual range and azimuthal angle coordinates used in spherical coordinates, the projected-elevation angle ψ differs from the elevation angle of spherical coordinates; instead, it denotes the angle formed by the x-axis and the line from the origin to the projection of the hidden scene point onto the xz-plane (see Supplementary Figure 1(a) and insets). The triple (ρ, θ, ψ) uniquely identifies a point in the hidden 3D volume. In contrast, only 1D or 2D plan view reconstructions of the hidden volume are sought by prior corner camera demonstrations: in 1D demonstrations [1, 3], the coordinate along azimuth θ is used; whereas for 2D plan view reconstructions based on a single doorway edge [4] hidden scene points were parameterized by their distances along the floor from the origin and their azimuthal angles (see Supplementary Figure 1(b) and inset), while in their configuration which exploits two

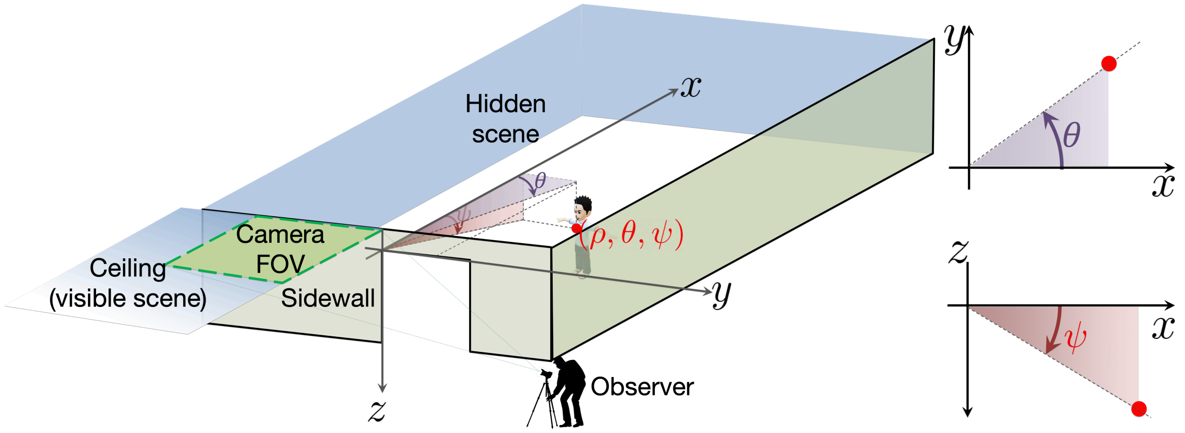

(a) Proposed acquisition configuration (left) and projected-elevation spherical coordinates (plan view: top-right, side view: bottom-right).

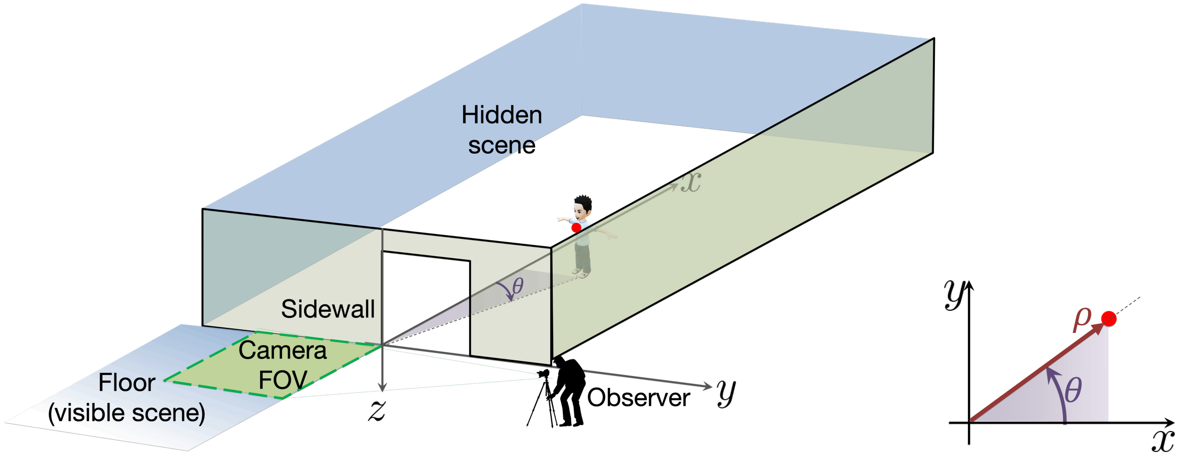

(b) Corner camera acquisition configuration (left) and polar coordinates (right).

Supplementary Figure 1: Acquisition configurations and the project-elevation spherical coordinates. The observation plane is chosen to be the ceiling in the proposed configuration (a) to maximize the utility of both the horizontal and vertical edges of the doorway. In contrast, all previous [1, 2, 3, 4, 5, 6] corner camera-based configurations obtain measurements on the floor (b). The insets in (a) and (b) show the coordinate system utilized by each acquisition configuration for representing an arbitrary point (indicated by the red dot).

vertical edges, Krska *et al* [2] used two azimuthal angles (one for each vertical edge), called biangular coordinates [8], to identify a point.

Transforming from projected-elevation spherical coordinates to Cartesian coordinates is achievable using the following set of equations:

$$x = \frac{\rho}{\sqrt{1 + \tan^2(\theta) + \tan^2(\psi)}}, \tag{1}$$

$$y = \tan(\theta) \frac{\rho}{\sqrt{1 + \tan^2(\theta) + \tan^2(\psi)}} = x \tan(\theta), \tag{2}$$

$$z = \tan(\psi) \frac{\rho}{\sqrt{1 + \tan^2(\theta) + \tan^2(\psi)}} = x \tan(\psi); \tag{3}$$

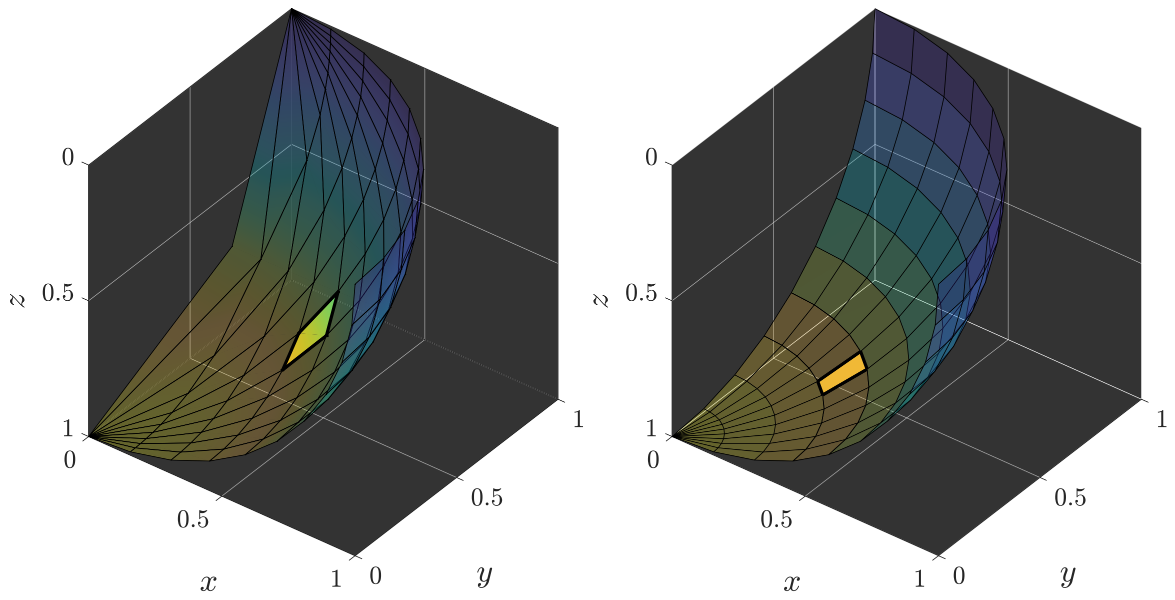

(a) Projected-elevation spherical coordinates. **(b)** Spherical coordinates.

Supplementary Figure 2: Comparison of hidden scene surface elements. The hidden scene surface elements generated from uniformly discretising the two angular coordinates in the proposed projected-elevation spherical coordinate system and the standard spherical coordinate system are shown in (a) and (b), respectively. A single fixed range, $\rho = 1$ m, is used. For each case, a 10-by-10 discretization is shown with the black lines indicating the boundaries of an elemental surface. Using a column-major indexing surface element $n = 57$ (i.e., the elemental surface at (projected-)elevation angle bin 7 and azimuthal angle bin 6) is highlighted. This element is identified by its centre $(\rho, \theta, \psi) = (1, {}^{11\pi}/_{40}, {}^{13\pi}/_{40})$ and its angular extents are ${}^{\pi}/_{20}$ along the azimuth and projected-elevation axes. A rotating 3D visualisation is provided in Supplementary Movie 2. (The colours in this visualization hold no meaning; they are used to aid visual interpretation of the 3D plot.)

and vice versa, using:

$$\rho^2 = x^2 + y^2 + z^2, \tag{4}$$

$$\psi = \arctan\left(z/x\right), \tag{5}$$

$$\theta = \arctan\left(y/x\right). \tag{6}$$

S1.3 Hidden Scene Representation Model

The hidden scene is modelled as a collection of surface elements formed by uniformly discretizing the azimuthal and projected-elevation axes of the projected-elevation spherical coordinate system. The range axis is not discretized, thus each surface element is associated with an arbitrary continuous-valued range (to be estimated). To achieve this discretization, the azimuthal projected-elevation axes are each uniformly divided into N_θ and N_ψ angular bins, respectively, with corresponding widths of $2\delta_\theta$ and $2\delta_\psi$. Thus, for an N_θ-by-N_ψ uniform discretization of a region $\{[0, \theta_{\max}] \times [0, \psi_{\max}]\}$ in the projected-elevation spherical coordinate system, the surface element at the i-th azimuthal and j-th projected-elevation angle position is centred at

$$\left(\frac{2i-1}{2N_\theta}\theta_{\max}, \frac{2j-1}{2N_\theta}\psi_{\max}\right),$$

for $i = 1, 2, \ldots, N_\theta$ and $j = 1, 2, \ldots, N_\psi$, with azimuthal and projected-elevation angular extents equal to $2\delta_\theta = {}^1\!/\!_{N_\theta}$ and $2\delta_\psi = {}^1\!/\!_{N_\psi}$, respectively. Using a colexicographical ordering[1] the n-th elemental surface denoted by $\mathcal{S}_n$, where $n = 1, 2, \ldots, N_\theta N_\psi$, assumed to have an unknown continuous-valued range ρ_n is the region:

$$\mathcal{S}_n = \{(\rho_n, \theta, \psi) : \theta \in [\theta_n - \delta_\theta, \theta_n + \delta_\theta],\ \text{and}\ \psi \in [\psi_n - \delta_\psi, \psi_n + \delta_\psi]\}, \tag{7}$$

where its centre is

$$\mathbf{s}_n = (\rho_n, \theta_n, \psi_n) = \left(\rho_n, \frac{2\lfloor (n-1)/N_\psi \rfloor + 1}{2N_\theta}\theta_{\max}, \frac{2(n - N_\psi\lfloor (n-1)/N_\psi \rfloor) - 1}{2N_\psi}\psi_{\max}\right), \tag{8}$$

where $\lfloor\cdot\rfloor$ denotes the floor operation. To produce a clear visualisation, the case where the octant $(\theta, \psi) \in \{[0, \pi/2] \times [0, \pi/2]\}$ is divided into $N_\theta = N_\psi = 10$ uniform regions is shown in Supplementary Figures 2 and 3. All $N_\theta N_\psi = 100$ surface elements are visualised in Supplementary Figure 2, while a close-up of a representative surface element $\mathcal{S}_{57}$) is shown in Supplementary Figure 3.

However, with fine enough discretization (i.e., high enough N_θ and N_ψ) this representation is rich enough to model a variety of scenes as a collection of possible surface elements $\{\mathcal{S}_1, \mathcal{S}_2, \ldots, \mathcal{S}_{N_\theta N_\psi}\}$ with corresponding unknown continuous-valued non-negative ranges $\{\rho_1, \rho_2, \ldots, \rho_{N_\theta N_\psi}\}$ and radiosities $\{c_1, c_2, \ldots, c_{N_\theta N_\psi}\}$. An arbitrary hidden scene can be represented by choosing appropriate c_n's and ρ_n's. Our goal will be to estimate the unknown c_n's and ρ_n's from a single measurement (i.e., a photograph of the ceiling plane).

S1.4 The Projected-Elevation Spherical Coordinates Light Transport Model

We now derive the contribution of a representative surface element $\mathcal{S}_n$, with range ρ_n and constant radiosity c_n, to the measurement plane photographs. To begin, we first state a model for the radiosity of a visible surface point $\mathbf{p}$ due to illumination from a hidden scene point. Let any hidden scene point $\mathbf{s} = (\rho, \theta, \psi)$ in 3D be represented by its range ρ from the origin, its azimuthal angle θ and projected-elevation angle ψ; denote also its equivalent Cartesian representation as $\mathbf{s}^{\mathrm{c}} = (s_x, s_y, s_z) = \left(\rho(1 + \tan^2\theta + \tan^2\psi)^{-1/2}, \rho\tan\theta(1 + \tan^2\theta + \tan^2\psi)^{-1/2}, \rho\tan\psi(1 + \tan^2\theta + \tan^2\psi)^{-1/2}\right)$. It is well known that the radiosity of a point $\mathbf{p}$ is equal to the sum of all incident light on the point multiplied by the albedo of the point $\mathbf{p}$. The light incident on $\mathbf{p}$ (where $\mathbf{p}$ is in Cartesian coordinates) originating from the hidden scene point $\mathbf{s}^{\mathrm{c}}$ whose radiosity is c is the product of: *(i)* the radiosity c of the point $\mathbf{s}^{\mathrm{c}}$; *(ii)* the inverse square-law decay in intensity with increasing distance between hidden scene point $\mathbf{s}^{\mathrm{c}}$ and visible scene point $\mathbf{p}$; *(iii)* a model for foreshortening and Lambertion reflection between $\mathbf{s}^{\mathrm{c}}$ and $\mathbf{p}$; and *(iv)* a model for any effects of occlusion between $\mathbf{s}^{\mathrm{c}}$ and $\mathbf{p}$. Accounting for these effects give

$$\ell(\mathbf{p}; \rho, \theta, \psi) = c\frac{\cos(\angle(\mathbf{p} - \mathbf{s}^{\mathrm{c}}, \mathbf{n_p}))}{\|\mathbf{p} - \mathbf{s}^{\mathrm{c}}\|_2^2} u\big(\theta - \tan^{-1}(p_x/p_y)\big)\, u\big(\psi - \tan^{-1}(h/p_y)\big), \tag{9}$$

as a model for the light incident on the visible ceiling point $\mathbf{p} = (p_x, p_y, -h)$ due to the hidden scene point $\mathbf{s}^{\mathrm{c}} = (c_x, c_y, c_z)$, where the ceiling plane is assumed to be a distance h above the horizontal edge of the doorway, while p_x and p_y represent the position of the point on the ceiling plane along the x- and y-axes. Moreover, the factor $u\big(\theta - \tan^{-1}(p_x/p_y)\big)$ models the effect of the occluding sidewall (i.e., the wall portion beside the vertical edge), while $u\big(\psi - \tan^{-1}(h/p_y)\big)$ models the effect of the occluding doorway head (i.e., the wall portion above the horizontal edge).

The light incident on $\mathbf{p}$ from the surface element $\mathcal{S}_n$ with unknown continuous-valued range $\rho_n \geq 0$ and unknown continuous-valued radiosity $c_n \geq 0$ is the integral of (9) over the entire surface $\mathcal{S}_n$. Thus,

$$\int_{\mathbf{s}\in\mathcal{S}_n} \ell(\mathbf{p}; \mathbf{s})\, \delta(\rho - \rho_n) \frac{\rho^2 \sec^2(\theta)\sec^2(\psi)}{(1 + \tan^2(\theta) + \tan^2(\psi))^{\frac{3}{2}}}\, \mathrm{d}\mathbf{s}, \tag{10}$$

[1] Also known as column-major indexing.

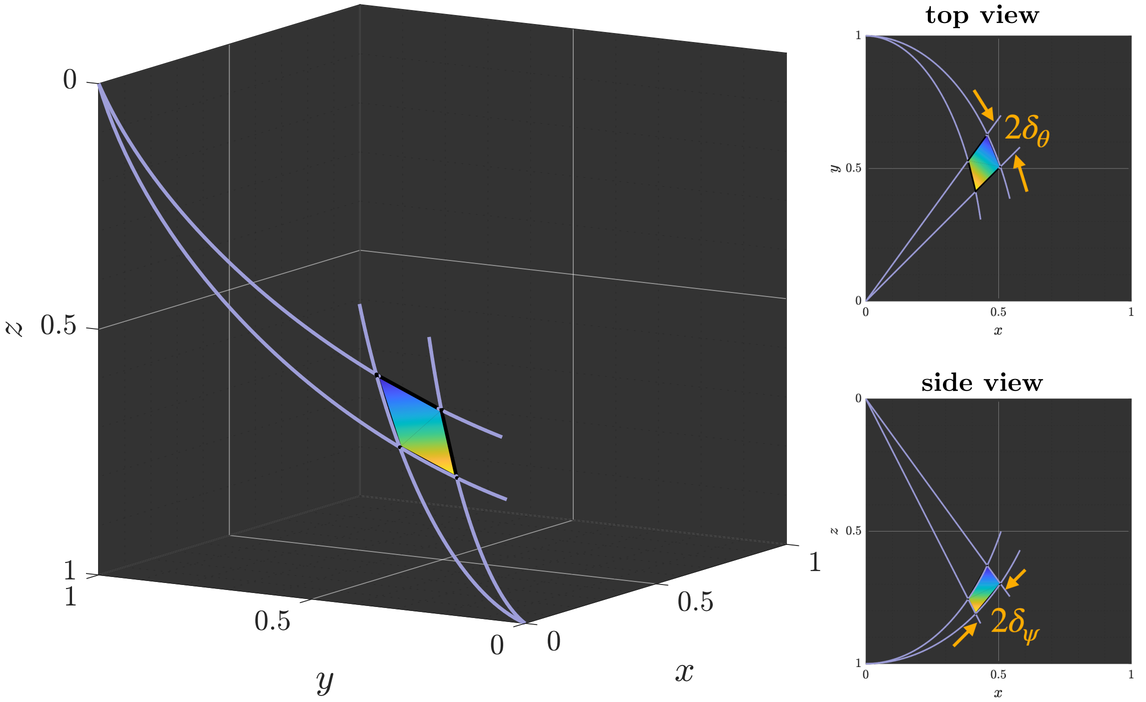

(a) Projected-elevation spherical coordinates. **(b)** Spherical coordinates.

Supplementary Figure 3: A single surface element. Close-up 3D (a), plan (b, top), and side (b, bottom) views of the representative projected-elevation spherical coordinate surface element highlighted in Supplementary Figure 2(a) (and main manuscript Figure 1(c)). The surface element has azimuthal and projected-elevation angular widths equal to $2\delta_\theta$ and $2\delta_\psi$, respectively, as indicated in plots (b). (The colour of the surface element holds no particular meaning; it is used to aid visual interpretation of the 3D plot.)

where the second factor in the integral accounts for the surface element having a single range ρ_n, and the third factor is the determinant of the Jacobian that arises when changing from Cartesian to the proposed projected-elevation spherical coordinates.

Let any measured ceiling photograph be of size $M_{\text{rows}} \times M_{\text{cols}}$ with $M = M_{\text{rows}} M_{\text{cols}}$

pixels resolution. Furthermore, assuming a uniform ceiling albedo—and because all pixels have an equal projected area on the ceiling plane—when the camera's focal plane is focussed on and fronto-parallel with the ceiling plane, the contribution $\ell_{m,n}$ of the n-the surface element to the measurement at the m-th camera pixel is proportional to (10) evaluated at the center of the m-th camera pixel location on the ceiling plane (i.e., $\mathbf{p} = \mathbf{p}_m = (p_{m,x}, p_{m,y}, -h)$). This gives,

$$\ell_{m,n} \propto \int_{\mathbf{s}\in\mathcal{S}_n} \ell\left(\mathbf{p}_m;\mathbf{s}\right)\delta(\rho-\rho_n)\frac{\rho^2\sec^2(\theta)\sec^2(\psi)}{(1+\tan^2(\theta)+\tan^2(\psi))^{\frac{3}{2}}}\,\mathrm{d}\mathbf{s} \tag{11}$$

$$= \int_{\mathbf{s}\in\mathcal{S}_n} \kappa c_n \delta(\rho-\rho_n)\frac{\cos\left(\angle(\mathbf{p}_m-\mathbf{s}^{\mathrm{c}},\mathbf{n}_{\mathbf{p}_m})\right)}{\|\mathbf{p}_m-\mathbf{s}^{\mathrm{c}}\|_2^2}\frac{\rho^2\sec^2(\theta)\sec^2(\psi)u\big(\theta-\tan^{-1}(p_{m,x}/p_{m,y})\big)\,u\big(\psi-\tan^{-1}(h/p_{m,y})\big)}{(1+\tan^2(\theta)+\tan^2(\psi))^{\frac{3}{2}}}\,\mathrm{d}\mathbf{s} \tag{12}$$

$$= \int_{\theta_n-\delta_\theta}^{\theta_n+\delta_\theta}\int_{\psi_n-\delta_\psi}^{\psi_n+\delta_\psi} \kappa c_n \frac{\cos\left(\angle(\mathbf{p}_m-\mathbf{s}^{\mathrm{c}},\mathbf{n}_{\mathbf{p}_m})\right)}{\|\mathbf{p}_m-\mathbf{s}^{\mathrm{c}}\|_2^2}\frac{\rho_n^2\sec^2(\theta)\sec^2(\psi)u\big(\theta-\tan^{-1}(p_{m,x}/p_{m,y})\big)\,u\big(\psi-\tan^{-1}(h/p_{m,y})\big)}{(1+\tan^2(\theta)+\tan^2(\psi))^{\frac{3}{2}}}\,\mathrm{d}\psi\mathrm{d}\theta, \tag{13}$$

where κ is the constant of proportionality. In addition, for sufficiently small surface elements, the varia-

tions in the radial falloff and cosine factors of the integrand with θ and ψ are negligible. Thus,

$$\ell_{m,n} \approx \kappa c_n \frac{\cos\left(\angle(\mathbf{p}_m - \mathbf{s}_n^{\mathrm{c}}, \mathbf{n}_{\mathbf{p}_m})\right)}{\|\mathbf{p}_m - \mathbf{s}_n^{\mathrm{c}}\|_2^2} \int_{\theta_n-\delta_\theta}^{\theta_n+\delta_\theta} \int_{\psi_n-\delta_\psi}^{\psi_n+\delta_\psi} \frac{\rho_n^2 \sec^2(\theta)\sec^2(\psi) u\big(\theta - \tan^{-1}(p_{m,x}/p_{m,y})\big)\, u\big(\psi - \tan^{-1}(h/p_{m,y})\big)}{(1+\tan^2(\theta)+\tan^2(\psi))^{\frac{3}{2}}} \,\mathrm{d}\psi \mathrm{d}\theta, \tag{14}$$

with $\mathbf{s}_n^{\mathrm{c}} = (s_{n,x}, s_{n,y}, s_{n,z})$ is the Cartesian representation of the projected-elevation spherical coordinate $\mathbf{s}_n = (\rho_n, \theta_n, \psi_n)$. For a collection of $N_\theta N_\psi$ surface elements $\{\mathcal{S}_1, \mathcal{S}_2, \ldots, \mathcal{S}_{N_\theta N_\psi}\}$ with corresponding non-negative ranges $\{\rho_1, \rho_2, \ldots, \rho_{N_\theta N_\psi}\}$ and radiosities $\{c_1, c_2, \ldots, c_{N_\theta N_\psi}\}$, the measurement y_m of camera pixel m is a linear combination of the contributions from all surface elements; that is

$$y_m = \sum_{n=1}^{N_\theta N_\psi} \kappa c_n \ell_{m,n} = \sum_{n=1}^{N_\theta N_\psi} f_n \ell_{m,n}, \tag{15}$$

where estimates of $\{\rho_1, \rho_2, \ldots, \rho_{N_\theta N_\psi}\}$ and scaled radiosities $\{f_1, f_2, \ldots, f_{N_\theta N_\psi}\}$ are desired from measurements $\{y_m\}_{m=1}^M$. Collecting the measurements $\{y_m\}_{m=1}^M$, ranges $\{\rho_1, \rho_2, \ldots, \rho_{N_\theta N_\psi}\}$, and radiosities $\{f_1, f_2, \ldots, f_{N_\theta N_\psi}\}$ into corresponding column vectors $\mathbf{y} \in \mathbb{R}^M$, $\mathbf{f} \in \mathbb{R}^{N_\theta N_\psi}$ and $\boldsymbol{\rho} \in \mathbb{R}^{N_\theta N_\psi}$, and incorporating measurement noise and visible scene illumination contributions, we can write the compact matrix-vector equation:

$$\mathbf{y} = \mathbf{A}(\boldsymbol{\rho})\mathbf{f} + \mathbf{v} + \mathbf{n}, \tag{16}$$

where element (m, n) of the matrix $\mathbf{A}(\boldsymbol{\rho}) \in \mathbb{R}^{M \times N_\theta N_\psi}$ is given by $\ell_{m,n}$ in (14), $\mathbf{v} \in \mathbb{R}^M$ denotes visible scene ambient light contributions, and $\mathbf{n} \in \mathbb{R}^M$ denotes zero-mean additive white Gaussian noise.

The projected-elevation spherical coordinates parameterization makes it possible to evaluate (14) in closed form, specifically,

$$\begin{aligned} \ell_{m,n} = \kappa c_n \frac{\cos\left(\angle(\mathbf{p}_m - \mathbf{s}_n^{\mathrm{c}}, \mathbf{n}_{\mathbf{p}_m})\right)}{\|\mathbf{p}_m - \mathbf{s}_n^{\mathrm{c}}\|_2^2} \Bigg[& \tan^{-1}\left(\frac{\sqrt{\tan^2\gamma_- + \sec^2\beta_+}}{\tan\beta_+ \tan\gamma_-}\right) - \tan^{-1}\left(\frac{\sqrt{\tan^2\gamma_- + \sec^2\beta_-}}{\tan\gamma_- \tan\beta_-}\right) \\ & - \tan^{-1}\left(\frac{\sqrt{\tan^2\gamma_+ + \sec^2\beta_+}}{\tan\gamma_+ \tan\beta_+}\right) + \tan^{-1}\left(\frac{\sqrt{\tan^2\gamma_+ + \sec^2\beta_-}}{\tan\gamma_+ \tan\beta_-}\right) \Bigg], \end{aligned} \tag{17}$$

where

$$\gamma_\pm = \max\left(\psi_n \pm \delta_\psi, \tan^{-1}(h/p_{m,y})\right), \text{ and} \tag{18a}$$

$$\beta_\pm = \max\left(\theta_n \pm \delta_\theta, \tan^{-1}(p_{m,x}/p_{m,y})\right). \tag{18b}$$

S1.5 Computational Field of View

Certain regions of the hidden scene have poor conditioning due to the nature of the occlusion. In particular, there are two regions that have significantly worse conditioning than the rest of the hidden scene. First, there is a region that is fully occluded by the door head. Because there are no unoccluded paths from this region to the observation plane, this region is completely outside the computational field of view (CFOV) and is impossible to recover. The shape of this region (which depends on h and the size of the image) is shown in Supplementary Figure 4. If we let w be the width of the measurement plane, and h be the distance from the horizontal edge to the measurement plane, this region is described by $\psi \leq \tan^{-1}(\frac{h}{w})$.

Second, as shown in Supplementary Figure 5, for regions where θ is close to $\pi/2$ and ψ is large, while they are not completely occluded, the slanted penumbra boundary in the camera measurements that gives high-fidelity information about the azimuth angle of the hidden scene target is occluded by the horizontal angle. Thus, varying θ provides little change, forming a region of poor conditioning. This region is illustrated by the uncertainty region in Supplementary Figure 7, in the top view of the two-edge case (top-right image). The regions where θ is close to $\pi/2$ are circular, whereas the rest of the uncertainty

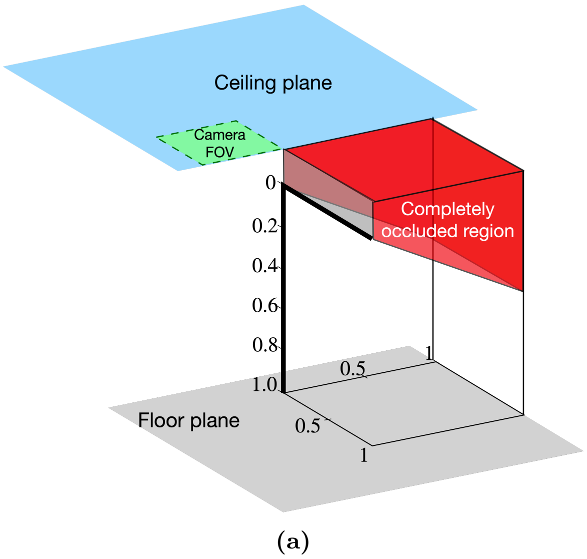

Supplementary Figure 4: Completely occluded portion of the hidden scene given a camera FOV. (a) The red region shows the portion of the hidden scene that produces no light contribution to the observation photograph due to being occluded completely by the door head (horizontal occluding edge); while the green area of the ceiling plane indicates the camera's FOV. (b) Overhead light illuminating the hidden scene area is completely occluded by the doorway edge from directly illuminating the visible scene ceiling plane, however, the hidden scene mannequin reflects incoming light into the camera's FOV.

regions have collapsed to a line. By simple geometric computations, it follows that this region of poor conditioning occurs when $\theta \geq \tan^{-1}(\frac{w\tan(\psi)}{h})$.

These regions of uncertainty grow smaller with increasing w and decreasing h, so we conclude that having a large observation image but a small h is desirable to maximize the recoverable hidden scene portion (i.e., the CFOV). Of course, $h = 0$ reduces the configuration to a single edge case, so h can't be too close to 0.

To facilitate the formation of a well-conditioned system of equations, we do not attempt to recover these totally occluded regions.

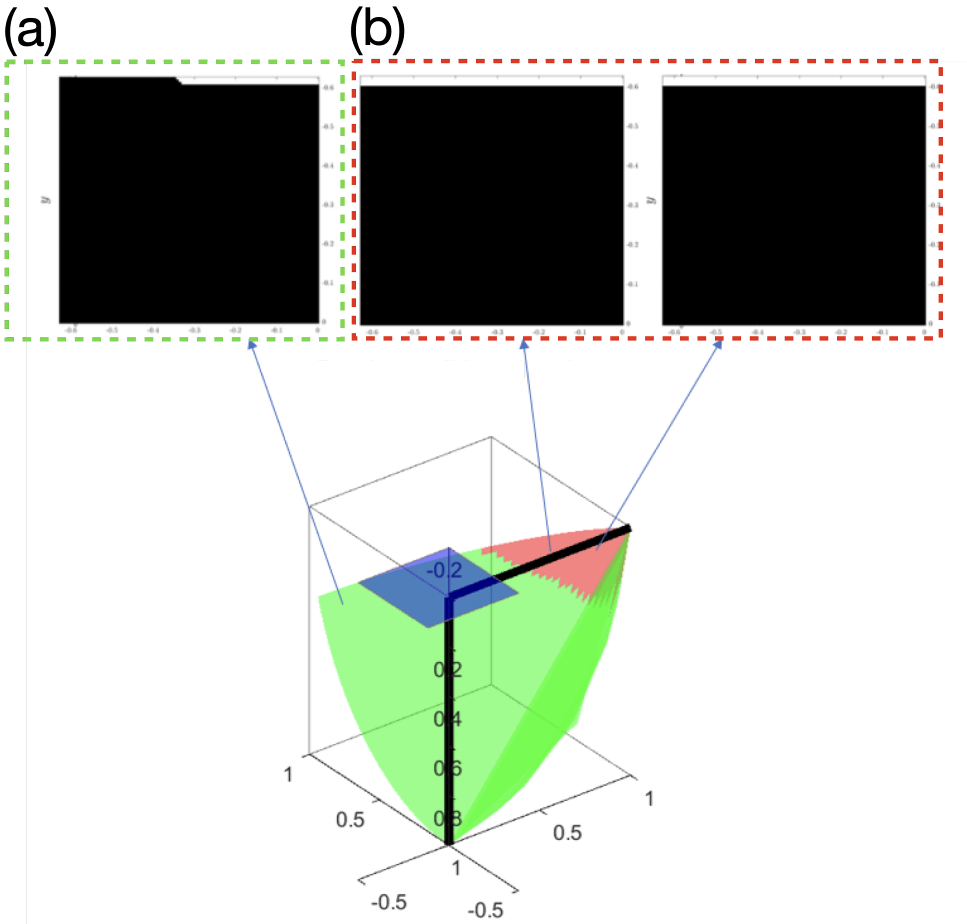

Supplementary Figure 5: Region of poor conditioning. Surface elements with poor conditioning are indicated in red colour. Their poor conditioning results from having a very similar penumbra pattern with at least one other surface element, meaning the only observable illumination differences are due to radial falloff or foreshortening factors. Surface elements in the solid angle described by the green region are, by contrast, well conditioned since they cast a unique penumbra pattern onto the measurement plane (the range has no effect on the penumbra). (a) Shows the penumbra pattern of a well condition surface element; notice the slanted edge of the illuminated region in white which is informative of the azimuthal angle of the surface element. (b) The measured penumbra patterns of two poorly conditioned surface elements appear almost the same.

Supplementary Note 2

S2 Fisher Information and Cramér-Rao Bound Analyses

The aim of the proposed approach is to form an accurate reconstruction of the hidden scene from a single observation photograph. Mathematically, this amounts to estimating $(\mathbf{f}, \boldsymbol{\rho})$ in (16) from measurements $\mathbf{y}$. Here, a formal study of the estimability of the hidden scene through a number of Fisher Information (FI) and Cramér-Rao Bound (CRB) analyses is presented. CRB-based comparisons among different acquisition configurations and hidden scene parameterizations are explored.

The CRBs of the no-edge, single-edge (vertical), and two-edge (vertical and horizontal) acquisition configurations are used to further justify the utility of the additional orthogonal edge in the proposed two-edge configuration. For an additive Gaussian noise measurement model $y_m = i_m + n_m$, where $n_m \sim \mathcal{N}(0, \sigma^2)$. Let the Fisher information (FI) matrix be denoted by $\mathbf{I}$ then the CRB is $\mathbf{I}^{-1}$. We derive the FI matrices and CRBs for the general two-edge imaging system and then for the one-edge and two-edge systems, as special cases in what follows.

Consider a hidden scene point whose radiosity is c and position is $\mathbf{s} = (\rho, \theta, \psi)$ in projected-elevation spherical coordinates. The measurement $i_m(\rho, \theta, \psi)$ observed by the m-th camera pixel is (approximately) equal to (9) integrated over the patch $\mathcal{P}_m$ which depicts the projected area of the camera pixel onto the measurement plane:

$$i_m(\rho,\theta,\psi) = \int_{\mathbf{p}\in\mathcal{P}_m} \ell\left(\mathbf{p};\rho,\theta,\psi\right)\,\mathrm{d}\mathbf{p} \tag{19}$$

$$= c\int_{\mathbf{p}\in\mathcal{P}_m} \frac{\cos\left(\angle(\mathbf{p}-\mathbf{s}^{\mathrm{c}},\mathbf{n}_{\mathbf{p}})\right)}{\|\mathbf{p}-\mathbf{s}^{\mathrm{c}}\|_2^2} u\big(\theta-\tan^{-1}(p_x/p_y)\big)\,u\big(\psi-\tan^{-1}(h/p_y)\big)\,\mathrm{d}\mathbf{p} \tag{20}$$

$$\approx c\frac{\cos\left(\angle(\mathbf{p}_m-\mathbf{s}^{\mathrm{c}},\mathbf{n}_{\mathbf{p}_m})\right)}{\|\mathbf{p}_m-\mathbf{s}^{\mathrm{c}}\|_2^2}\int_{\mathbf{p}\in\mathcal{P}_m} u\big(\theta-\tan^{-1}(p_x/p_y)\big)\,u\big(\psi-\tan^{-1}(h/p_y)\big)\,\mathrm{d}\mathbf{p} \tag{21}$$

$$= \alpha c r\int_{\mathbf{p}\in\mathcal{P}_m} u\big(\theta-\tan^{-1}(p_x/p_y)\big)\,u\big(\psi-\tan^{-1}(h/p_y)\big)\,\mathrm{d}\mathbf{p}, \tag{22}$$

the final expression follows because the variations in the foreshortening and radial falloff factors over the relatively small pixel patch area $\mathcal{P}_m$ are inconsequential. Where it has been assumed that $\alpha = \cos\left(\angle(\mathbf{p}_m - \mathbf{s}^{\mathrm{c}}, \mathbf{n}_{\mathbf{p}_m})\right)$ is constant over the entire patch, while

$$r = \|\mathbf{p}_m-\mathbf{s}^{\mathrm{c}}\|_2^{-2} = \frac{1}{\rho^2 + 2\gamma\rho\left(p_{m,y}+p_{m,x}\tan\theta + h\tan\psi\right) + p_{m,x}^2 + p_{m,y}^2 + h^2},$$

with $\gamma = (1+\tan^2\theta+\tan^2\psi)^{-1/2}$.

The derivatives of (22) with respect to the hidden scene point's radiosity c and positional parameters (ρ, θ, ψ) are desired. These derivatives are computed as follows:

$$\frac{\partial i_m(\rho,\theta,\psi)}{\partial c} = \frac{\alpha}{\|\mathbf{p}_m-\mathbf{s}^{\mathrm{c}}\|_2^2}\int_{\mathbf{p}\in\mathcal{P}_m} u\big(\theta-\tan^{-1}(p_x/p_y)\big)\,u\big(\psi-\tan^{-1}(h/p_y)\big)\,\mathrm{d}\mathbf{p}, \tag{23}$$

$$\frac{\partial i_m(\rho,\theta,\psi)}{\partial \rho} = \alpha c\frac{\partial r}{\partial \rho}\int_{\mathbf{p}\in\mathcal{P}_m} u\big(\theta-\tan^{-1}(p_x/p_y)\big)\,u\big(\psi-\tan^{-1}(h/p_y)\big)\,\mathrm{d}\mathbf{p}, \tag{24}$$

where $\frac{\partial r}{\partial \rho} = -2r^2\left(\rho + \gamma\left(p_{m,y}+p_{m,x}\tan\theta + h\tan\psi\right)\right)$. Further, the derivatives with respect to the angu-

lar axes are

$$
\begin{aligned}
\frac{\partial i_m(\rho,\theta,\psi)}{\partial\theta} &= \alpha c r \int_{\mathbf{p}\in\mathcal{P}_m} \frac{\partial}{\partial\theta} u\big(\theta-\tan^{-1}(p_x/p_y)\big)\, u\big(\psi-\tan^{-1}(h/p_y)\big)\ \mathrm{d}\mathbf{p} \\
&\quad + \alpha c \frac{\partial r}{\partial\theta} \int_{\mathbf{p}\in\mathcal{P}_m} u\big(\theta-\tan^{-1}(p_x/p_y)\big)\, u\big(\psi-\tan^{-1}(h/p_y)\big)\ \mathrm{d}\mathbf{p} \\
&= \alpha c r \int_{\mathbf{p}\in\mathcal{P}_m} \delta\big(\theta-\tan^{-1}(p_x/p_y)\big)\, u\big(\psi-\tan^{-1}(h/p_y)\big)\ \mathrm{d}\mathbf{p} \\
&\quad + \alpha c \frac{\partial r}{\partial\theta} \int_{\mathbf{p}\in\mathcal{P}_m} u\big(\theta-\tan^{-1}(p_x/p_y)\big)\, u\big(\psi-\tan^{-1}(h/p_y)\big)\ \mathrm{d}\mathbf{p},
\end{aligned} \tag{25}
$$

where $\frac{\partial r}{\partial\theta} = -2\gamma\rho r^2 \left(p_{m,x}\sec^2\theta - \gamma^2(p_{m,y} + p_{m,x}\tan\theta + h\tan\psi)\tan\theta\sec^2\theta\right)$, and finally

$$
\begin{aligned}
\frac{\partial i_m(\rho,\theta,\psi)}{\partial\psi} &= \alpha c r \int_{\mathbf{p}\in\mathcal{P}_m} u\left(\theta-\tan^{-1}(p_x/p_y)\right) \frac{\partial}{\partial\psi} u\left(\psi-\tan^{-1}(h/p_y)\right)\ \mathrm{d}\mathbf{p} \\
&\quad + \alpha c \frac{\partial r}{\partial\psi} \int_{\mathbf{p}\in\mathcal{P}_m} u\left(\theta-\tan^{-1}(p_x/p_y)\right) u\left(\psi-\tan^{-1}(h/p_y)\right)\ \mathrm{d}\mathbf{p} \\
&= \alpha c r \int_{\mathbf{p}\in\mathcal{P}_m} u\left(\theta-\tan^{-1}(p_x/p_y)\right) \delta\left(\psi-\tan^{-1}(h/p_y)\right)\ \mathrm{d}\mathbf{p} \\
&\quad + \alpha c \frac{\partial r}{\partial\psi} \int_{\mathbf{p}\in\mathcal{P}_m} u\left(\theta-\tan^{-1}(p_x/p_y)\right) u\left(\psi-\tan^{-1}(h/p_y)\right)\ \mathrm{d}\mathbf{p},
\end{aligned} \tag{26}
$$

where $\frac{\partial r}{\partial\psi} = -2\gamma\rho r^2 \left(h\sec^2\psi - \gamma^2(p_{m,y} + p_{m,x}\tan\theta + h\tan\psi)\tan\psi\sec^2\psi\right)$. In (25) and (26) the derivatives of the unit step functions are taken in the distributional sense to obtain Dirac delta functions.

Assuming an M-pixel measurement photograph and additive white Gaussian measurement noise model, that is $y_m = i_m + n_m$, where $n_m \sim \mathcal{N}(0,\sigma^2)$ for $m = 1,2,\ldots,M$, then the Fisher information (FI) matrix for the proposed *two-edge imaging configuration* is given by:

$$
\mathbf{I}_{2\mathrm{E}} = \frac{1}{\sigma^2}\left(\nabla\mathbf{i}^\top\nabla\mathbf{i}\right), \tag{27}
$$

where

$$
\nabla\mathbf{i} = \begin{bmatrix}
\frac{\partial i_1}{\partial c} & \frac{\partial i_1}{\partial\rho} & \frac{\partial i_1}{\partial\theta} & \frac{\partial i_1}{\partial\psi} \\
\frac{\partial i_2}{\partial c} & \frac{\partial i_2}{\partial\rho} & \frac{\partial i_2}{\partial\theta} & \frac{\partial i_2}{\partial\psi} \\
\vdots & \vdots & \vdots & \vdots \\
\frac{\partial i_M}{\partial c} & \frac{\partial i_M}{\partial\rho} & \frac{\partial i_M}{\partial\theta} & \frac{\partial i_M}{\partial\psi}
\end{bmatrix}, \tag{28}
$$

is an $M\times 4$ matrix of the derivatives computed in (23), (24), (25) and (26) above.

For comparison, the no-edge and one-edge configuration FI matrices, $\mathbf{I}_{0\mathrm{E}}$ and $\mathbf{I}_{1\mathrm{E}}$ respectively, are also computed. These configurations can be seen as special cases of the two-edge configuration.

S2.1 Special case I: No-edge configuration

In this case, without any occluding edges, all points are visible to the ceiling plane. Thus, without any dependence on angles θ and ψ, the visibility function reduces trivially to unity. The partial derivatives in this case become:

$$\frac{\partial i_m}{\partial c} = \alpha r \tag{29}$$

$$\frac{\partial i_m}{\partial \rho} = \alpha c \frac{\partial r}{\partial \rho} \tag{30}$$

$$\frac{\partial i_m}{\partial \theta} = \alpha c \frac{\partial r}{\partial \theta} \tag{31}$$

$$\frac{\partial i_m}{\partial \psi} = \alpha c \frac{\partial i_m}{\partial \psi}, \tag{32}$$

where, as before, $\frac{\partial r}{\partial \rho} = -2r^2 \left(\rho + \gamma \left(p_{m,y} + p_{m,x} \tan\theta + h \tan\psi\right)\right)$, $\frac{\partial r}{\partial \theta} = -2\gamma\rho r^2 \left(p_{m,x} \sec^2\theta - \gamma^2 (p_{m,y} + p_{m,x} \tan\theta + h\tan\psi) \tan\theta \sec^2\theta\right)$, and $\frac{\partial r}{\partial \psi} = -2\gamma\rho r^2 \left(h \sec^2\psi - \gamma^2 (p_{m,y} + p_{m,x} \tan\theta + h\tan\psi) \tan\psi \sec^2\psi\right)$. With these derivatives the FI matrix $\mathbf{I}_{0\text{E}}$ is formed similarly to $\mathbf{I}_{2\text{E}}$ in (27).

S2.2 Special case II: One-edge configuration

For the vertical occluding edge case, it follows that the visibility function has no dependence on the projected-elevation angle component. This reduces the component of the visibility function that models the horizontal occluding edge to unity, leaving only the factor $u(\theta - \tan^{-1}(p_x/p_y))$, whose derivative (in the distributional sense) is $\delta(\theta - \tan^{-1}(p_x/p_y))$. Thus, the desired derivatives for this configuration are:

$$\frac{\partial i_m(\rho,\theta,\psi)}{\partial c} = \frac{\alpha}{\|\mathbf{p}_m - \mathbf{s}^{\text{c}}\|_2^2} \int_{\mathbf{p}\in\mathcal{P}_m} u\big(\theta - \tan^{-1}(p_x/p_y)\big)\, \mathrm{d}\mathbf{p}, \tag{33}$$

$$\frac{\partial i_m(\rho,\theta,\psi)}{\partial \rho} = \alpha c \frac{\partial r}{\partial \rho} \int_{\mathbf{p}\in\mathcal{P}_m} u\big(\theta - \tan^{-1}(p_x/p_y)\big)\, \mathrm{d}\mathbf{p} \tag{34}$$

$$\frac{\partial i_m(\rho,\theta,\psi)}{\partial \theta} = \alpha c r \int_{\mathbf{p}\in\mathcal{P}_m} \delta\big(\theta - \tan^{-1}(p_x/p_y)\big)\, \mathrm{d}\mathbf{p} + \alpha c \frac{\partial r}{\partial \theta} \int_{\mathbf{p}\in\mathcal{P}_m} u\big(\theta - \tan^{-1}(p_x/p_y)\big)\, \mathrm{d}\mathbf{p} \tag{35}$$

$$\frac{\partial i_m(\rho,\theta,\psi)}{\partial \psi} = \alpha c \frac{\partial r}{\partial \psi} \int_{\mathbf{p}\in\mathcal{P}_m} u\left(\theta - \tan^{-1}(p_x/p_y)\right)\, \mathrm{d}\mathbf{p}, \tag{36}$$

where the partial derivatives $\frac{\partial r}{\partial \rho}$, $\frac{\partial r}{\partial \theta}$, and $\frac{\partial r}{\partial \psi}$ are the same as before. With the derivatives above the FI matrix $\mathbf{I}_{1\text{E}}$ is formed similarly to $\mathbf{I}_{2\text{E}}$ in (27).

S2.3 One-edge vs Two-edge Configurations: A Crámer-Rao Bound Comparison

The CRB is a lower bound for the posterior variance of the estimated parameters. Given the Fisher information matrix $\mathbf{I}$ for the set of unknown parameters, their CRB is computed as the inverse of $\mathbf{I}$. Here, CRBs for estimating the unknown parameters c, ρ, θ, and ψ of a hidden scene point from noisy observations are visualized. To achieve this, the computed CRBs are converted from the projected-elevation spherical coordinates to Cartesian coordinates for plotting using:

$$\mathbf{C}_{\text{cart}} = \sigma^2 \left((\nabla \mathbf{i}\, \mathbf{J})^\top \nabla \mathbf{i}\, \mathbf{J} \right)^{-1} \tag{37}$$

where the Jacobian matrix $\mathbf{J}$ is

$$\mathbf{J} = \begin{bmatrix} \frac{\partial c}{\partial c} & \frac{\partial \rho}{\partial c} & \frac{\partial \theta}{\partial c} & \frac{\partial \psi}{\partial c} \\ \frac{\partial c}{\partial x} & \frac{\partial \rho}{\partial x} & \frac{\partial \theta}{\partial x} & \frac{\partial \psi}{\partial x} \\ \frac{\partial c}{\partial y} & \frac{\partial \rho}{\partial y} & \frac{\partial \theta}{\partial y} & \frac{\partial \psi}{\partial y} \\ \frac{\partial c}{\partial z} & \frac{\partial \rho}{\partial z} & \frac{\partial \theta}{\partial z} & \frac{\partial \psi}{\partial z} \end{bmatrix} = \begin{bmatrix} 1 & 0 & 0 & 0 \\ 0 & \frac{\partial \rho}{\partial x} & \frac{\partial \theta}{\partial x} & \frac{\partial \psi}{\partial x} \\ 0 & \frac{\partial \rho}{\partial y} & \frac{\partial \theta}{\partial y} & \frac{\partial \psi}{\partial y} \\ 0 & \frac{\partial \rho}{\partial z} & \frac{\partial \theta}{\partial z} & \frac{\partial \psi}{\partial z} \end{bmatrix}. \tag{38}$$

(39)

Here the desired derivatives are computed from the mappings from projected-elevation spherical coordinates to Cartesian coordinates given in (4), (5), and (6). Note that because the radiosity of the point is left untransformed by the change in spatial coordinates, its derivatives with respect to the positional parameters are zero, while the derivative with respect to itself is unity.

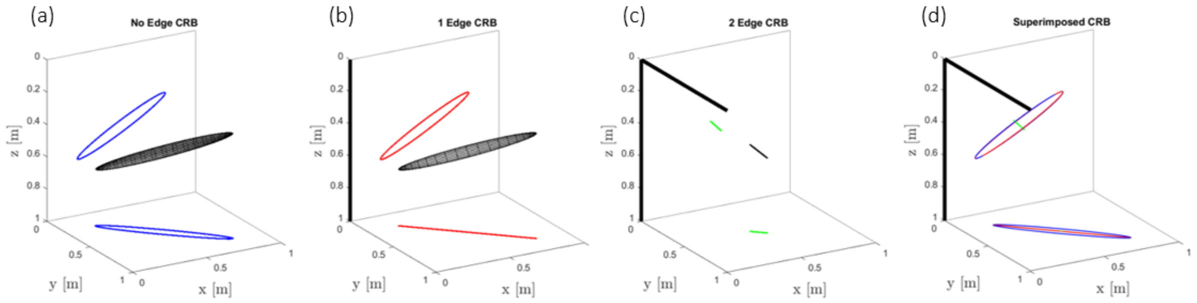

Supplementary Figure 6: Cramér-Rao Bound comparisons across various edge-resolved 3D imaging configurations. Visualized ellipsoids (black) show the 50% CRB uncertainty regions, i.e. the region wherein 50% of all estimates will fall when performing repeated trials; and the centres of the ellipsoids coincide with the true source location. Also shown are projections of the ellipsoidal regions onto the xy- and xz-planes for better appreciation of the uncertainties along those dimensions. (a) Shows the no-edge case. (b) Shows the one-edge case of the conventional passive corner cameras[1, 3, 4]. (c) Shows the two-edge configuration proposed in this work. (d) Shows the projected uncertainties in (a-c) superimposed on each other to facilitate visual comparison. In each case, the edge that would be exploited for imaging is shown in bold. The measurement SNR of 79 dB is held fixed across the three configurations.

First, the CRB for a point at the position $\mathbf{s} = (\sqrt{3}/2, \pi/4, \pi/4)$ (equivalently, $\mathbf{s}^{\mathrm{c}} = (0.5, 0.5, 0.5)$ in Cartesian coordinates) is computed and used to plot, in Supplementary Figure 6, the 50% confidence region for estimating its 3D position. This is achieved by computing the CRBs for the no-edge, vertical-edge, and two-edge cases for a fixed noise level σ, and plotting the region wherein 50% of estimates are expected to fall. In the no-edge case, the uncertainty region is, in general, an ellipsoid centred at the Cartesian location $(0.5, 0.5, 0.5)$. For the single edge case, the θ dimension is well resolved, collapsing the uncertainty region to an ellipse (precisely, it is a very thin ellipsoid). The two-edge case collapses the ellipse to a line pointing toward the origin. This suggests that when forming a 3D estimate using each of these configurations, the most accurate estimate will likely be obtained by the two-edge configuration.

Supplementary Figure 7 shows the uncertainty regions (projected onto the xy- and yz-planes) across multiple points in the hidden scene space: Specifically for equally spaced ranges, azimuth, and projected-elevations. This computation gives an idea of the computational field-of-view (CFOV)[2] of the proposed NLOS imaging configuration. In the plan view visualisations, the projected-elevation angle ψ is fixed, while the hidden scene points are varied in ρ and θ. Notice that although the uncertainty regions are quite small, suggesting good localization accuracy, the regions near the extremities of the hidden scene (for θ values close to 0 and $\pi/2$) have larger regions.

[2]Describes the portion of the hidden scene that is well-conditioned for recovery [9].

In the side view visualizations, azimuth θ is fixed while ψ and ρ are varied together. The uncertainty region becomes slightly wider for lower angles. Also, notice that the regions stop at a certain angle and don't increase above it. This is because the region above is completely occluded by the horizontal edge. Thus, we can gain no information in that region. This effect produces the computational field-of-view (CFOV) [9] of the NLOS imaging system which was explained in Section S1.5. The CFOV is the region of the hidden scene that is well-conditioned for recovery.

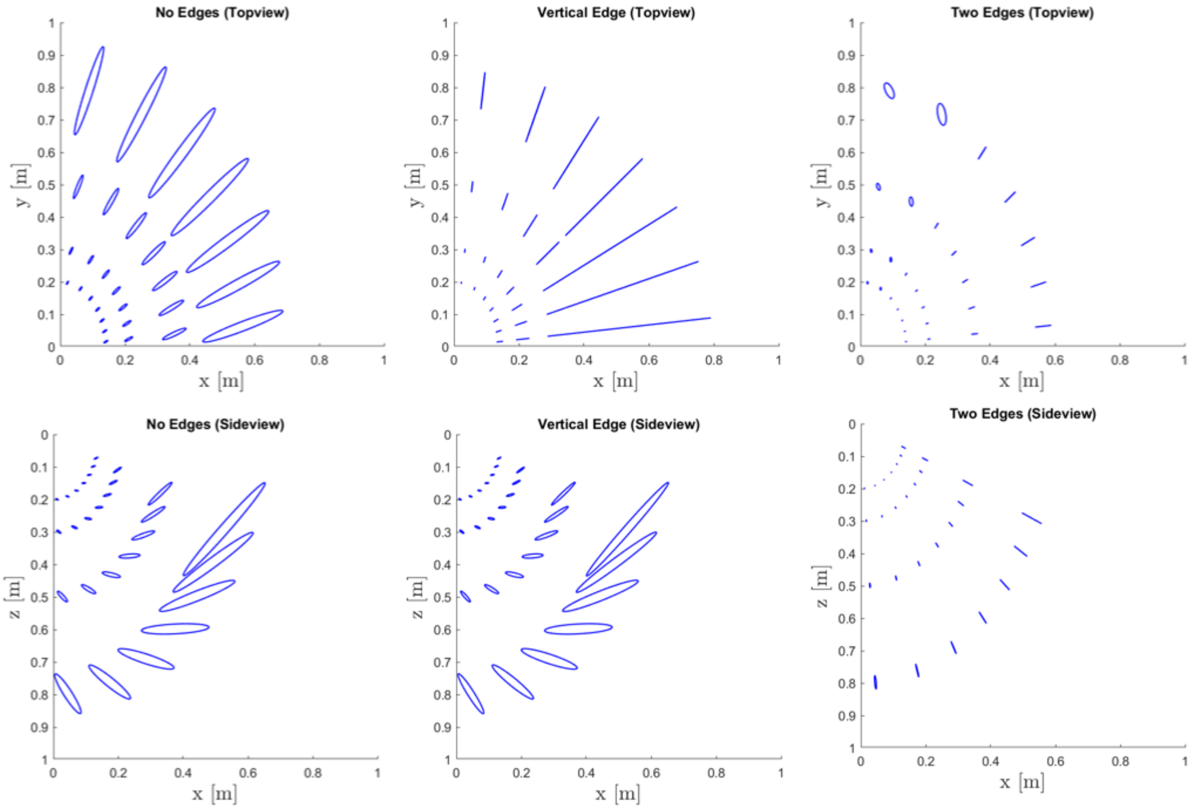

Supplementary Figure 7: 95 percent confidence regions with SNR = 85 dB for the no-edge, one-edge and two-edge case. The top row depicts the plan view (the 3D confidence region projected onto the xy-plane), with a fixed $\psi = \pi/4$ and varied ρ and θ. The bottom row shows the confidence region from the side, projected onto the xz-plane. Here, θ is fixed, and ρ and ψ vary. Notice ψ does not take up the whole hidden region because if the angle is too deep, any object there is completely occluded and has an infinite uncertainty region.

S2.4 Comparing Per Cluster Range vs Per Elemental Surface Range Estimation

Here we provide the theoretical, CRB-based, justification for clustering neighbouring elements and estimating a single range for the entire cluster, in lieu of estimating a range for each member of the cluster. To do so, an object situated at a distance $\bar{\rho}$ from the origin is considered. Then under the assumption that the first step of the proposed reconstruction procedure returns a cluster of K surface elements to represent the object, the CRB for the first case of recovering a single range estimate for the computed cluster of surface elements is derived in Section S2.4.1. Next, the CRB for the alternative of recovering K range estimates, i.e. one for each surface element of the cluster representing the object, is computed in Section S2.4.2. Finally, the derived CRBs for each case are compared in Section S2.4.3

S2.4.1 CRB for Estimating a Single Range Per Cluster of Surface Elements

Assuming Step 1 of the reconstruction algorithm has identified J clusters, without loss of generality let $J = 1$, and also let the cluster be comprised of K surface elements such that $\mathcal{C} = \bigcup_{k=1}^{K} \mathcal{S}_k$. Let the estimated scaled-radiosities be $\{\hat{c}_k\}_{k=1}^{K}$ correspondingly. We now evaluate the CRB of the scenario where the elements of the cluster are assumed to share one single range to be estimated. Under this set of assumptions, the contribution produced by the cluster in a measurement $y_m = h_m + n_m$ is:

$$h_m(w,\rho) = w \sum_{k=1}^{K} \hat{c}_k \int_{\mathbf{p}\in\mathcal{P}_m} \frac{\cos\left(\angle(\mathbf{p}-\mathbf{s}_k^{\mathrm{c}}, \mathbf{n}_{\mathbf{p}})\right)}{\|\mathbf{p}-\mathbf{s}_k^{\mathrm{c}}\|_2^2} u\big(\theta_k - \tan^{-1}(p_x/p_y)\big)\, u\big(\psi_k - \tan^{-1}(h/p_y)\big)\, \mathrm{d}\mathbf{p} \tag{40}$$

$$\approx d^2 w \sum_{k=1}^{K} \hat{c}_k \frac{\cos\left(\angle(\mathbf{p}_m-\mathbf{s}_k^{\mathrm{c}}, \mathbf{n}_{\mathbf{p}_m})\right)}{\|\mathbf{p}_m-\mathbf{s}_k^{\mathrm{c}}\|_2^2} \int_{\mathbf{p}\in\mathcal{P}_m} u\big(\theta_k - \tan^{-1}(p_x/p_y)\big)\, u\big(\psi_k - \tan^{-1}(h/p_y)\big)\, \mathrm{d}\mathbf{p} \tag{41}$$

$$= d^2 w \sum_{k=1}^{K} \alpha(\rho,\theta_k,\psi_k)\, r(\rho,\theta_k,\psi_k)\, \hat{c}_k \underbrace{\int_{\mathbf{p}\in\mathcal{P}_m} u\big(\theta_k - \tan^{-1}(p_x/p_y)\big)\, u\big(\psi_k - \tan^{-1}(h/p_y)\big)\, \mathrm{d}\mathbf{p}}_{\stackrel{\mathrm{def}}{=} O_{k,m}} \tag{42}$$

$$= d^2 w \sum_{k=1}^{K} \alpha(\rho,\theta_k,\psi_k)\, r(\rho,\theta_k,\psi_k)\, \hat{c}_k O_{k,m}, \tag{43}$$

where $\alpha(\rho,\theta_k,\psi_k) \stackrel{\mathrm{def}}{=} \cos\left(\angle(\mathbf{p}_m-\mathbf{s}_k^{\mathrm{c}}, \mathbf{n}_{\mathbf{p}_m})\right)$, $r(\rho,\theta_k,\psi_k) \stackrel{\mathrm{def}}{=} 1/\|\mathbf{p}_m-\mathbf{s}_k^{\mathrm{c}}\|_2^2$, and $n_m \sim \mathcal{N}(0,\sigma^2)$. Also, the range ρ_k for surface element k in the cluster is assumed to be ρ, thus $\rho_k = \rho$ for all $k = 1,\ldots,K$. The quantity d is the length of the square patch covered by each camera pixel (i.e., the projected size of a camera pixel on the measurement plane), and w is the overall scaling of the cluster. The derivatives of (43) with respect to the unknowns w and ρ are:

$$\frac{\partial h_m}{\partial w} \approx d^2 \sum_{k=1}^{K} \alpha(\rho,\theta_k,\psi_k)\, r(\rho,\theta_k,\psi_k)\, \hat{c}_k O_{k,m}, \tag{44}$$

and

$$\frac{\partial h_m}{\partial \rho} \approx d^2 w \sum_{k=1}^{K} \frac{\partial}{\partial \rho}\Big(\alpha(\rho,\theta_k,\psi_k)\, r(\rho,\theta_k,\psi_k)\Big)\, \hat{c}_k O_{k,m} \tag{45}$$

respectively. Next, given M observations $\{y_m\}_{m=1}^{M}$, the FI matrix for estimating the pair (w,ρ) from it is:

$$\mathbf{I}^{\mathcal{C}} = \frac{1}{\sigma^2} \begin{bmatrix} \frac{\partial h_1}{\partial w} & \frac{\partial i_1}{\partial \rho} \\ \frac{\partial h_2}{\partial w} & \frac{\partial i_2}{\partial \rho} \\ \vdots & \vdots \\ \frac{\partial h_M}{\partial w} & \frac{\partial i_M}{\partial \rho} \end{bmatrix}^{\top} \begin{bmatrix} \frac{\partial h_1}{\partial w} & \frac{\partial i_1}{\partial \rho} \\ \frac{\partial h_2}{\partial w} & \frac{\partial i_2}{\partial \rho} \\ \vdots & \vdots \\ \frac{\partial h_M}{\partial w} & \frac{\partial i_M}{\partial \rho} \end{bmatrix} \tag{46}$$

$$= \frac{1}{\sigma^2} \begin{bmatrix} \sum_{m=1}^{M} \big(\frac{\partial h_m}{\partial w}\big)^2 & \sum_{m=1}^{M} \frac{\partial h_m}{\partial \rho}\frac{\partial h_m}{\partial w} \\ \sum_{m=1}^{M} \frac{\partial h_m}{\partial w}\frac{\partial h_m}{\partial \rho} & \sum_{m=1}^{M} \Big(\frac{\partial h_m}{\partial \rho}\Big)^2 \end{bmatrix}. \tag{47}$$

Next, the entries of $\mathbf{I}^{\mathcal{C}}$ are computed explicitly, as:

$$\left[\mathbf{I}^{\mathcal{C}}\right]_{1,1} \approx \frac{d^4}{\sigma^2} \sum_{m=1}^{M} \left(\sum_{k=1}^{K} \alpha(\rho,\theta_k,\psi_k)\, r(\rho,\theta_k,\psi_k)\, \hat{c}_k O_{k,m}\right)^2, \tag{48}$$

$$\left[\mathbf{I}^{\mathcal{C}}\right]_{1,2} = \left[\mathbf{I}^{\mathcal{C}}\right]_{2,1} \approx \frac{d^4 w}{\sigma^2} \sum_{m=1}^{M} \sum_{k=1}^{K} \alpha(\rho,\theta_k,\psi_k)\, r(\rho,\theta_k,\psi_k)\, \hat{c}_k O_{k,m} \sum_{j=1}^{K} \frac{\partial}{\partial \rho}\Big(\alpha(\rho,\theta_j,\psi_j)\, r(\rho,\theta_j,\psi_j)\Big)\, \hat{c}_j O_{j,m}, \tag{49}$$

and

$$\left[\mathbf{I}^{\mathcal{C}}\right]_{2,2} \approx \frac{d^4 w^2}{\sigma^2} \sum_{m=1}^{M} \left(\sum_{k=1}^{K} \frac{\partial}{\partial \rho}\Big(\alpha(\rho,\theta_k,\psi_k)\, r(\rho,\theta_k,\psi_k)\Big) \hat{c}_k O_{k,m} \right)^2 . \tag{50}$$

The CRB matrix is, thus, $\left(\mathbf{I}^{\mathcal{C}}\right)^{-1}$ with diagonal entries:

$$\left[\mathbf{C}^{\mathcal{C}}\right]_{1,1} = \frac{1}{\left[\mathbf{I}^{\mathcal{C}}\right]_{1,1} - \frac{([\mathbf{I}^{\mathcal{C}}]_{1,2})^2}{[\mathbf{I}^{\mathcal{C}}]_{2,2}}} \geq \left(\left[\mathbf{I}^{\mathcal{C}}\right]_{1,1}\right)^{-1} \tag{51}$$

$$= \frac{\sigma^2}{d^4} \left(\sum_{m=1}^{M} \left(\sum_{k=1}^{K} \alpha(\rho,\theta_k,\psi_k)\, r(\rho,\theta_k,\psi_k)\, \hat{c}_k O_{k,m} \right)^2 \right)^{-1} \tag{52}$$

$$\geq \frac{\sigma^2}{d^4 \left(\sum_{k=1}^{K} \hat{c}_k\right)^2} \frac{1}{\sum_{m=1}^{M} \sum_{k=1}^{K} \Big(\alpha(\rho,\theta_k,\psi_k)\, r(\rho,\theta_k,\psi_k)\Big)^2 O_{k,m}^2}, \tag{53}$$

and

$$\left[\mathbf{C}^{\mathcal{C}}\right]_{2,2} = \frac{1}{\left[\mathbf{I}^{\mathcal{C}}\right]_{2,2} - \frac{([\mathbf{I}^{\mathcal{C}}]_{1,2})^2}{[\mathbf{I}^{\mathcal{C}}]_{1,1}}} \geq \left(\left[\mathbf{I}^{\mathcal{C}}\right]_{2,2}\right)^{-1} \tag{54}$$

$$= \frac{\sigma^2}{d^4 w^2} \left(\sum_{m=1}^{M} \left(\sum_{k=1}^{K} \frac{\partial}{\partial \rho}\Big(\alpha(\rho,\theta_k,\psi_k)\, r(\rho,\theta_k,\psi_k)\Big) \hat{c}_k O_{k,m} \right)^2 \right)^{-1} \tag{55}$$

$$\geq \frac{\sigma^2}{d^4 w^2 \left(\sum_{k=1}^{K} \hat{c}_k\right)^2} \frac{1}{\sum_{m=1}^{M} \sum_{k=1}^{K} \Big(\frac{\partial}{\partial \rho}\alpha(\rho,\theta_k,\psi_k)\, r(\rho,\theta_k,\psi_k)\Big)^2 O_{k,m}^2} \tag{56}$$

$$= \frac{\sigma^2}{d^4 w^2 \left(\sum_{k=1}^{K} \hat{c}_k\right)^2} \frac{1}{\sum_{k=1}^{K} \sum_{m=1}^{M} \Big(\frac{\partial}{\partial \rho}\alpha(\rho,\theta_k,\psi_k)\, r(\rho,\theta_k,\psi_k)\Big)^2 O_{k,m}^2}, \tag{57}$$

which can be interpreted as lower bounds for the posterior variance of the estimates for parameters w and ρ, respectively. The final inequalities, (53) and (57), follow from Cauchy-Schwarz inequality; while interchanging the summations follow because the sums are finite[3].

S2.4.2 CRB for Estimating a Single Range Per Surface Element

In principle, we could instead seek to estimate a range and updated radiosity for each surface element in the cluster. This effectively means estimating K radiosity weights and ranges, i.e. $\{(w_k, \rho_k)\}_{k=1}^{K}$, independently[4] from the camera observations $y_m = q_m + n_m$, with:

$$q_m = d^2 \sum_{k=1}^{K} w_k \alpha(\rho_k,\theta_k,\psi_k)\, r(\rho_k,\theta_k,\psi_k)\, \hat{c}_k O_{k,m}. \tag{58}$$

In a similar fashion to Section S2.4.1, one could compute the FIs for the K pairs of unknown parameters under this alternative model: i.e., a pair (w_k, ρ_k) for each member $k = 1, 2, \ldots, K$ of the cluster. Let

[3] Put differently, it does not matter in what order we sum the entries of a 2D array.
[4] Without incorporating any regularity or spatial correlation assumptions.

$\mathbf{I}^{\mathcal{S}} \in \mathbb{R}^{2K\times 2K}$ denote the resulting FI matrix and $\mathbf{C}^{\mathcal{S}} \in \mathbb{R}^{2K\times 2K}$ the resulting CRB matrix for this setting. Hence, the respective CRBs for the radiosity weight w_k and range ρ_k of k-th surface element are bounded below by,

$$\left[\mathbf{C}^{\mathcal{S}}\right]_{k,k} \geq \frac{\sigma^2}{d^4 \hat{c}_k^2} \frac{1}{\sum_{m=1}^{M} \left(\alpha(\rho_k,\theta_k,\psi_k)\, r(\rho_k,\theta_k,\psi_k)\, O_{k,m}\right)^2} \tag{59}$$

and

$$\left[\mathbf{C}^{\mathcal{S}}\right]_{K+k,K+k} \geq \frac{\sigma^2}{d^4} \frac{1}{\hat{c}_k^2 w_k^2 \sum_{m=1}^{M} \left(\frac{\partial}{\partial \rho_k}\alpha(\rho_k,\theta_k,\psi_k)\, r(\rho_k,\theta_k,\psi_k)\right)^2 \left(O_{k,m}\right)^2} \tag{60}$$

respectively.

S2.4.3 Comparing Per Cluster Range vs Per Elemental Surface Range CRBs

Let the true object range from the origin be $\bar{\rho}$, and the true radiosity weight be $\bar{w}$, then the CRB for $\bar{\rho}$ when estimating it as a single parameter for the entire cluster of surface elements forming the object is:

$$\begin{aligned}
\left[\mathbf{C}^{\mathcal{C}}\right]_{2,2} &\geq \frac{\sigma^2}{d^4 \bar{w}^2 \left(\sum_{k=1}^{K} \hat{c}_k\right)^2} \frac{1}{\sum_{k=1}^{K}\sum_{m=1}^{M} \left(\frac{\partial}{\partial \bar{\rho}}\alpha(\bar{\rho},\theta_k,\psi_k)\, r(\bar{\rho},\theta_k,\psi_k)\right)^2 O_{k,m}^2} &(61)\\
&\geq \frac{\sigma^2}{d^4 \bar{w}^2 \left(\sum_{k=1}^{K} \hat{c}_k\right)^2} \frac{1}{K \max_k \sum_{m=1}^{M} \left(\frac{\partial}{\partial \bar{\rho}}\alpha(\bar{\rho},\theta_k,\psi_k)\, r(\bar{\rho},\theta_k,\psi_k)\right)^2 O_{k,m}^2}, &(62)
\end{aligned}$$

which follows from (57) with $\bar{\rho}$ replacing ρ. While the CRB for each $\rho_k = \bar{\rho}$ (with $w_k = \bar{w}/K$) in the alternative approach of estimating K ranges when treating each surface element composing the object independently is,

$$\left[\mathbf{C}^{\mathcal{S}}\right]_{K+k,K+k} \geq \frac{\sigma^2}{d^4 \bar{w}^2 \hat{c}_k^2/K^2} \frac{1}{\sum_{m=1}^{M} \left(\frac{\partial}{\partial \bar{\rho}}\alpha(\bar{\rho},\theta_k,\psi_k)\, r(\bar{\rho},\theta_k,\psi_k)\right)^2 O_{k,m}^2}, \tag{63}$$

which follows from (60) after replacing ρ_k with $\bar{\rho}$. We note that the following hold: $\left(\sum_{k=1}^{K} \hat{c}_k\right)^2 \geq \frac{\hat{c}_k^2}{K^2}$ and $K \max_k \sum_{m=1}^{M} \left(\frac{\partial}{\partial \bar{\rho}}\alpha(\bar{\rho},\theta_k,\psi_k)\, r(\bar{\rho},\theta_k,\psi_k)\right)^2 O_{k,m}^2 \geq \sum_{m=1}^{M} \left(\frac{\partial}{\partial \bar{\rho}}\alpha(\bar{\rho},\theta_k,\psi_k)\, r(\bar{\rho},\theta_k,\psi_k)\right)^2 O_{k,m}^2$. These two facts together lead to the desired conclusion that,

$$\left[\mathbf{C}^{\mathcal{C}}\right]_{2,2} \leq \left[\mathbf{C}^{\mathcal{S}}\right]_{K+k,K+k}.$$

In reality, because of the extremely mild dependence of $\alpha(\bar{\rho},\theta_k,\psi_k)$ and $r(\bar{\rho},\theta_k,\psi_k)$ on θ_k and ψ_k over the camera FOV the bounds derived for $\left[\mathbf{C}^{\mathcal{C}}\right]_{2,2}$ and $\left[\mathbf{C}^{\mathcal{S}}\right]_{K+k,K+k}$ are tight, and this enables comparison between the bounds.

An equivalent conclusion also arises from comparing the corresponding FIs $\left[\mathbf{I}^{\mathcal{C}}\right]_{2,2}$ and $\left[\mathbf{I}^{\mathcal{S}}\right]_{K+k,K+k}$.

In Supplementary Figure 8 we show the CRB variation for a hidden scene object when its range is estimated as just a single cluster of surface elements with the same range value and when it is subdivided into an increasing number of clusters. A worsening of the CRB for the range estimates is observed as the number of subclusters increases. The average range estimate CRB obtained when attempting to recover a range for each of the 32^2 surface elements that constitute the object independently exceeds the range estimate CRB obtained when all surface elements are enforced to have the same fixed range by over nine orders of magnitude. In the latter case, only one range estimate is to be recovered, whereas in the former 32^2 ranges will be estimated. As anticipated, the problem of estimating the range of the object becomes increasingly ill-conditioned with the number of subclusters.

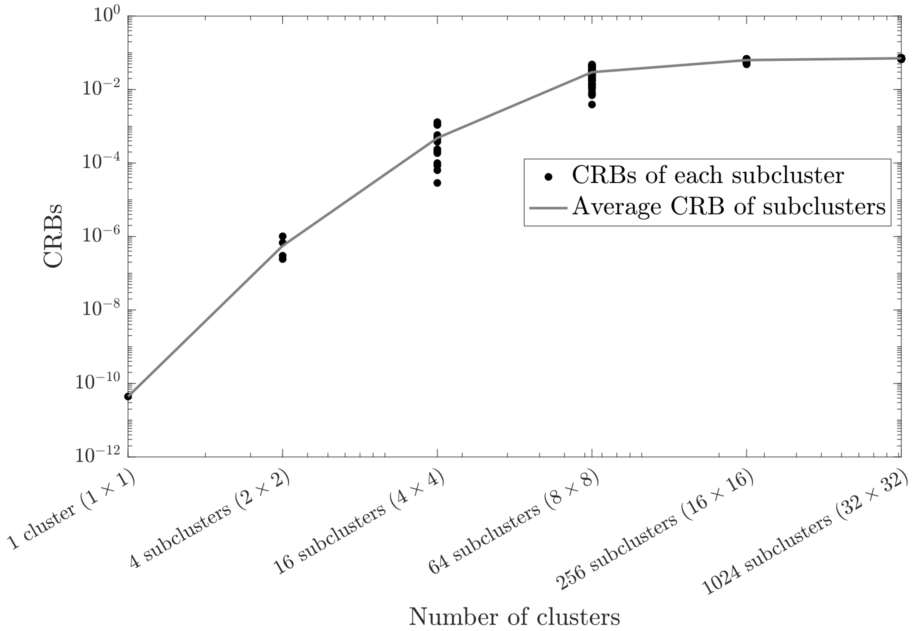

Supplementary Figure 8: Range estimation CRBs for a single object under varying levels of sub-discretization of the object. The hidden scene is a single object with fixed radiosity $\bar{c} = 1$, range $\bar{\rho} = 0.5$, and angular extents $\theta \in [{}^{11\pi}/_{60}, {}^{19\pi}/_{60}]$ and $\psi \in [{}^{4403\pi}/_{15000}, {}^{5707\pi}/_{15000}]$ in azimuth and elevation angles, respectively. Representing the hidden scene volume by 120×120 angular surface elements, the object is estimated as being composed of 32×32 contiguous surface elements. The variation of the computed range CRBs when the collection of surface elements constituting the object are combined into $k \times k$ subclusters, for $k = 1, 2, 4, 8, 16, 32$, and one range is to be estimated per subcluster. The ceiling plane is $h = 0.28$ m from above the horizontal edge. The number of measurements, i.e. pixels in the observation photograph, is $M = 126 \times 126$, the camera FOV is $\{[0, -0.46] \times [0, -0.46]\}$ m^2, and the measurement noise variance is $\sigma^2 = 1 \times 10^{-14}$ (roughly 45 dB).

Supplementary Note 3

S3 Fisher Information Orthogonality of the Projected-Elevation Spherical Coordinate System

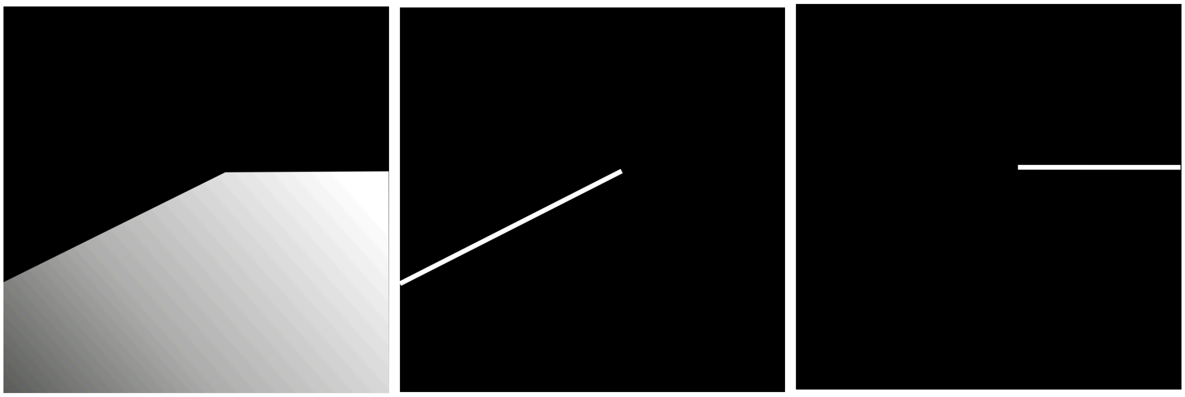

(a) Measurement i_m. **(b)** Azimuthal deriv. $\frac{\partial i_m}{\partial \theta}$. **(c)** Projected-elevation deriv. $\frac{\partial i_m}{\partial \psi}$.

Supplementary Figure 9: Measurement and depiction of derivatives of measurement with respect to the angular coordinates for an example hidden scene point. (a) Measurement contribution to the observation plane for an arbitrary hidden scene point located at (ρ, θ, ψ) with radiosity $c > 0$. (b) Derivative of the measurement contribution i_m with respect to the azimuthal angle parameter θ. (c) Derivative of the measurement contribution i_m with respect to the projected-elevation angle parameter ψ. The top-right corner is the origin of the measurement plane in all images, and the photographs are in the third quadrant in the xy-plane (i.e., the quadrant where $x, y < 0$).

S3.1 Proof of Fisher information orthogonality for an arbitrary hidden scene point

Consider a hidden scene point with radiosity c and location (ρ, θ, ψ) represented in the proposed projected-elevation spherical coordinate system. It was shown in Section S2 that the amount of light reaching the measurement plane patch $\mathcal{P}_m$ due to this hidden scene point is given by:

$$i_m(\rho, \theta, \psi) = \alpha_m c r_m \int_{\mathbf{p} \in \mathcal{P}_m} u\big(\theta - \tan^{-1}(p_x/p_y)\big)\, u\big(\psi - \tan^{-1}(h/p_y)\big)\, \mathrm{d}\mathbf{p}. \tag{64}$$

In addition, recall that its partial derivatives *wrt* the unknown parameters of the hidden scene point are:

$$\frac{\partial i_m(\rho, \theta, \psi)}{\partial c} = \frac{\alpha_m}{\|\mathbf{p}_m - \mathbf{s}^c\|_2^2} \int_{\mathbf{p} \in \mathcal{P}_m} u\big(\theta - \tan^{-1}(p_x/p_y)\big)\, u\big(\psi - \tan^{-1}(h/p_y)\big)\, \mathrm{d}\mathbf{p}, \tag{65}$$

$$\frac{\partial i_m(\rho, \theta, \psi)}{\partial \rho} = \alpha_m c \frac{\partial r_m}{\partial \rho} \int_{\mathbf{p} \in \mathcal{P}_m} u\big(\theta - \tan^{-1}(p_x/p_y)\big)\, u\big(\psi - \tan^{-1}(h/p_y)\big)\, \mathrm{d}\mathbf{p}, \tag{66}$$

where $\frac{\partial r_m}{\partial \rho} = -2r^2\left(\rho + \gamma\left(p_{m,y} + p_{m,x}\tan\theta + h\tan\psi\right)\right)$. Further, the desired derivatives are

$$\begin{aligned}
\frac{\partial i_m(\rho,\theta,\psi)}{\partial\theta} &= \alpha_m c r_m \int_{\mathbf{p}\in\mathcal{P}_m} \frac{\partial}{\partial\theta} u\big(\theta - \tan^{-1}(p_x/p_y)\big)\, u\big(\psi - \tan^{-1}(h/p_y)\big)\, \mathrm{d}\mathbf{p} \\
&\quad + \alpha_m c \frac{\partial r_m}{\partial\theta} \int_{\mathbf{p}\in\mathcal{P}_m} u\big(\theta - \tan^{-1}(p_x/p_y)\big)\, u\big(\psi - \tan^{-1}(h/p_y)\big)\, \mathrm{d}\mathbf{p} \\
&= \alpha_m c r_m \int_{\mathbf{p}\in\mathcal{P}_m} \delta\big(\theta - \tan^{-1}(p_x/p_y)\big)\, u\big(\psi - \tan^{-1}(h/p_y)\big)\, \mathrm{d}\mathbf{p} \\
&\quad + \alpha_m c \frac{\partial r_m}{\partial\theta} \int_{\mathbf{p}\in\mathcal{P}_m} u\big(\theta - \tan^{-1}(p_x/p_y)\big)\, u\big(\psi - \tan^{-1}(h/p_y)\big)\, \mathrm{d}\mathbf{p},
\end{aligned} \tag{67}$$

where $\frac{\partial r_m}{\partial\theta} = -2\gamma\rho r^2\left(p_{m,x}\sec^2\theta - \gamma^2(p_{m,y} + p_{m,x}\tan\theta + h\tan\psi)\tan\theta\sec^2\theta\right)$, and

$$\begin{aligned}
\frac{\partial i_m(\rho,\theta,\psi)}{\partial\psi} &= \alpha_m c r_m \int_{\mathbf{p}\in\mathcal{P}_m} u\left(\theta - \tan^{-1}(p_x/p_y)\right) \frac{\partial}{\partial\psi} u\left(\psi - \tan^{-1}(h/p_y)\right) \mathrm{d}\mathbf{p} \\
&\quad + \alpha_m c \frac{\partial r_m}{\partial\psi} \int_{\mathbf{p}\in\mathcal{P}_m} u\left(\theta - \tan^{-1}(p_x/p_y)\right) u\left(\psi - \tan^{-1}(h/p_y)\right) \mathrm{d}\mathbf{p} \\
&= \alpha_m c r_m \int_{\mathbf{p}\in\mathcal{P}_m} u\left(\theta - \tan^{-1}(p_x/p_y)\right) \delta\left(\psi - \tan^{-1}(h/p_y)\right) \mathrm{d}\mathbf{p} \\
&\quad + \alpha_m c \frac{\partial r_m}{\partial\psi} \int_{\mathbf{p}\in\mathcal{P}_m} u\left(\theta - \tan^{-1}(p_x/p_y)\right) u\left(\psi - \tan^{-1}(h/p_y)\right) \mathrm{d}\mathbf{p},
\end{aligned} \tag{68}$$

where $\frac{\partial r}{\partial\psi} = -2\gamma\rho r^2\left(h\sec^2\psi - \gamma^2(p_{m,y} + p_{m,x}\tan\theta + h\tan\psi)\tan\psi\sec^2\psi\right)$. Assuming the dependence of r_m and α_m on the parameters ρ, θ and ψ to be very mild, then it follows that:

$$\frac{\partial i_m}{\partial\rho} \approx 0, \tag{69}$$

$$\begin{aligned}
\frac{\partial i_m}{\partial\theta} &\approx \alpha_m c r_m \int_{\mathbf{p}\in\mathcal{P}_m} \delta\big(\theta - \tan^{-1}(p_x/p_y)\big)\, u\big(\psi - \tan^{-1}(h/p_y)\big)\, \mathrm{d}\mathbf{p} \\
&= \begin{cases} \eta_m, & \text{for } \mathcal{P}_m \bigcap \{(p_{m,y}\cot\theta, p_{m,y}) : p_{m,y} \le -h\cot\psi\} \\ 0, & \text{otherwise} \end{cases}
\end{aligned} \tag{70}$$

and,

$$\begin{aligned}
\frac{\partial i_m}{\partial\psi} &\approx \alpha_m c r_m \int_{\mathbf{p}\in\mathcal{P}_m} u\left(\theta - \tan^{-1}(p_x/p_y)\right) \delta\left(\psi - \tan^{-1}(h/p_y)\right) \mathrm{d}\mathbf{p} \\
&= \begin{cases} \bar{\eta}_m, & \text{for } \mathcal{P}_m \bigcap \{(p_{m,x}, -h\cot\psi) : p_{m,x} \ge -h\tan\theta\cot\psi\} \\ 0, & \text{otherwise} \end{cases},
\end{aligned} \tag{71}$$

where $\eta_m \neq 0$ and $\bar{\eta}_m \neq 0$ are strictly non-zero quantities denoting the values of the pixels that intersect the lines shown in Supplementary Figures 9(b) and 9(c). Because of (69),

$$\sum_{m=1}^{M} \frac{\partial i_m}{\partial\theta}\frac{\partial i_m}{\partial\rho} = \sum_{m=1}^{M} \frac{\partial i_m}{\partial\rho}\frac{\partial i_m}{\partial\theta} \approx 0,$$

and

$$\sum_{m=1}^{M} \frac{\partial i_m}{\partial\psi}\frac{\partial i_m}{\partial\rho} = \sum_{m=1}^{M} \frac{\partial i_m}{\partial\rho}\frac{\partial i_m}{\partial\psi} \approx 0,$$

trivially. This fact also makes $\frac{\partial i_m}{\partial\rho}\frac{\partial i_m}{\partial c}$ small (see Supplementary Figure 10(a), entries (c,ρ) and (ρ,c)). However, $\sum_m \frac{\partial i_m}{\partial\rho}\frac{\partial i_m}{\partial c}$ is roughly the same order of magnitude as $\sum_m\left(\frac{\partial i_m}{\partial\rho}\right)^2$ and $\sum_m\left(\frac{\partial i_m}{\partial c}\right)^2$.

Furthermore, the measurement pixel patches that intersect the line where the azimuthal derivative is non-zero (Supplementary Figure 9(b)) do not coincide with those measurement pixel patches that intersect the line where the projected-elevation derivative is non-zero (Supplementary Figure 9(c)), apart from the single pixel that contains the point $(-h \tan\theta \cot\psi, -h\tan\theta)$. This means that:

$$\frac{\partial i_m}{\partial \theta}\frac{\partial i_m}{\partial \psi} = 0$$

for all $m = 1, \ldots, M$ apart from the single pixel patch $\mathcal{P}_m$ with $(-h\tan\theta\cot\psi, -h\tan\theta) \in \mathcal{P}_m$ (i.e., includes the meeting point of the two lines in Supplementary Figures 9(b) and 9(c)). Thus,

$$\sum_{m=1}^{M} \frac{\partial i_m}{\partial \theta}\frac{\partial i_m}{\partial \psi} = \sum_{m=1}^{M} \frac{\partial i_m}{\partial \psi}\frac{\partial i_m}{\partial \theta} \approx 0.$$

Combining these results show that all off-diagonal terms will be approximately zero. The FI matrix for an example hidden scene configuration is presented in the subsequent section.

S3.2 Example I: Fisher information matrices for a hidden scene point

The FI matrix computed for an example hidden scene point for a 125×125-pixel resolution measurement photograph, with an observation photograph FOV of size 0.5 m-by-0.5 m and a measurement SNR of 75 dB is shown in Supplementary Figure 10. The hidden room is 1 m in width and length, while its height is 1.2 m, making the height h of the doorway head 0.2 m.

Supplementary Figure 10(a) demonstrates that when the hidden scene point is represented in the projected-elevation spherical coordinate system, the off-diagonal terms are approximately zero, making the FI matrix approximately diagonal for the example scenario considered. The FI matrix is not precisely diagonal due to the effect of radial falloff and the Lambertian BRDF factors. However, for the same example configuration, using conventional spherical (Supplementary Figure 10(b)) and Cartesian (Supplementary Figure 10(c)) coordinates as hidden scene point representations produce "highly non-diagonal" FI matrices correspondingly. In particular, the off-diagonal term for (θ, ϕ)-pair is sizeable – roughly a few orders of magnitude larger than the off-diagonal term for (θ, ψ)-pair.

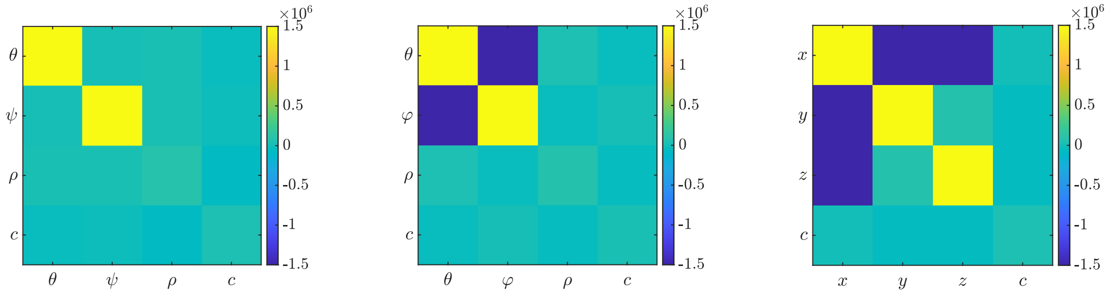

(a) Projected-elevation spherical. **(b)** Spherical. **(c)** Cartesian.

Supplementary Figure 10: Fisher information matrices in various coordinate systems for estimating a hidden point target. The 4-by-4 FI matrices computed for estimating all four unknown parameters of the hidden target are shown for various coordinate systems. The off-diagonal terms of the matrix are approximately zero in (a) indicating that the unknown positional parameters (ρ, θ, ψ) are roughly information orthogonal in projected-elevation spherical coordinates. In (b) and (c), the off-diagonals are not generally zero, thus the target's unknown parameters are not information orthogonal when represented in spherical and Cartesian coordinates. Here, the point target has unit radiosity and is located at $(x, y, z) = (1/2, 1/2, 1/2)$ in Cartesian coordinates, i.e. $(\rho, \theta, \psi) = (\sqrt{3}/2, \pi/4, \pi/4)$ in projected-elevation spherical or $(\rho, \theta, \varphi) = (\sqrt{3}/2, \pi/4, \cos^{-1} 1/\sqrt{3})$ in standard spherical coordinates.

S3.3 Example II: CRB matrices for a hidden scene point

The CRB matrix for the same example hidden scene point (as above) for a 125×125-pixel resolution measurement photograph, with an observation photograph FOV of size 0.5 m-by-0.5 m and a measurement SNR of 75 dB is shown in Supplementary Figure 11. The hidden room is 1 m in width and length, while its height is 1.2 m, making the height h of the doorway head 0.2 m.

Supplementary Figure 11(a) demonstrates that the CRBs are almost zero for estimating the angular parameters for the hidden scene point, and the correlations between those angular parameters estimates—as well as between them and the range and radiosity parameters estimates (indicated by the off-diagonal terms)—are very small. Strong correlations will exist between the range and radiosity parameter estimates, however, in addition to having high CRBs themselves when using the projected-elevation spherical coordinates representation.

Using spherical coordinate representation, as indicated by the plot in Supplementary Figure 11(b), the CRBs for estimates of the range ρ and radiosity c parameters are high. There is also a high correlation between the elevation angle and radiosity parameter estimates. Moreover, as shown in Supplementary Figure 11(c), using a Cartesian coordinate representation yields fairly even CRBs for the x, y, and z positional parameter estimates, as well as pairwise correlations among them. Estimates of the radiosity parameter c will still have high a CRB like the other two cases representations.

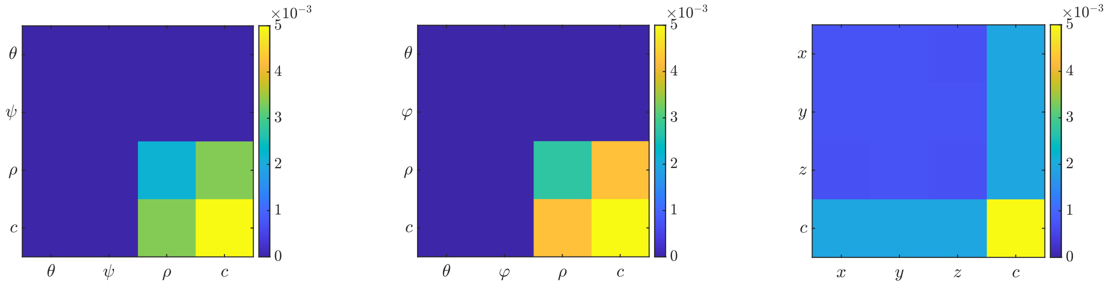

(a) Projected-elevation spherical. **(b)** Spherical. **(c)** Cartesian.

Supplementary Figure 11: Crámer-Rao bounds in various coordinate systems for estimating a hidden point target. Shown here are the corresponding inverses of the FI matrices given in Supplementary Figure 10 which demonstrate the effectiveness of the projected-elevation spherical coordinate system at making the positional parameters uncorrelated. The projected-elevation spherical coordinate CRB matrix shown in (a) suggests that the correlations between each pair (θ, ψ), (θ, ρ), (θ, c), (ψ, ρ) and (ψ, c) is zero; this is not true in general for the other coordinate system representations shown in (b) and (c).

Supplementary Note 4

S4 Inversion method: Proposed TERI reconstruction algorithm

The proposed two-edge resolved imaging approach presented in the main manuscript aims to reconstruct the hidden scene in 3D by solving the following optimization problem (main manuscript equation (4)):

$$\arg \min_{(\mathbf{f},\boldsymbol{\rho},\mathbf{b})\geq\mathbf{0}} \|\mathbf{y} - \mathbf{A}(\boldsymbol{\rho})\mathbf{f} - \mathbf{B}\mathbf{b}\|_2^2 + \mathcal{R}(\mathbf{f}). \tag{72}$$

The exact reconstruction TERI algorithm (summarized in Figure 2 of the main manuscript) proceeds as follows:

STEP I: Shape reconstruction

1. Initialize regularization parameter $\lambda_1 > 0$.
2. Initialize integer $J_{\max} > 0$ for the maximum number of clusters to discover.
3. Initialize $\boldsymbol{\rho} \in \mathbb{R}^{N_\theta N_\psi}$ to be a vector of constant positive entries equal to $\rho_0 > 0$ (i.e., $\boldsymbol{\rho} = \boldsymbol{\rho}_0 = \rho_0\mathbf{1}$).
4. For each colour channel, the optimization $(\widehat{\mathbf{f}}, \widehat{\mathbf{b}}) = \arg\min_{\mathbf{f}\geq 0,\mathbf{b}\geq 0} \|\mathbf{y} - \mathbf{A}(\boldsymbol{\rho}_0)\mathbf{f} - \mathbf{B}\mathbf{b}\|_2^2 + \lambda_1 \|\mathbf{f}\|_1$ is solved using ADMM [10] to recover $\widehat{\mathbf{f}}$ per color channel. (That is, using $\mathbf{y} = \mathbf{y}_{\mathrm{R}}$ the red channel measurement produces a reconstruction for the red channel of the hidden scene $\widehat{\mathbf{f}}_{\mathrm{R}}$, and similarly for the G- and B-channels.)
5. Given the reconstructions $\widehat{\mathbf{f}}_{\mathrm{R}}$, $\widehat{\mathbf{f}}_{\mathrm{G}}$ and $\widehat{\mathbf{f}}_{\mathrm{B}}$, compute the sum $\mathbf{f}_{\mathrm{grey}} = \widehat{\mathbf{f}}_{\mathrm{R}} + \widehat{\mathbf{f}}_{\mathrm{G}} + \widehat{\mathbf{f}}_{\mathrm{B}}$.
6. Reshape $\mathbf{f}_{\mathrm{grey}}$ into an $N_\theta \times N_\phi$ image and combine contiguous surface elements (i.e., surface elements that share an edge) into the same cluster. Thus, the computed clusters $\{\mathcal{C}_j\}_{j=1}^{J}$ will be angularly separated (i.e., they will not be neighbours).
7. If $J > J_{\max}$ compute the overall radiosity for each cluster, by summing the estimated radiosities of the surface elements constituting the cluster. Keep the $J_{\max} - 1$ clusters with the highest total radiosities and combine the remaining clusters into one larger cluster.

STEP II: Range reconstruction

8. Compute $\mathbf{y}_{\mathrm{grey}} = \widehat{\mathbf{y}}_{\mathrm{R}} + \widehat{\mathbf{y}}_{\mathrm{G}} + \widehat{\mathbf{y}}_{\mathrm{B}}$.
9. Solve the optimization $(\widehat{\boldsymbol{\rho}}^{\mathcal{C}}, \widehat{\mathbf{w}}, \widehat{\mathbf{b}}) = \arg\min_{(\boldsymbol{\rho}^{\mathcal{C}},\mathbf{w},\mathbf{b})\geq 0} \left\|\mathbf{y}_{\mathrm{grey}} - \mathbf{D}(\boldsymbol{\rho}^{\mathcal{C}})\mathbf{w} - \mathbf{B}\mathbf{b}\right\|_2^2$ using an accelerated projected gradient algorithm [11], to reconstruct a range for each of the J (or $J_{\max}$) clusters (recovered in line 6 above). Details of this algorithm are in the subsequent section (and summarized in Algorithm 1).
10. For each colour channel the optimization $(\widehat{\mathbf{f}}^{\mathrm{TV}}, \widehat{\mathbf{b}}^{\mathrm{TV}}) = \arg\min_{\mathbf{f}\geq 0,\mathbf{b}\geq 0} \left\|\mathbf{y} - \mathbf{A}(\widehat{\boldsymbol{\rho}}^{\mathcal{C}})\mathbf{f} - \mathbf{B}\mathbf{b}\right\|_2^2 + \lambda_{\mathrm{TV}} \|\mathbf{f}\|_{\mathrm{TV}}$ is solved using FISTA [11] to obtain a refined radiosity estimate for the hidden scene clusters identified in STEP I. (That is, using the red channel measurement $\mathbf{y} = \mathbf{y}_{\mathrm{R}}$ produces a reconstruction for the red channel of the hidden scene $\widehat{\mathbf{f}}_{\mathrm{R}}^{\mathrm{TV}}$, and similarly for the G- and B-channels.)
11. Combine the range estimates $\widehat{\boldsymbol{\rho}}^{\mathcal{C}}$ and the refined radiosities $\widehat{\mathbf{f}}^{\mathrm{TV}}$ to produce a full-colour 3D visualization of the hidden scene.

S4.1 Accelerated project gradient algorithm for range reconstruction

When $\mathbf{f}$ is fixed in (72), for instance by using an initial estimate obtained from **Step I** of the reconstruction algorithm, we obtain the non-linear inverse problem,

$$\mathbf{y}_{\text{grey}} = \mathbf{D}(\boldsymbol{\rho}^{\mathcal{C}})\mathbf{w} + \mathbf{B}\mathbf{b} + \mathbf{n} \tag{73}$$

for estimating the unknown range vector $\boldsymbol{\rho}^{\mathcal{C}}$, whose elements correspond to the unknown ranges for each of the J clusters, as well as their corresponding radiosity weights $\mathbf{w}$.

To recover $\boldsymbol{\rho}^{\mathcal{C}}$ and $\mathbf{w}$, we solve a non-negative least squares problem:

$$\arg \min_{(\boldsymbol{\rho}^{\mathcal{C}}, \mathbf{w}, \mathbf{b}) \geq 0} \left\| \mathbf{y}_{\text{grey}} - \mathbf{D}(\boldsymbol{\rho}^{\mathcal{C}})\mathbf{w} - \mathbf{B}\mathbf{b} \right\|_2^2 ,$$

using the fast iterative shrinkage thresholding algorithm [11] with a fixed step size.

Algorithm 1 Proximal gradient algorithm for range reconstruction

---

Input: Data $\mathbf{y}_{\text{grey}}$, step sizes $\eta_{\text{range-step}}, \eta_{\text{rad-step}}, \eta_{\text{bg-step}} > 0$
Initialize:
$\boldsymbol{\rho}_0^{\mathcal{C}} > 0$
$\mathbf{w}_0 > 0$
$\mathbf{b}_0 > 0$
$\mathbf{u}_1 = \boldsymbol{\rho}_0^{\mathcal{C}}$, $\widetilde{\mathbf{w}}_1 = \mathbf{w}_0$, $\widetilde{\mathbf{b}}_1 = \mathbf{b}_0$ and $t_1 = 1$
for $k = 1, 2, \ldots,$ `Max_iteration` **do**
$\quad \boldsymbol{\rho}_k^{\mathcal{C}} = \max\left(\mathbf{u}_k - \eta_{\text{range-step}} \nabla_{\boldsymbol{\rho}} \mathbf{D}(\boldsymbol{\rho}_{k-1}^{\mathcal{C}}) \left(\mathbf{D}(\boldsymbol{\rho}_{k-1}^{\mathcal{C}})\mathbf{w}_{k-1} + \mathbf{B}\mathbf{b}_{k-1} - \mathbf{y}_{\text{grey}}\right), 0\right)$
$\quad \mathbf{w}_k = \max\left(\widetilde{\mathbf{w}}_k - \eta_{\text{rad-step}} \mathbf{D}(\boldsymbol{\rho}_k^{\mathcal{C}})^\top \left(\mathbf{D}(\boldsymbol{\rho}_k^{\mathcal{C}})\mathbf{w}_{k-1} + \mathbf{B}\mathbf{b}_{k-1} - \mathbf{y}_{\text{grey}}\right), 0\right)$
$\quad \mathbf{b}_k = \max\left(\widetilde{\mathbf{b}}_k - \eta_{\text{bg-step}} \mathbf{B}^\top \left(\mathbf{D}(\boldsymbol{\rho}_k^{\mathcal{C}})\mathbf{w}_k + \mathbf{B}\mathbf{b}_{k-1} - \mathbf{y}_{\text{grey}}\right), 0\right)$
$\quad t_{k+1} = \frac{1+\sqrt{4t_k^2+1}}{2}$
$\quad \mathbf{u}_{k+1} = \boldsymbol{\rho}_k^{\mathcal{C}} + \left(\frac{t_k - 1}{t_{k+1}}\right) \left(\boldsymbol{\rho}_k^{\mathcal{C}} - \boldsymbol{\rho}_{k-1}^{\mathcal{C}}\right)$
$\quad \widetilde{\mathbf{w}}_{k+1} = \mathbf{w}_k + \left(\frac{t_k - 1}{t_{k+1}}\right) \left(\mathbf{w}_k - \mathbf{w}_{k-1}\right)$
$\quad \widetilde{\mathbf{b}}_{k+1} = \mathbf{b}_k + \left(\frac{t_k - 1}{t_{k+1}}\right) \left(\mathbf{b}_k - \mathbf{b}_{k-1}\right)$
end for
$\widehat{\boldsymbol{\rho}}^{\mathcal{C}} \leftarrow \boldsymbol{\rho}_k^{\mathcal{C}}$
$\widehat{\mathbf{w}} \leftarrow \mathbf{w}_k$
Output: $\left(\widehat{\boldsymbol{\rho}}^{\mathcal{C}}, \widehat{\mathbf{w}}\right)$

S4.2 Variant of TERI algorithm I: No total variation refinement

The total variation (TV) prior introduced in the penultimate stage of STEP II promotes piecewise smoothness in the computed hidden scene reconstruction. Consequently, it produces refined radiosity estimates for surface elements in each reconstructed hidden scene cluster. Although this refinement produces more visually accurate results—as seen in Figures 3 and 4 of the main manuscript and in Supplementary Figure 33(e)—compared to combining the reconstructed ranges with the initial shape estimates computed in Step I of TERI, computational complexity increases. This simpler variant of the TERI algorithm is obtained by bypassing the TV-based radiosity update. Instead, radiosities estimated in STEP I and the range estimates computed by solving the NLIP are used to produce a 3D visualization of the hidden scene. A summary of this simpler variant of the reconstruction algorithm is outlined in Supplementary Figure 12.

Henceforth, this lower complexity variant of the proposed TERI algorithm will be used to produce all reconstructions presented in this supplementary document, unless otherwise stated.

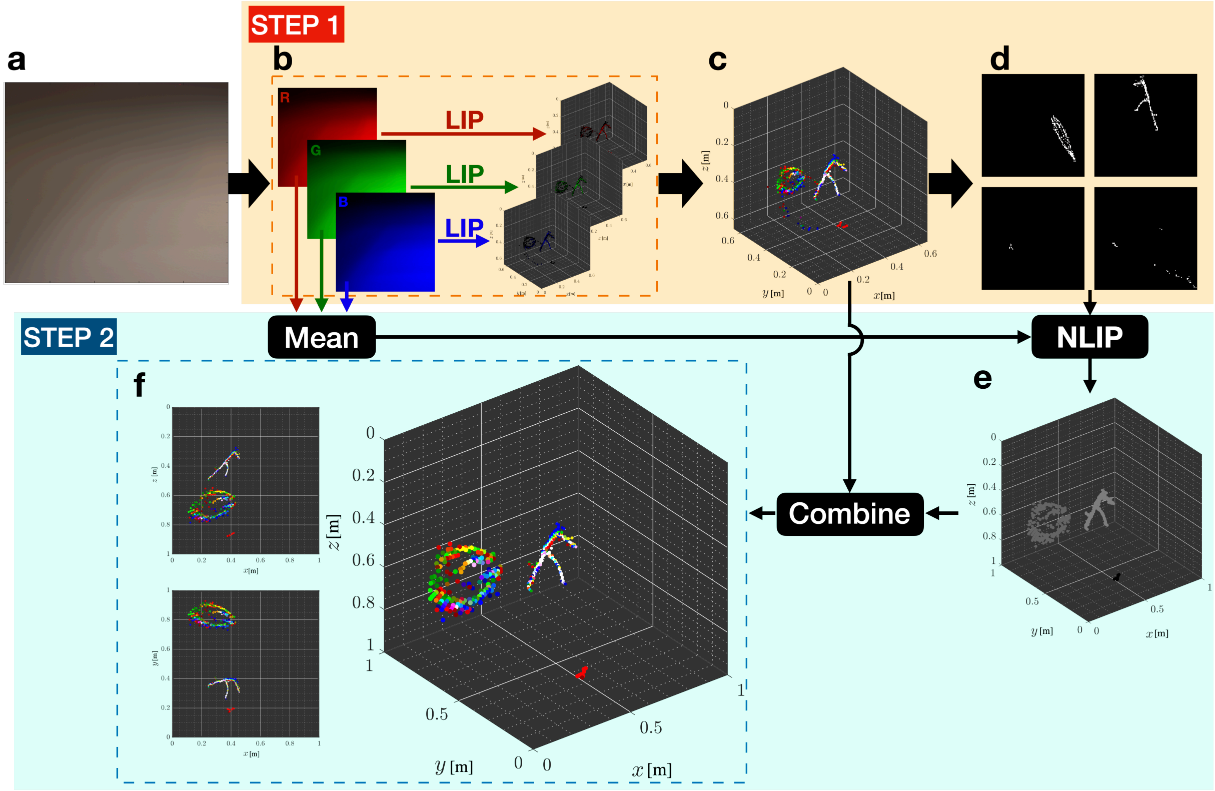

Supplementary Figure 12: Two-step reconstruction procedure. (a) Measured penumbra photograph. (b) A linear inverse problem (LIP) is solved, per colour channel, to recover the azimuth and projected-elevation representation (i.e., the shapes) of hidden scene objects, with hidden scene assumed to be confined to a single fixed range. (c) Colour visualisation of the initial angular reconstruction. (d) The reconstruction is analysed for connected surface elements which most likely belong to the same cluster. Four such clusters were identified, one for each of the three objects and a fourth (bottom right) for spurious elements. (e) A non-linear inverse problem (NLIP) is solved to estimate four ranges and four global radiosities, one for each cluster identified in d. (f) Multiple views of the 3D colour reconstruction produced by inpainting the estimated ranges onto the recovered shapes in c; a 3D view (second column), plan view (first column, top), and a side view (first column, bottom). (Best viewed in colour.)

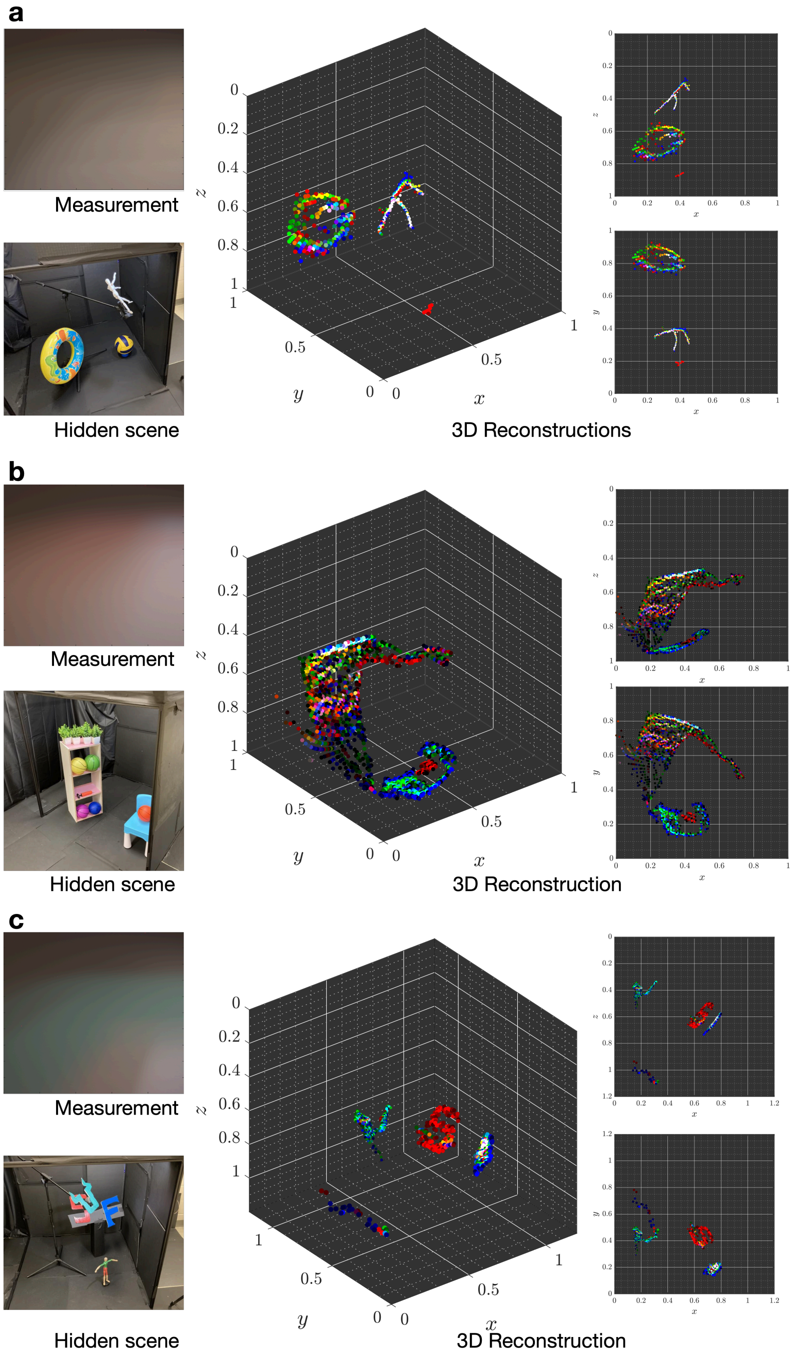

Supplementary Figure 13: Colour 3D reconstructions of three hidden scenes with simplified variant of TERI. (a) Play scene configuration. (b) Work scene configuration. (c) USF scene configuration. For each configuration, the first column shows the NLOS measurement photograph (top), and a line-of-sight photograph of the true scene (bottom); the second column shows the 3D full-colour reconstruction; and the last column shows the side (top) and plan (bottom) views of the 3D reconstruction.

Supplementary Note 5

S5 Additional Reconstructions

S5.1 Reconstructions from Single Snapshots

To improve measurement SNR, fifty independent 7331 ms-exposure snapshots were combined to make a single photograph using median filtering, and the resulting increased-SNR measurements were processed using the proposed approach to produce the reconstructions reported in the main manuscript (Figures 2, 3, 4, and Table 1). Presented in Supplementary Figures 14, 15 and 19 are corresponding reconstructions for the same scenes from their corresponding single exposure snapshots (i.e., without combining multiple exposures). The obtained results demonstrate that the reconstruction performance is equally compelling, with minimal to no degradation in the reconstruction fidelity even when a single exposure snapshot is used.

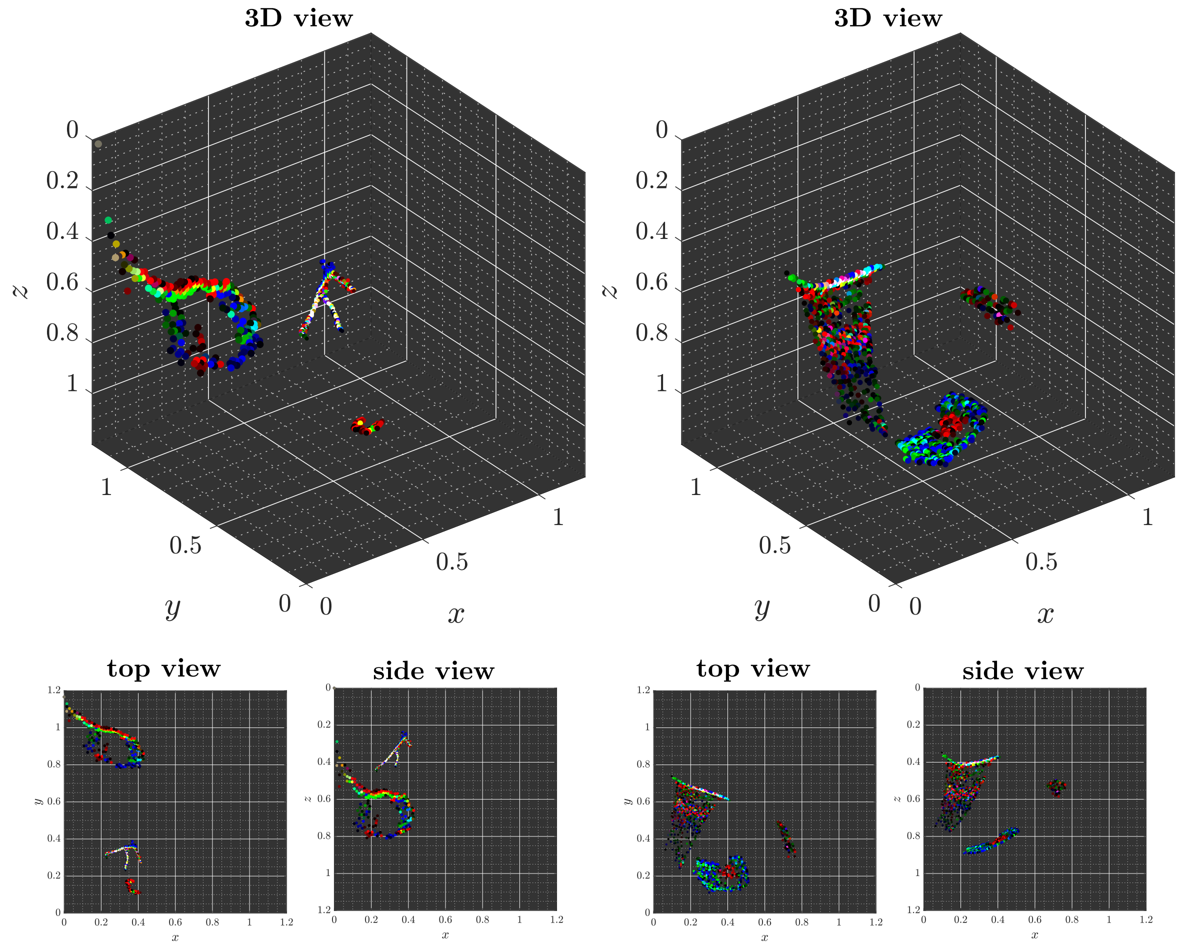

(a) Play scene. **(b)** Work scene.

Supplementary Figure 14: Single exposure snapshot reconstructions. Reconstructions obtained from a single exposure snapshot for the same scenes reported in the main manuscript: (a) Play scene, (b) Work scene. Here, however, no averaging of multiple snapshots was performed to improve the measurement photograph's SNR.

Supplementary Table 1: Experimentally measured and estimated ranges of hidden scene objects shown in Supplementary Figures 14 and 15. In contrast to the main manuscript Table 1, each reconstruction is obtained from one single exposure snapshot. The average range reconstruction error is roughly 12.3 cm demonstrating surprisingly accurate range estimation. Excluding the two-mannequins scene, the average error in the range estimate is 11.8 cm, which is only slightly worse than the average error computed in Table 1 of the main manuscript.

**Scene**	**Object**	**Measured range [m]**	**Estimated range [m]**
Play scene	Doughnut	1.32	1.17
	Mannequin	0.58	0.59
	Volleyball	1.14	0.87
Work scene	Shelf	1.12	0.82
	Basketball	0.99	0.94
	Chair	1.02	0.94
USF scene	U	0.64	0.69
	S	1.12	1.03
	F	1.07	1.03
	Mannequin	1.09	1.23
Two Mannequins scene	T-pose	0.86	0.64
	Marching-pose	1.04	0.97

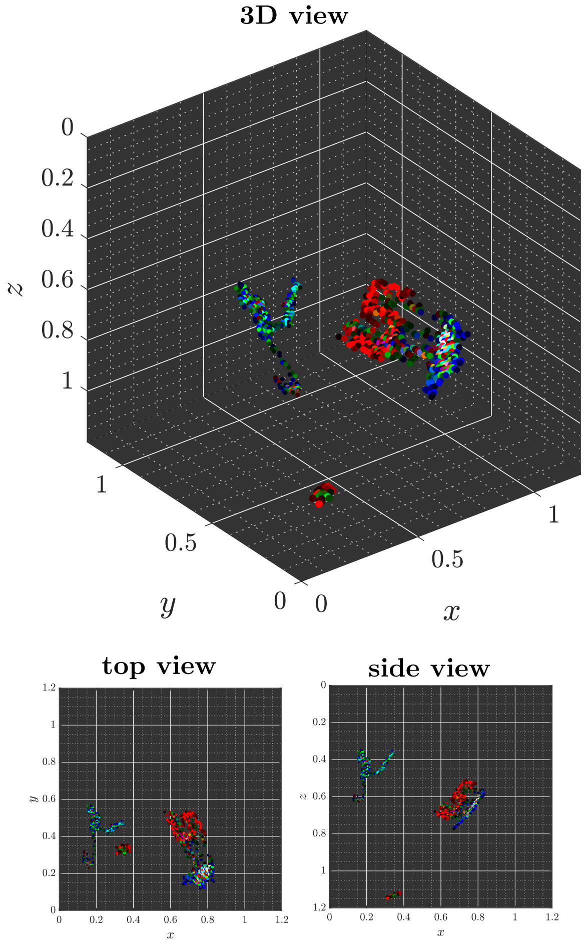

(a) USF scene.

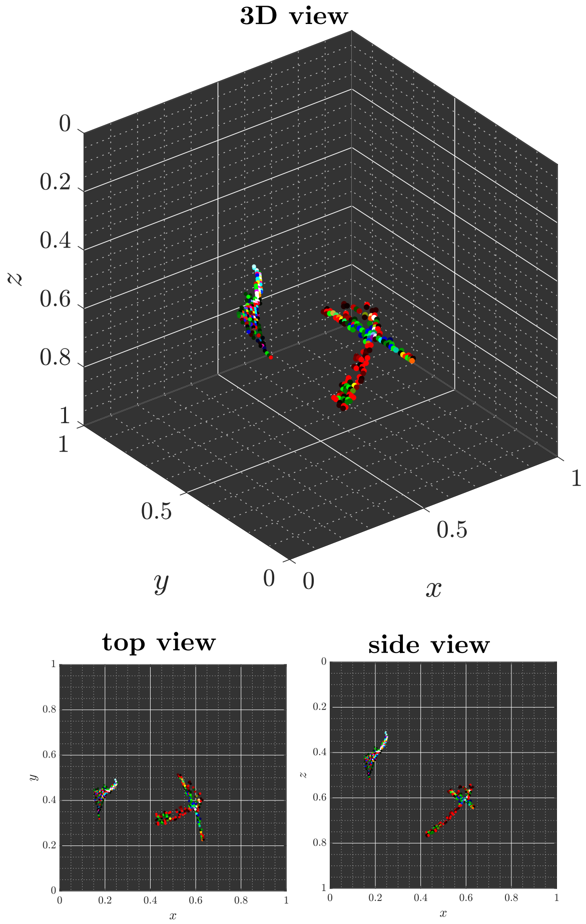

(b) Two Mannequins scene (no ambient light).

Supplementary Figure 15: Single exposure snapshot reconstructions. Reconstructions obtained from a single exposure snapshot for the same scenes reported in the main manuscript: (a) USF scene, (b) Two mannequins scene with no additional ambient illumination introduced. Here, however, no averaging of multiple snapshots was performed to improve the measurement photograph's SNR.

S5.2 Reconstructions in Visible Side Ambient Light: Experimental details and additional experiment

Without an ability to control the illumination of the hidden scene, passive NLOS imaging methods are inherently less robust to visible side ambient light contributions compared to active NLOS imaging methods. Here, the robustness of the proposed approach to increasing amounts of visible side ambient illumination is investigated. Supplementary Figure 16 shows the experimental setup used to introduce additional visible side illumination for the results reported in Figure 4 of the main manuscript, and a failed reconstruction obtained under an excessive amount of ambient illumination is shown in Supplementary Figure 18(e).

The experimental conditions were achieved by illuminating the visible scene area with the flashlight of a smartphone with controllable brightness levels. Four levels (i.e., none, low, medium, and high) of additional visible scene ambient illumination brightness were tested. In Supplementary Figure 18, the three reconstructions (i.e., none, low, and medium levels) are duplicated from the main manuscript and shown alongside the fourth ambient light level (i.e., high level) for ease of comparison.

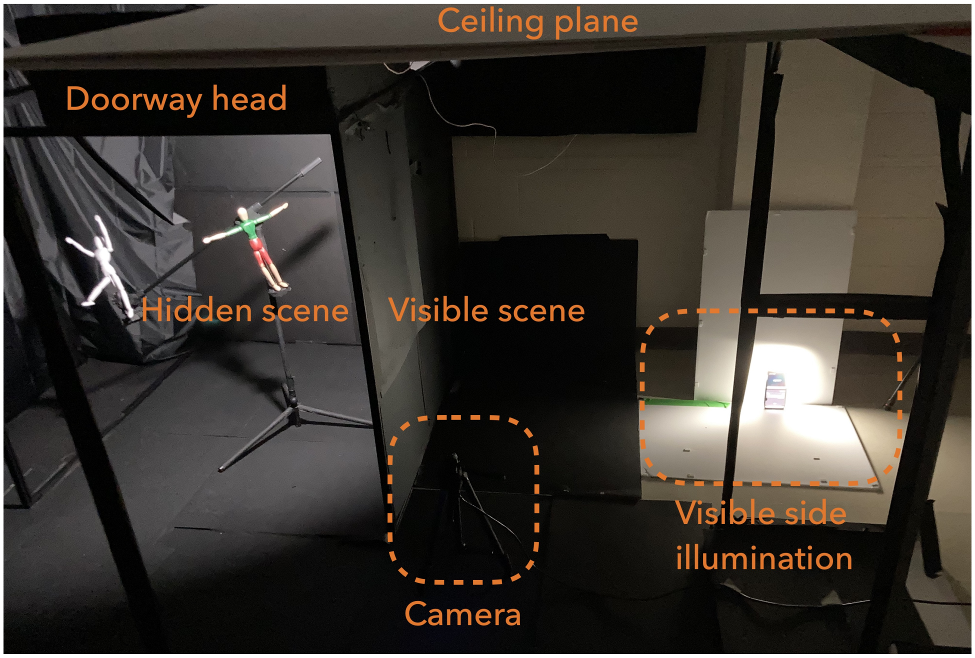

Supplementary Figure 16: A labelled photograph of the experimental setup with visible side illumination. The source of the visible side light is a smartphone flashlight directly illuminating two white foam boards and the white walls and floor of the visible scene. The light rays reflected by these surfaces diffusely illuminate the visible ceiling plane.

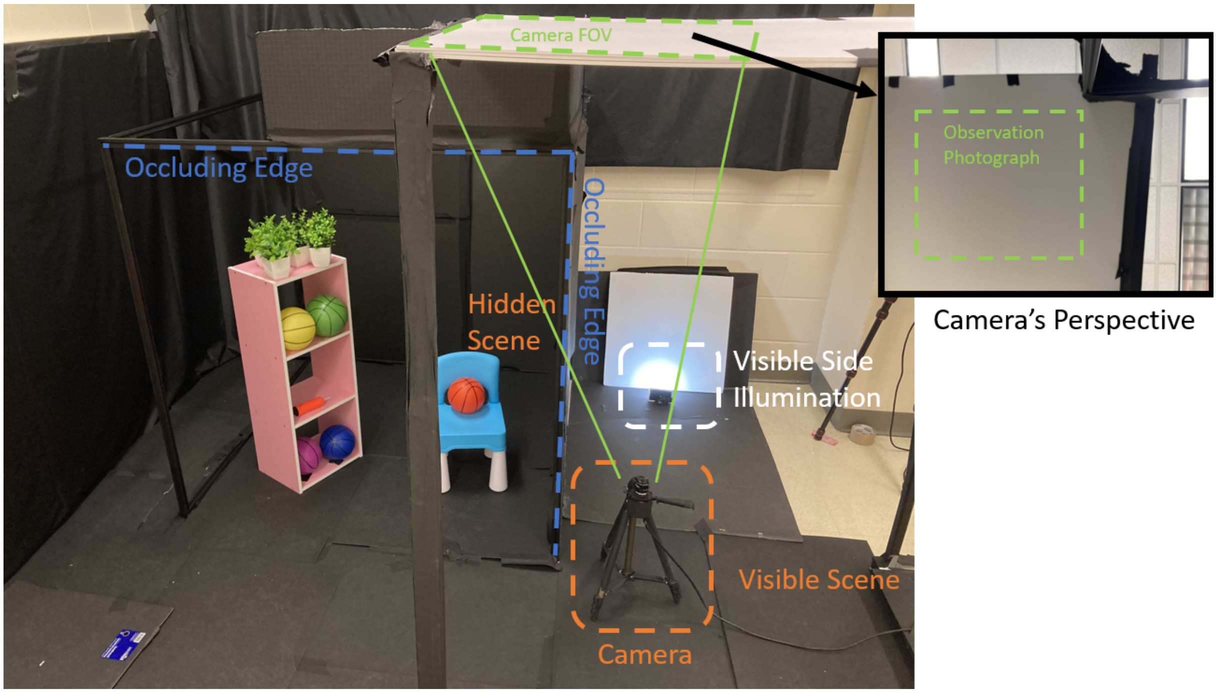

Supplementary Figure 17: Additional labelled photograph of the experimental setup. Here, the inset shows the view from the camera's perspective, which is of a white foamboard that plays the role of the ceiling.

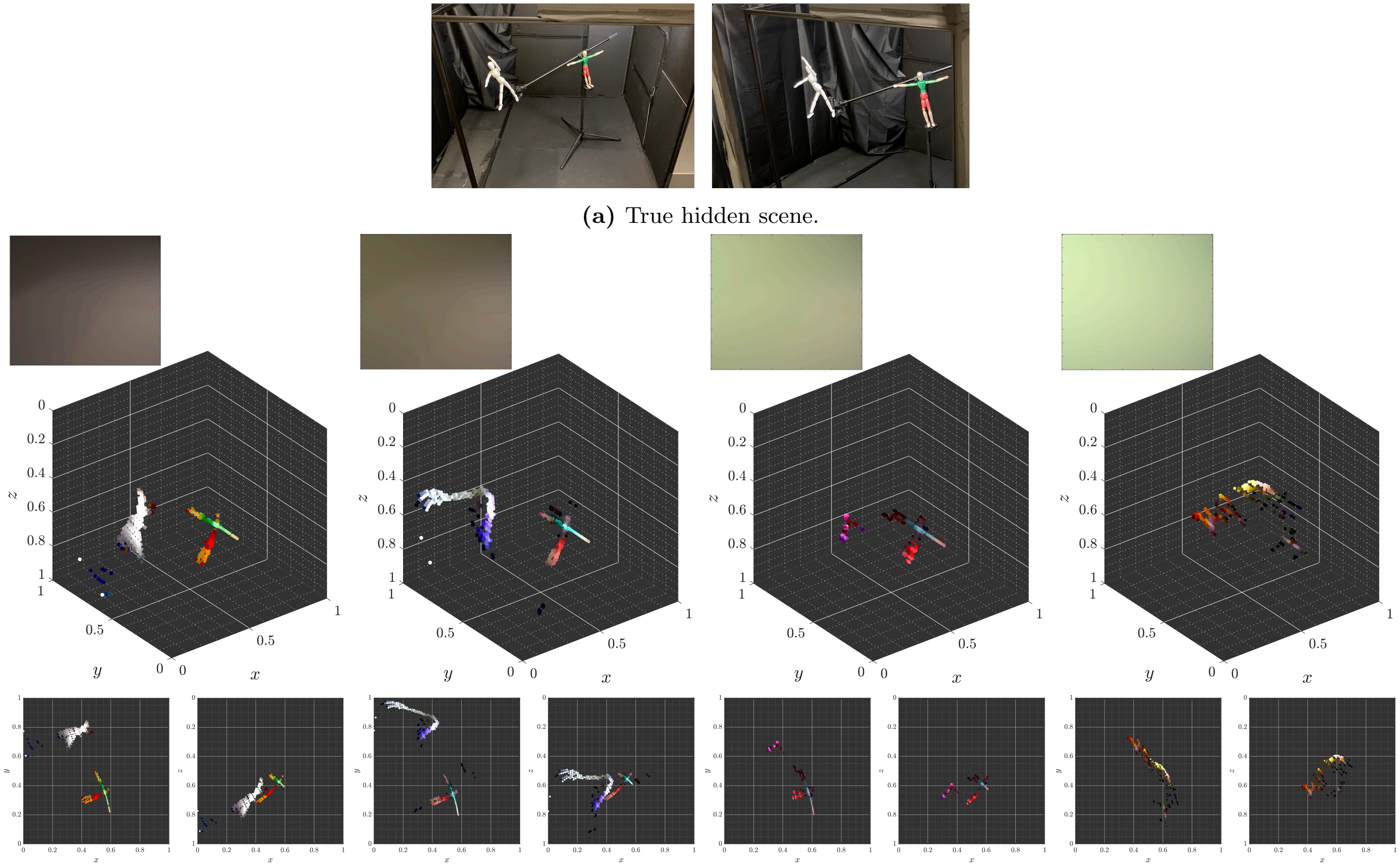

(a) True hidden scene.

(b) No additional ambient light. **(c)** Low ambient light. **(d)** Medium ambient light. **(e)** High ambient light.

Supplementary Figure 18: Reconstructions under additional ambient illumination. (a) The measured ranges of the two mannequins are 0.86 m (red-green mannequin in T pose) and 1.04 m (white mannequin in marching pose). (b) Accurate reconstruction when no additional ambient light is introduced. (c) Low level of additional visible side ambient light yields slightly degraded reconstruction with small amounts of clutter introduced. (d) Medium level of additional visible side ambient light yields significantly degraded reconstruction with a substantially higher amount of clutter introduced. Penumbra in the measurement is not easily visible to the human eye. (e) A failed reconstruction under a high level of additional visible side ambient light. Panels (b)–(e) show observations (top row), a 3D view of reconstructions (middle row), and xy- and xz-plane projections, respectively (bottom row).

S5.2.1 Single snapshot reconstructions with visible scene ambient illumination

The single snapshot reconstruction performance of the proposed approach is also investigated with measurements obtained with visible scene ambient lights turned on. The results demonstrate robustness even in these reduced SNR measurement cases shown in Supplementary Figure 19, the range estimates are still comparable to the fifty snapshot case and the visual reconstructions are similar in the low light case (Supplementary Figure 19(a)). However, Supplementary Figure 19(b) shows considerably worse shape recovery compared to its high SNR measurement counterpart (in Supplementary Figure 18(d)) with the marching mannequin no longer visible. However, the range reconstruction is still reliable.

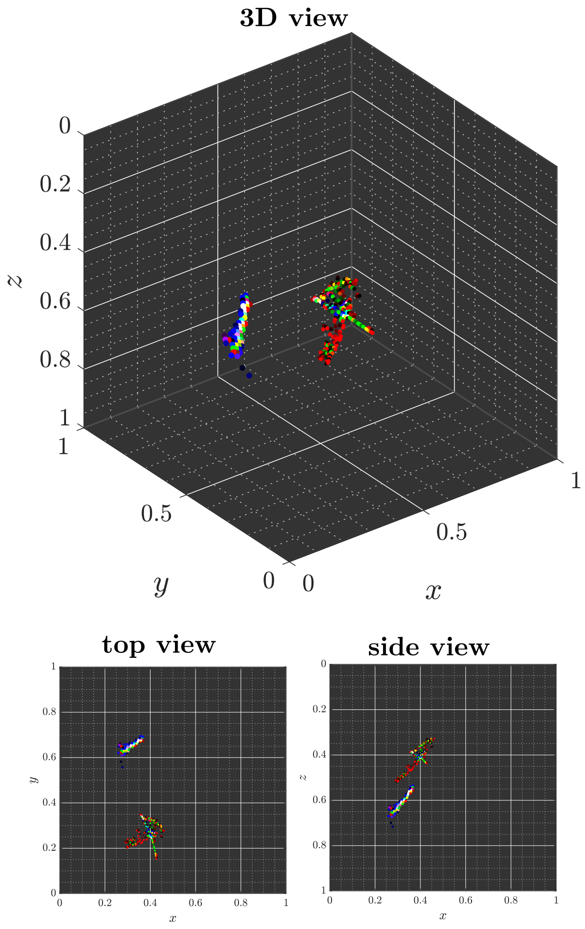

(a) Low ambient light.

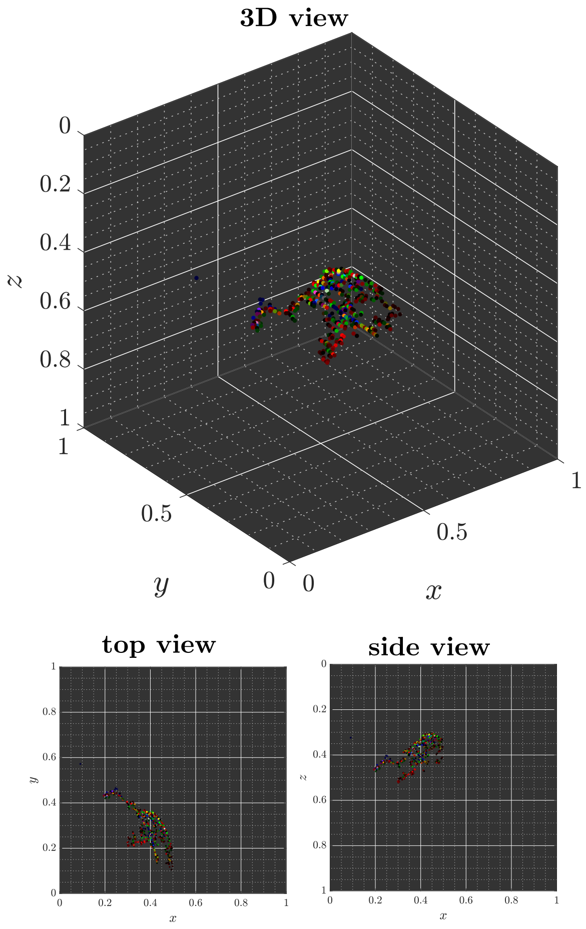

(b) Medium ambient light.

Supplementary Figure 19: Single snapshot reconstructions with visible scene ambient illumination turned on. Reconstructions obtained from a single exposure snapshot for the same two-mannequins scene in the main manuscript with visible scene illumination turned on. However, no averaging of multiple snapshots was performed to improve the measurement photograph's SNR. (a) The ranges of the mannequins are estimated to be 0.63 m for the T pose mannequin and .95 m for the marching mannequin. (b) The range of the mannequin in a T pose is estimated to be 0.63 m, while the marching mannequin was not recovered. In (b) the mannequin in the T pose was detected as two clusters, and the recovered ranges for both clusters were ~ 0.63 m.

S5.3 Single Object at Varying Ranges

To test the efficacy of the range reconstruction step of our approach, we created four scene setups that each contain a single plain white card. The ranges of the cards varied across the four setups, while their angular positions and extents (solid angle) were roughly constant. Note that to maintain the same angular extent at a different range, the objects must be physically larger at farther ranges. The ground truth photographs of the original scenes are shown in Supplementary Figure 20, and the 3D reconstructions and estimated ranges are shown in Supplementary Figure 21. While the range is not precise for all four scenes, they were reconstructed in the correct relative order. (We note that in this result, the person performing the data processing did not know the hidden scene and imaging conditions. Thus, there was only minimal tuning of the regularisation parameters.)

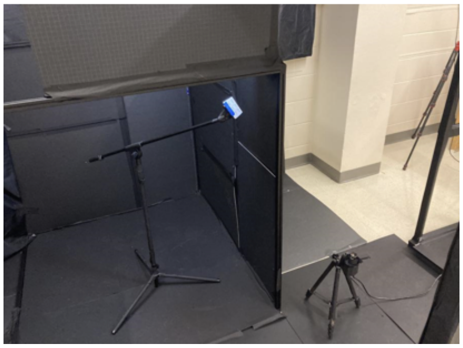

(a) Close (True range $\rho = .22\,\text{m}$).

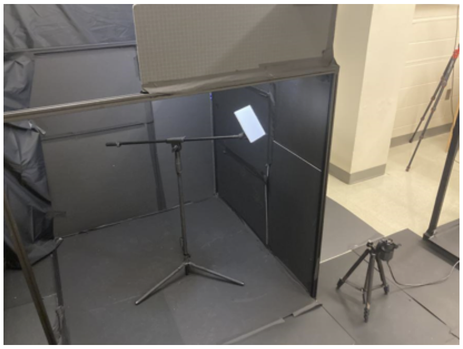

(b) Mid-Close (True range $\rho = 0.42\,\text{m}$).

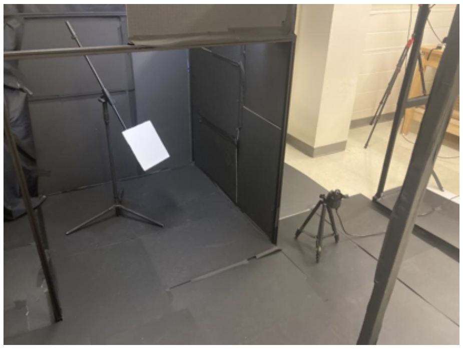

(c) Mid-Far (True range $\rho = 1.05\,\text{m}$).

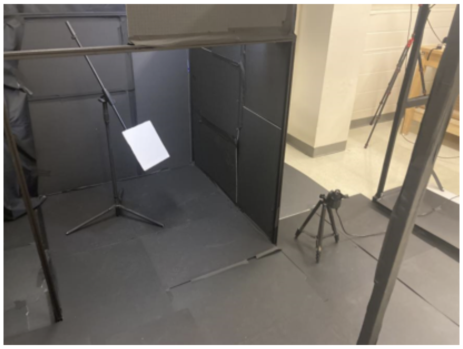

(d) Far (True range $\rho = 1.72\,\text{m}$).

Supplementary Figure 20: Ground truth photographs of square card scenes. Four square cards are placed at increasing ranges from the origin; the cards are sized such that they subtend roughly equal solid angles. The cards are placed at (a) close range, (b) mid-close range, (c) mid-far range, and (d) far range.

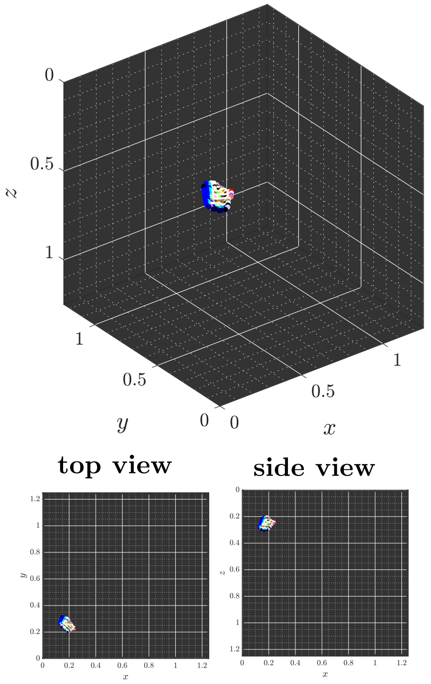

(a) Close (True range $\rho = 0.22\,\mathrm{m}$ and estimated range $\hat{\rho} = 0.41\,\mathrm{m}$).

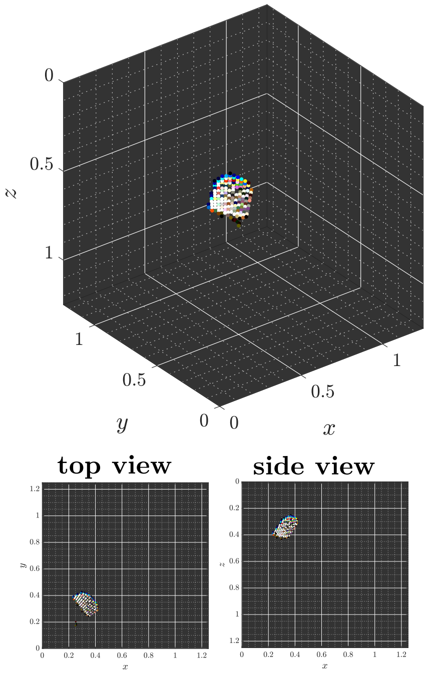

(b) Mid-close (True range $\rho = 0.42\,\mathrm{m}$ and estimated range $\hat{\rho} = 0.59\,\mathrm{m}$).

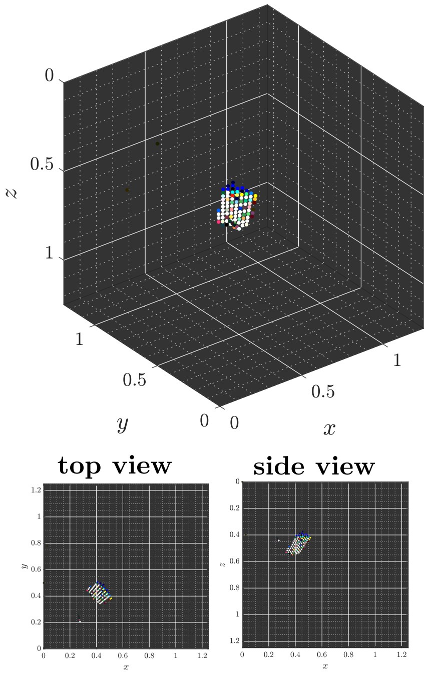

(c) Mid-Far (True range $\rho = 1.05\,\mathrm{m}$ and estimated range $\hat{\rho} = 0.77\,\mathrm{m}$).

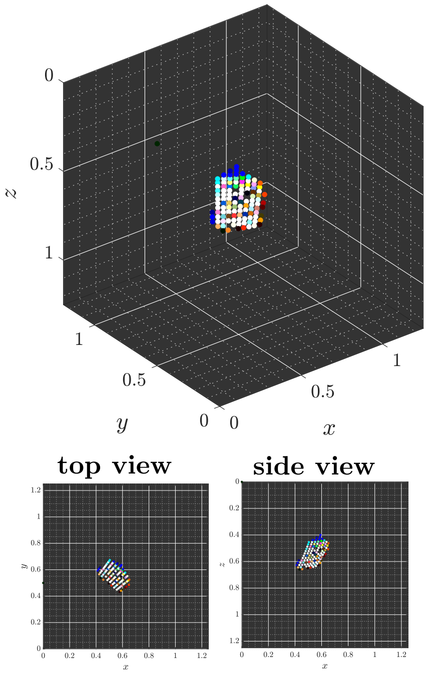

(d) Far (True range $\rho = 1.72\,\mathrm{m}$ and estimated range $\hat{\rho} = 0.96\,\mathrm{m}$).

Supplementary Figure 21: Reconstructions for targets with roughly equal solid angles placed at increasing ranges from the origin. The cards are placed at (a) close range, (b) mid-close range, (c) mid-far range, and (d) far range.

S5.4 Information orthogonality: An experimental demonstration

While there were no theoretical or practical constraints in selecting the initial range ρ_0 in Step 1 of TERI, all reconstructions in the main manuscript used an initial range ρ_0 of either 0.5 m or 1 m arbitrarily. The information orthogonality property of the proposed projected-elevation coordinate system allows accurate angular (i.e., shape) reconstruction with arbitrary initialisation of the range. This is a seemingly surprising phenomenon, which we further validate using real experiments.

Shown, in the figures that follow, are shape-only reconstructions (i.e., completing only Step 1 of the TERI algorithm) using various range initialisations between 0.1 m and 10 m. The reconstructions show almost no dependence on the chosen range values across two orders of magnitude. The most significant differences are increased amounts of clutter attempting to explain extraneous ambient illumination due to multiple reflections. (These differences become almost inconsequential when the entire TERI algorithm concludes. Also, we did not attempt to tune the regularisation parameter λ in these reconstructions, and the same value was used in almost all reconstructions. Thus, proper tuning of λ may further lessen the apparent differences.)

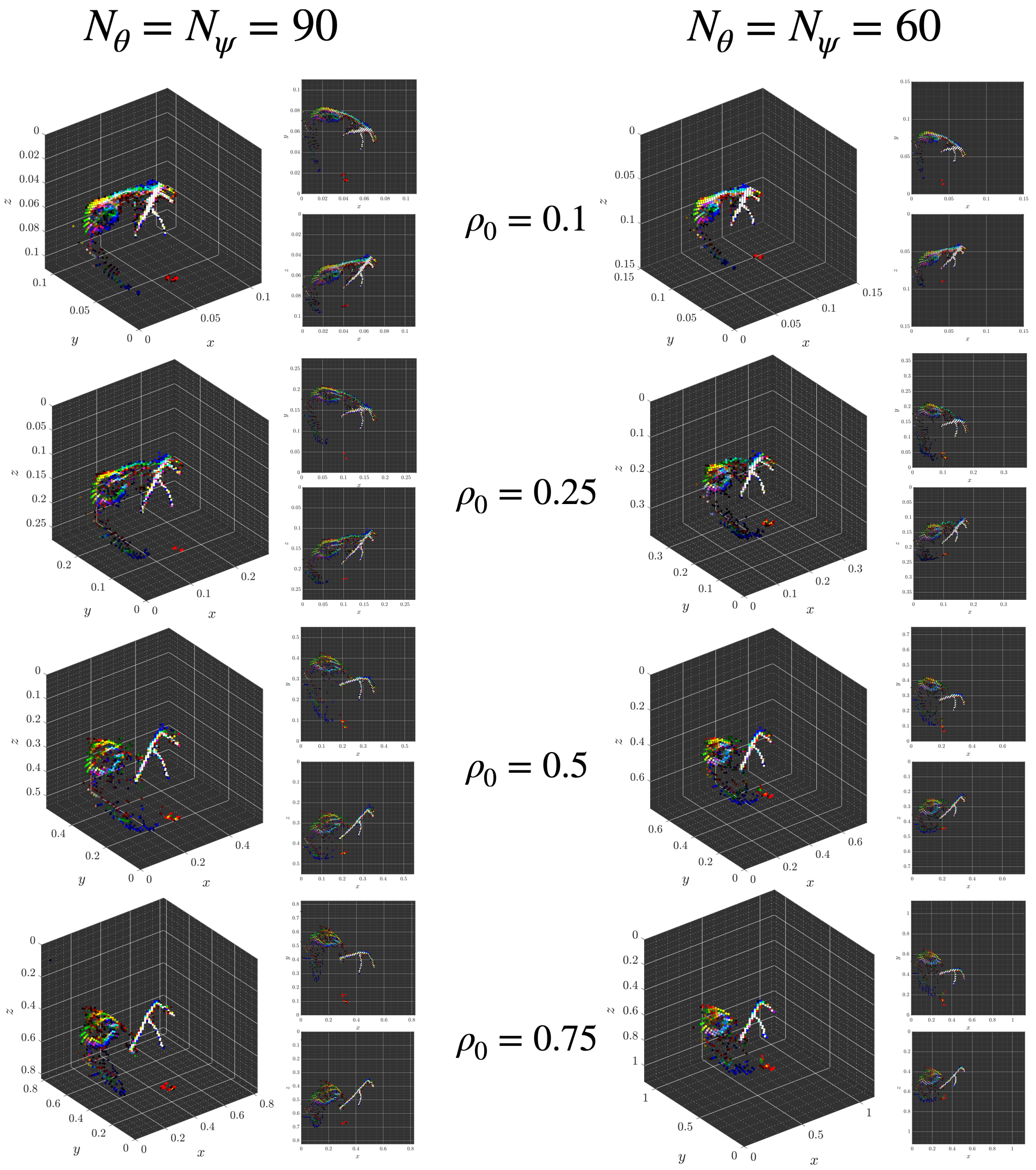

Supplementary Figure 22: Invariance of TERI reconstructions to arbitrary range initialization. Shape-only reconstruction results of solving the LIP in Step 1 of the TERI algorithm for different choices of initial range values (i.e., $\rho_0 = 0.1, 0.25, 0.5, 0.75$). Two different reconstruction resolutions are also shown: left is for $N_\theta \times N_\psi = 90 \times 90$ and right is for $N_\theta \times N_\psi = 60 \times 60$.

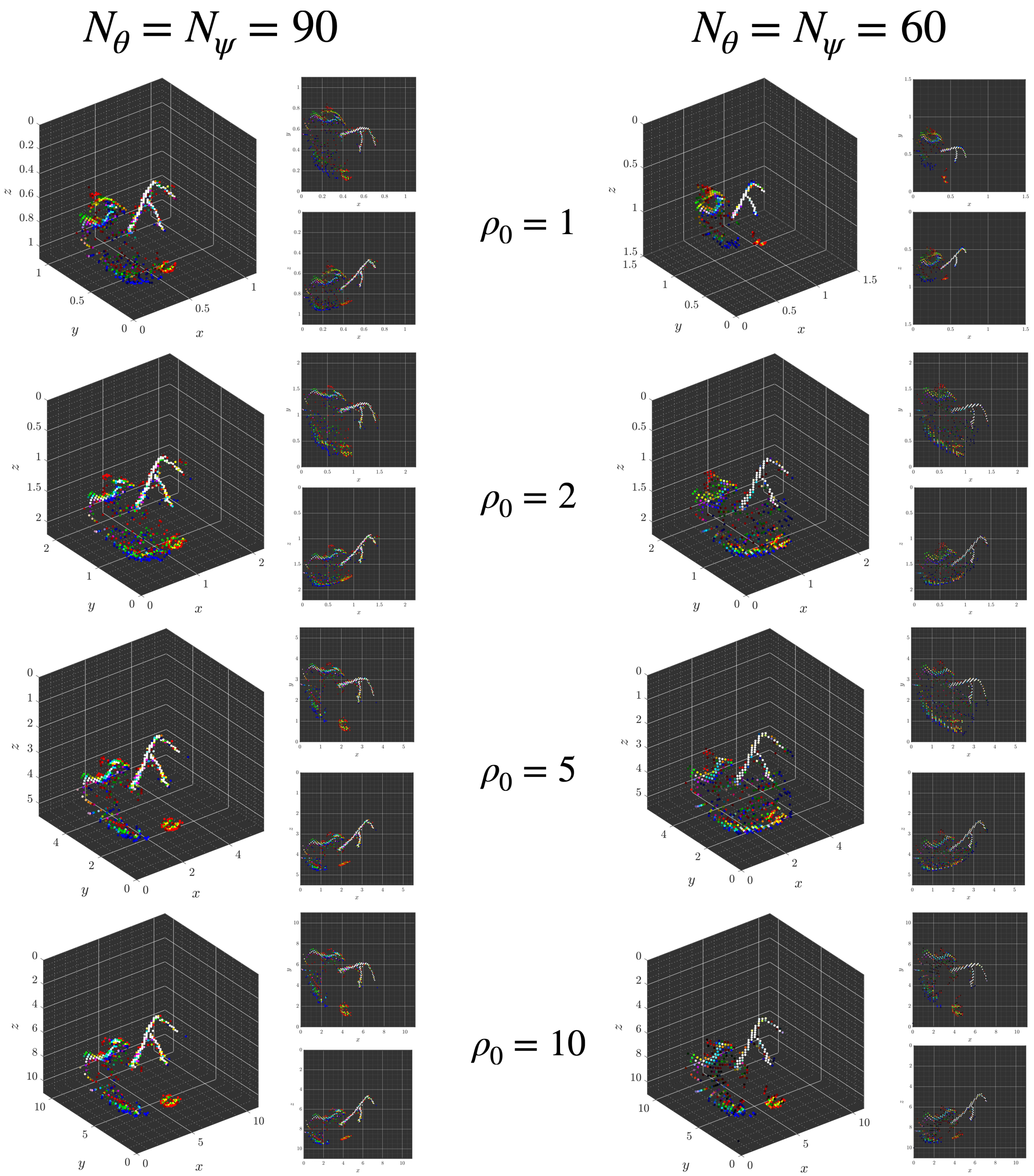

Supplementary Figure 23: Invariance of TERI reconstructions to arbitrary range initialization. Shape-only reconstruction results of solving the LIP in Step 1 of the TERI algorithm for different choices of initial range values (i.e., $\rho_0 = 1, 2, 5, 10$). Two different reconstruction resolutions are also shown: left is for $N_\theta \times N_\psi = 90 \times 90$ and right is for $N_\theta \times N_\psi = 60 \times 60$.

S5.5 Scenes that irradiate light from all angles

Large hidden scene objects, such as the shelf (see main manuscript Fig. 3) and planar surfaces like large walls, extend over a significant fraction of solid angles and vary continuously in range from the origin. If range estimation were well-conditioned, then estimating one range per solid angle (at high angular discretisation) would yield produce accurate visualisations for such large scenes. Because range estimation is poorly conditioned, however, the current work aims to attain reliable range estimates by clustering a possibly large number of neighbouring surface elements (recovered in Step I) and assuming they are confined to a single range. While this assumption improves the variance of reconstructed ranges, it also causes a bias in the estimated range when there is a significant mismatch (i.e., for large clusters). The computed 3D representation is a projection of the scene onto the surface of *projected-elevation sphere centered at the origin* (the 3D ball in projected-elevation spherical coordinates). Shown in Supplementary Figures 24 and 25 are photographs of the two scenes and their corresponding reconstructions demonstrating this phenomenon. The first scene is an empty room with white walls and floor, and the second is the same room with a white and blue broom (that partially occludes the walls).

The wall that is parallel to the doorway is 0.67 m from the doorway plane, the other wall is at 0.60 m from the xz-plane, and the floor is 1.24 m from the doorway head. The foamboard walls each are roughly 1 m high. Based on the surface area of each face of the room from the perspective of the ceiling, we may compute an "average range" by weighting the range of each surface by its distance from the origin. Thus:

- Surface area of hidden scene's wall 1 $\approx 0.6 \times 1 = 0.60$ m^2
- Surface area of hidden scene's wall 2 $\approx 0.67 \times 1 = 0.67$ m^2
- Surface area of hidden scene floor area $\approx 0.6 \times 0.67 = 0.402$ m^2
- Total surface area $0.60 + 0.67 + 0.402 = 1.672$ m^2
- Proportion of hidden scene contribution due to teach surface:
  1. *Wall 1:* $\approx \frac{0.60}{1.672} \approx 0.36$
  2. *Wall 2:* $\approx \frac{0.67}{1.672} \approx 0.40$
  3. *Floor:* $\approx \frac{0.402}{1.672} \approx 0.24$
- **Weighted average range $\approx$ $(\mathbf{0.36 \times 0.67}) + (\mathbf{0.40 \times 1.60}) + (\mathbf{0.24 \times 1.24}) \approx \mathbf{0.78}$ m**

This weighted average range of 0.78 m can be interpreted as a rough estimate of the range a single projected elevation spherical shell would have to be to approximate all surfaces of the room collectively. This estimate very closely matches the estimated range of 0.79 m computed using the proposed TERI reconstruction algorithm for the configuration shown in Supplementary Figure 24. In the second scene, Supplementary Figure 25, the presence of the broom marginally decreases the weighted average range for the scene, and correspondingly the reconstructed range produced in Supplementary Figure 25(b) is 0.76 m.

Visually, we can readily conclude that almost all solid angles are contain an object. However, it is difficult to intuit from the visualisations in Supplementary Figures 24(b) and 25(b) that they are reconstructions of rooms. This highlights a limitation of the clustering approach proposed in this work. To address this limitation, one possible approach is to augment the current modeling with a parametric model to account for walls and floors, allowing three planes each one with a distance to be computed in Step 2 of the reconstruction algorithm. This is not a trivial extension since mutual occlusions among the planes have to also be incorporated into the model. Another approach is to deproject the reconstructions in Supplementary Figures 24(b) and 25(b) onto three orthogonal planes (corresponding to the two walls and floor planes) and exploiting the shadows and variations in color present in the reconstructions as cues for separating background and foreground objects. Finally, we anticipate that refining clusters into smaller sizes could yield more interpretable reconstructions and visualisations. In particular, a large cluster could be successively segmented into smaller sub-clusters while also refining their range estimates. Assuming successive sub-clusters remain sufficiently large to make the variance of their range

estimates small enough. This approach will improve biases in range estimates for large objects while also facilitating the separation of foreground and background objects that may have been incorrectly clustered in initial reconstructions. Section S7 presents actual experimental results that demonstrate the feasibility of splitting a cluster that contains two objects that are neighbours in angle but have different ranges.

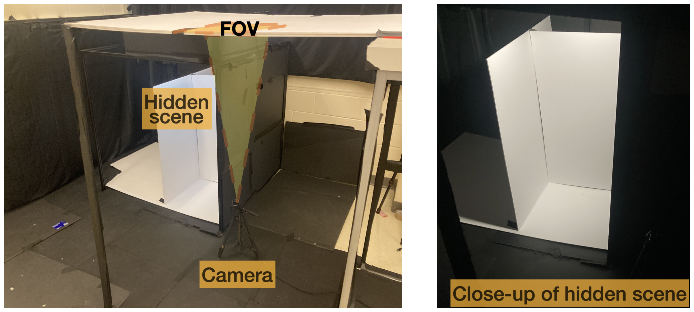

(a) Photographs showing different views of ground truth hidden scene.

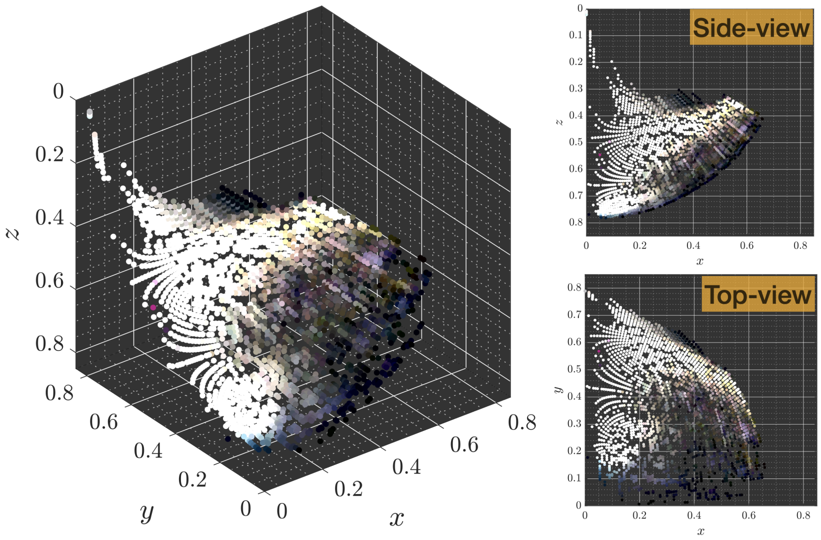

(b) Different views of the reconstructed scene.

Supplementary Figure 24: Example reconstruction of a non-sparse scene. (a) The hidden scene is an empty room with white walls and floor. The wall parallel to the doorway is placed at 0.67 m from the doorway plane, the other wall is at 0.60 m from the xz-plane, and the floor is 1.24 m from the doorway head. (b) 3D view, side, and top views of the computed reconstruction. The entire scene is reconstructed as a single large cluster of surface elements with an estimated range of 0.79 m. One of the walls is reconstructed as being brighter than the other because the hidden scene illumination was facing that wall.

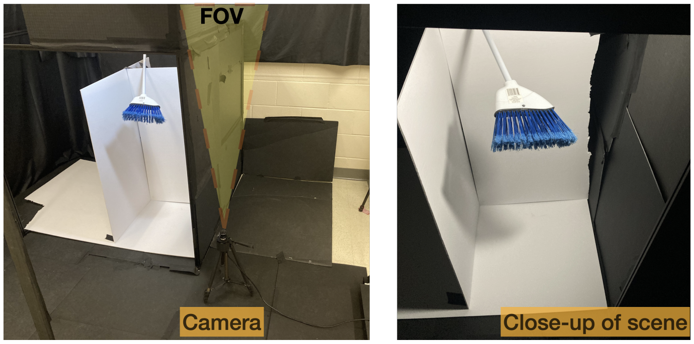

(a) Photographs showing different views of ground truth hidden scene.

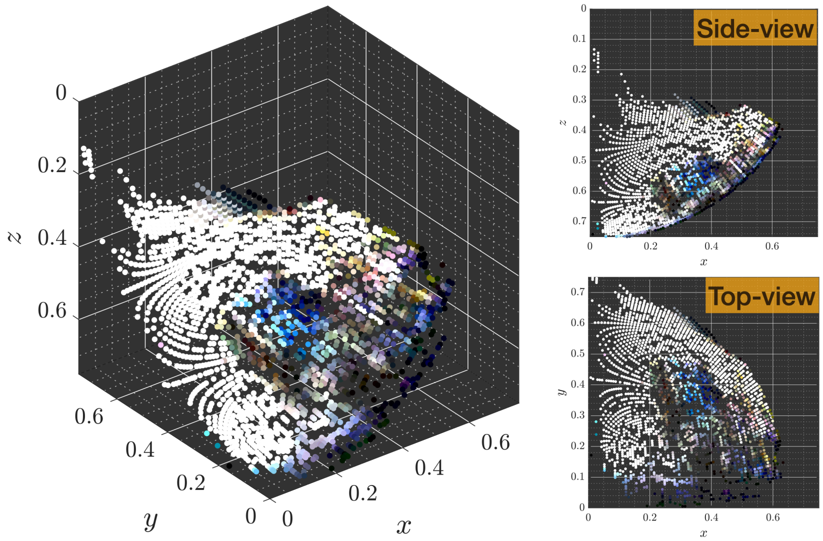

(b) Photographs showing different views of ground truth hidden scene.

Supplementary Figure 25: A non-sparse scene with partial occlusion. (a) The hidden scene configuration, which shows a room with a white and blue broom inside it. From the perspective of the ceiling plane, the broom casts a shadow on some portions and fully occludes some other portions of the room's walls and floor. The wall parallel to the doorway is placed at 0.67 m from the doorway plane, the other wall is 0.60 m from the xz-plane, the floor is 1.24 m from the doorway head, while the broom is roughly 0.53 m from the origin. (b) The obtained reconstruction of the scene shows a 3D view along with side and top views. The entire scene is reconstructed as a single large cluster of surface elements with an estimated range of 0.76 m. The blueness and orientation of the broom's bristles and part of the shadow cast by the broom are visible in the reconstructions.

Supplementary Note 6

S6 Alternative reconstruction algorithms

The integral model,

$$\begin{aligned} y_m &= \sum_{n=1}^{N_\theta N_\psi} f_n \int_{\mathbf{s}\in\mathcal{S}_n} \ell(\mathbf{p}_m;\rho,\theta,\psi)\delta(\rho-\rho_n)\frac{\rho^2\sec^2(\theta)\sec^2(\psi)}{(1+\tan^2(\theta)+\tan^2(\psi))^{\frac{3}{2}}}\,\mathrm{d}\mathbf{s} \\ &= \sum_{n=1}^{N_\theta N_\psi} f_n \int_{\theta_n-\delta_\theta}^{\theta_n+\delta_\theta}\int_{\psi_n-\delta_\psi}^{\psi_n+\delta_\psi} \ell(\mathbf{p}_m;\rho_n,\theta,\psi)\frac{\rho_n^2\sec^2(\theta)\sec^2(\psi)}{(1+\tan^2(\theta)+\tan^2(\psi))^{\frac{3}{2}}}\,\mathrm{d}\psi\,\mathrm{d}\theta, \end{aligned} \tag{74}$$

which appears as equation (3) of the main manuscript relates the hidden scene to the measured intensity photograph. In principle, equation (74) is invertible by a number of possible approaches to reconstruct the hidden scene. Among all possibilities, we investigated four main approaches but presented in the main manuscript only the most computationally efficient, robust, and accurate approach, which is also justified by our theoretical FI and CRB analyses (detailed in Section S2).

That main reconstruction approach assumed the entire hidden scene to be confined to a single range and first reconstructs a 2D (azimuth and projected-elevation) angular representation of the scene and clusters the recovered angular surface elements (based on their proximity). Subsequently, a non-linear model is inverted to compute a new range for each identified cluster of surface elements. In the first step, the 2D angular dimensions were finely discretized because our analyses predict high angular resolution (compared to the range resolution). In the second step, the range coordinate is not discretized; instead, we solve for continuous-valued ranges given the *a priori* computed clusters of surface elements. This step is motivated by attempting to improve the conditioning of the range reconstruction problem, by combining a number of (potentially) spatially contiguous surface elements into a cluster and seeking to estimate only a single range for that cluster. The (naïve) alternative of this approach is to independently estimate a range for each angular surface element reconstructed in the first step without clustering them: This alternative is investigated in Section S6.1.

Additionally, the following two related alternatives are also evaluated:

1. The range coordinate is discretized in the second step to yield a discretised linear model, while keeping the sequential structure of the main algorithm (see Section S6.2).

2. The range and angular coordinates are both discretized in (74) and both are reconstructed jointly in a single step (see Section S6.3).

S6.1 Inverting a Non-linear Model for Range without Clustering

Here we provide additional experiments to empirically demonstrate the effectiveness of the clustering step. In this case, we simply bypass the clustering procedure that terminates step one of the TERI reconstruction algorithm and proceed with inverting the non-linear range model. Thus, a range estimate will be computed for each surface element—estimated as having nonzero radiosity in step one—without any spatial coupling/correlation being imposed. Essentially, instead of having a few clusters for which a single range will be computed, the general setting where each surface element is treated individually and a proximal gradient method is used to recover a continuous-valued range for that surface element. The obtained reconstructions for the USF and the two-mannequin scenes are shown in Supplementary Figures 26 and 27. Note that the colour, angles-only reconstructions of step one (shown in Supplementary Figures 26(a) and 27(a)), will be the same as the reconstructions from step one of the proposed TERI algorithm; however, their final results will potentially differ after step two because of the exclusion of the clustering step here.

As can be seen in Supplementary Figures 26(b) and 27(b), the range estimates for the surface elements constituting the scenes become spread out over a broad range of values (from zero to a maximum of

around 4.8 m), due to the poor conditioning of the range estimation problem. Thus, because of this high inaccuracy of the range estimates, the shape of the scene is no longer apparent in the final reconstructions unless viewed from a specific vantage point. The clustering step essentially imposes a strong spatial correlation prior among neighbouring surface elements for a scene. A total variation constraint in the second step may potentially impose the required spatial regularity in the range to allow stable, and flexible range estimation. However, we anticipate that this will increase computational complexity.

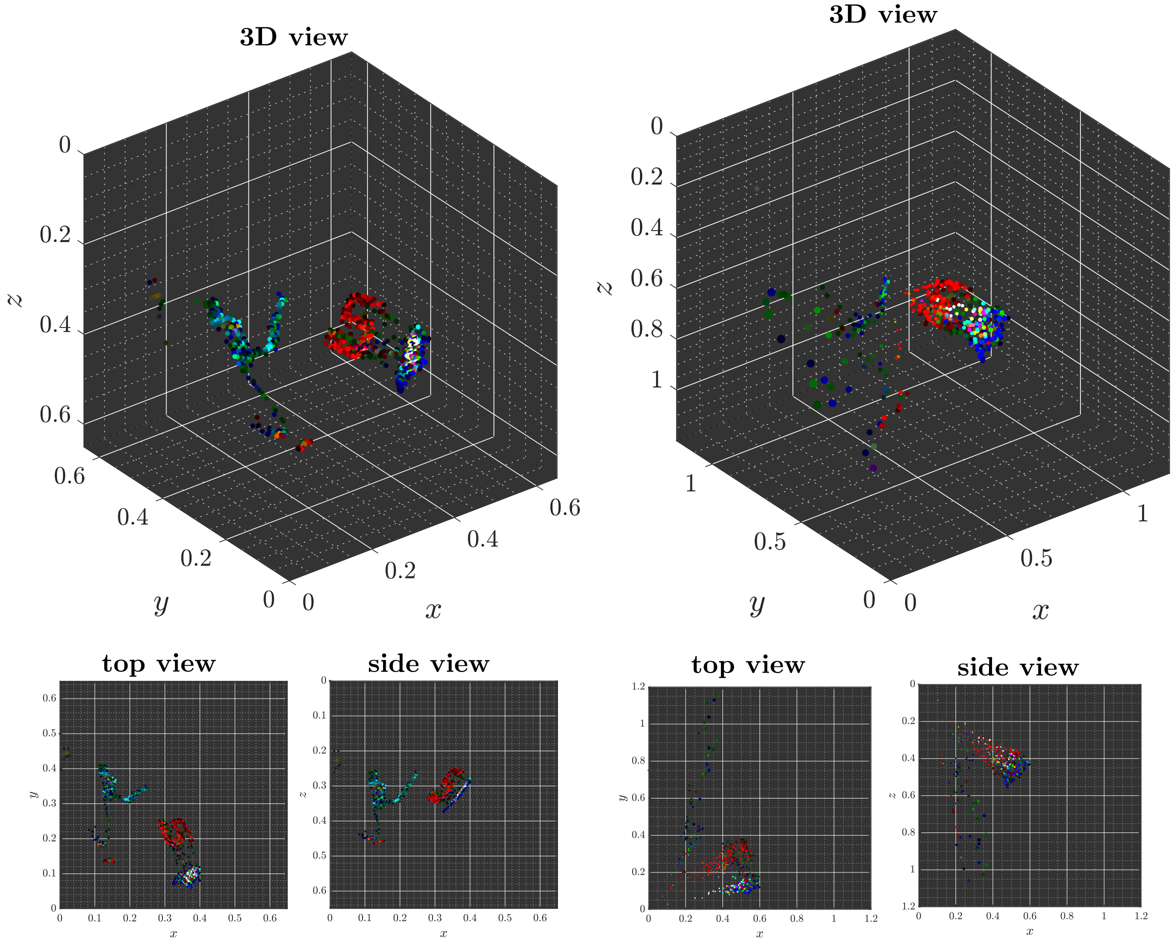

(a) Reconstructions when step one terminates. **(b)** Final reconstructions when clustering is bypassed.

Supplementary Figure 26: Two-step reconstruction results without the clustering step for the USF scene. Here the two-step reconstruction algorithm is used to recover a 3D image of the USF scene, with the clustering step excluded from the approach. This amounts to seeking to reconstruct a range for each surface element estimated in step one (shown in (a)) without exploiting any spatial (range) correlation priors. (a) Reconstruction obtained after step one. (b) Final reconstruction after step two when the clustering step is bypassed.

S6.2 Linear Model Inversion: Sequential Reconstruction

To investigate the effectiveness of using clustering in our reconstruction method to improve the conditioning of the range, we attempt to reconstruct some scenes while omitting the clustering step (i.e., the terminal stage of step one in Figure 2 of the main manuscript). As in the main manuscript, we start by choosing a fixed $\boldsymbol{\rho}_0$ and then estimating $\mathbf{f}$—which is a discretized representation of the scene along

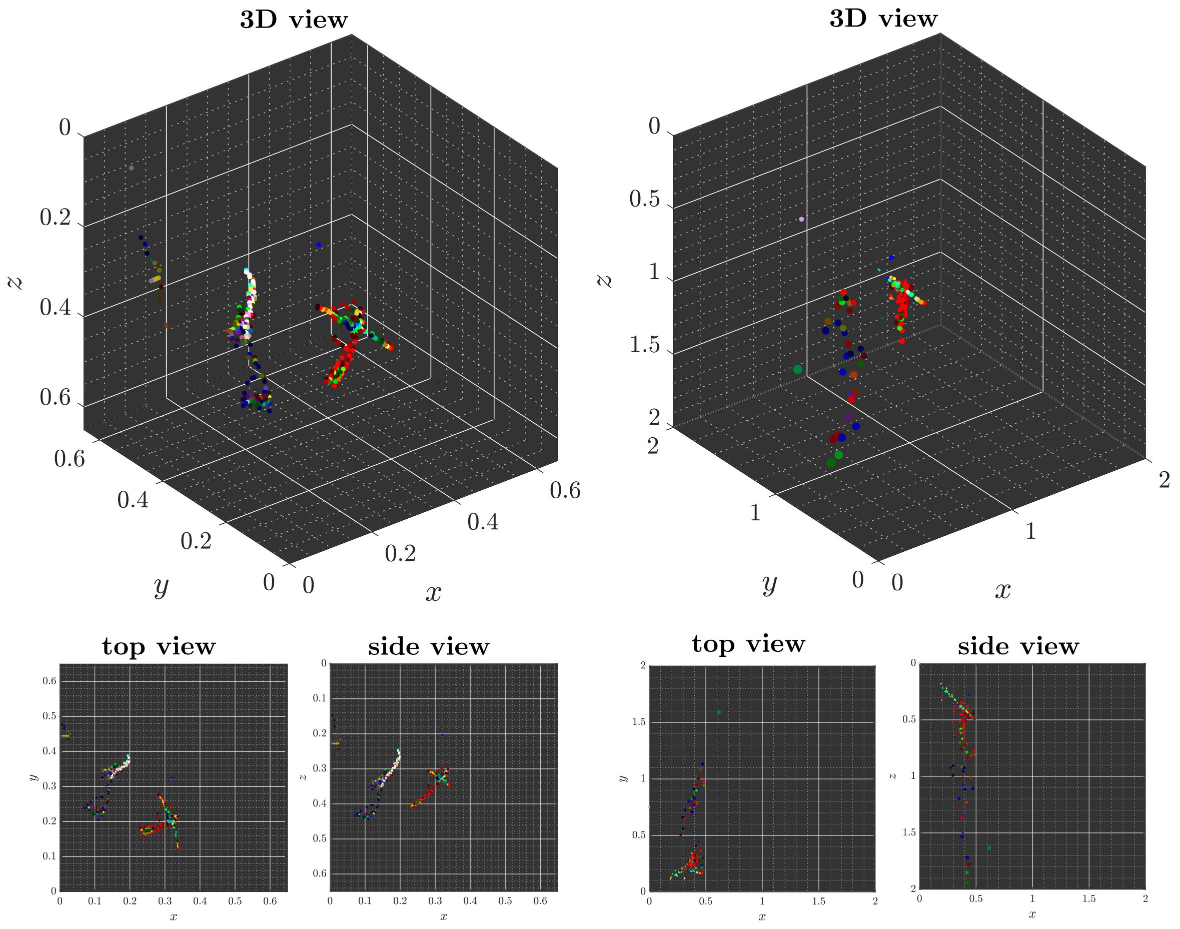

(a) Reconstructions when step one terminates. **(b)** Final reconstructions when clustering is bypassed.

Supplementary Figure 27: Two-step reconstruction results without the clustering step for the two mannequins scene. Here, the two-step reconstruction algorithm is used to recover a 3D image of the two mannequins scene, with the clustering step excluded from the approach. This amounts to seeking to reconstruct a range for each surface element estimated in step one (shown in (a)) without exploiting any spatial (range) correlation priors. (a) Reconstruction obtained after step one. (b) Final reconstruction after step two when the clustering step is bypassed.

the azimuth and projected-elevation angles axes alone—from the forward model $\mathbf{y} = \mathbf{A}(\boldsymbol{\rho}_0)\mathbf{f} + \mathbf{v} + \mathbf{n}$, by solving:

$$\arg \min_{(\mathbf{f},\mathbf{b})\geq \mathbf{0}} \|\mathbf{y} - \mathbf{A}(\boldsymbol{\rho_0})\mathbf{f} - \mathbf{B}\mathbf{b}\|_2^2 + \lambda \|\mathbf{f}\|_1 . \tag{75}$$

Given the estimate for $\mathbf{f}$, we then construct a new matrix $\mathbf{A}_{\mathrm{r}}$ at multiple ranges, which model only contributions from surface elements that were estimated as having non-zero radiosities in the solution to (75) (i.e., Step 1 without clustering). Assume there were J computed angular surface elements from the first step, for each of them we discretize the range axis into K possible candidates $\{\rho_k\}_{k=1}^{K}$, and construct the matrix $\mathbf{A}_{\mathrm{r}}$ that has JK number of columns (and M number of rows) such that:

$$\begin{aligned}\mathbf{y}_{\text{grey}} =& \sum_{k=1}^{K}\sum_{j=1}^{J} w_{j,k} \int_{\psi_j-\delta_\psi}^{\psi_j+\delta_\psi}\int_{\theta_j-\delta_\theta}^{\theta_j+\delta_\theta} \ell(\mathbf{p}_m;\rho_k,\theta,\psi)\frac{\rho_k^2 \sec^2(\theta)\sec^2(\psi)}{(1+\tan^2(\theta)+\tan^2(\psi))^{\frac{3}{2}}}\, \mathrm{d}\theta\, \mathrm{d}\psi + \mathbf{v} + \mathbf{n}\\ =& \mathbf{A}\,\mathbf{w} + \mathbf{B}\mathbf{b} + \mathbf{n}.\end{aligned} \tag{76}$$

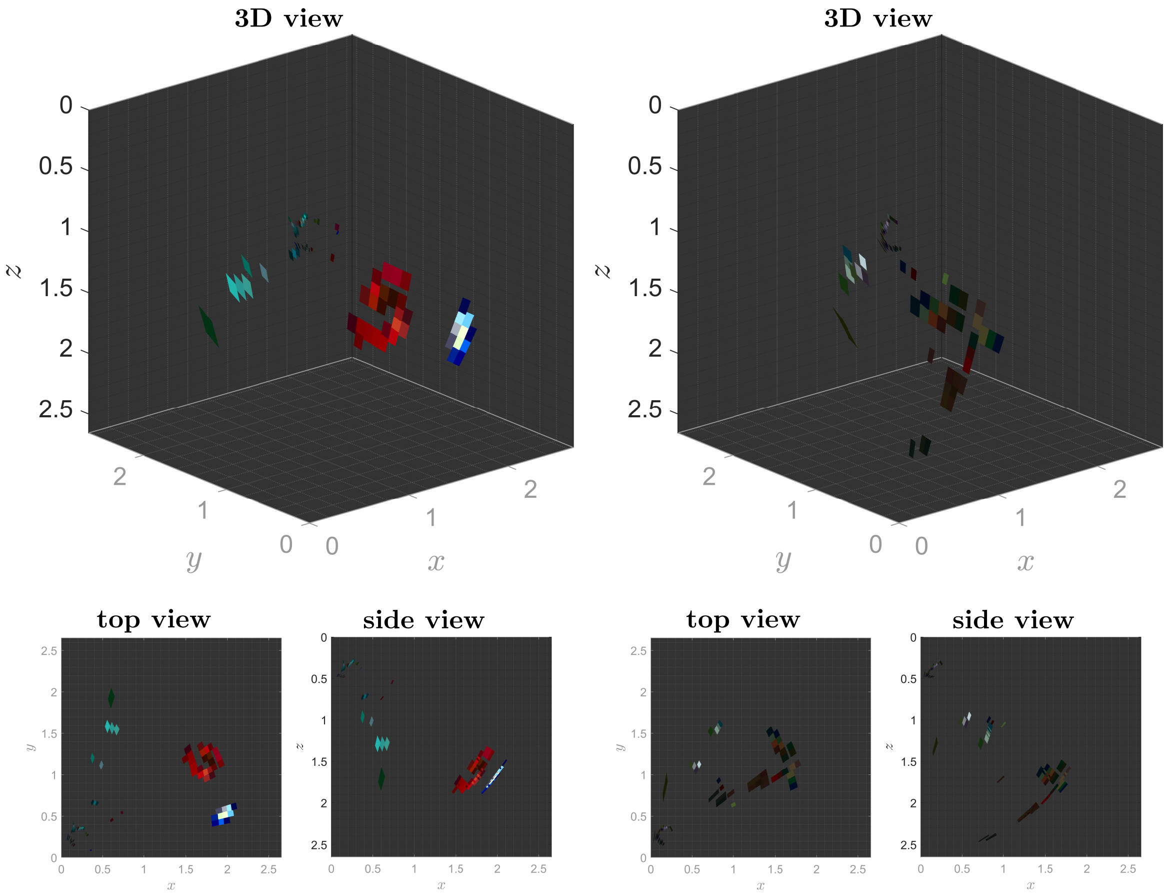

(a) USF Scene **(b)** Two mannequins scene

Supplementary Figure 28: Full 3D Reconstructions using a linear model for range and without applying clustering. Reconstructions are obtained by omitting the clustering step and, instead, ranges for each angular facet are computed individually, without spatial priors. (a) The S and F were both recovered correctly, but the U was broken into many pieces. (b) The mannequins were both broken into many pieces and placed in many different range bins. These results demonstrate the importance of exploiting spatial correlations to improve upon range estimates.

Then, we can estimate $\mathbf{w}$ which will produce a 3D reconstruction with each solid angle now allowed to potentially have multiple surface elements (each with a different range). In practice, the final reconstruction is computed as an α-trimmed mean of the estimate recovered from solving an ℓ_1-regularized inverse problem for (76). This variant (of the original TERI reconstruction algorithm) essentially interprets the solution of Step 1 (without clustering) as indicating which solid angles contain hidden scene objects. Then computes a set of plausible ranges for each solid angle by discretizing the forward model along the range axis, only for the solid angles identified as containing an object. Finally, a post-processing step is used to enforce that each solid angle contains a single object at a single range. Reconstructions for the USF and two-mannequins scenes are shown in Supplementary Figure 28. The USF scene is still recognizable, but because we do not cluster them the range reconstruction is very poorly conditioned, and all three letters have surface elements that are predicted to be at various ranges. The U is especially broken up to the point where the entire shape of the object is not obvious. The two mannequins scene is also apparently visually inaccurate, as the objects are broken up along the range axis into many pieces and do not form coherent, recognizable objects.

S6.3 Linear Model Inversion: Joint Non-sequential Reconstruction

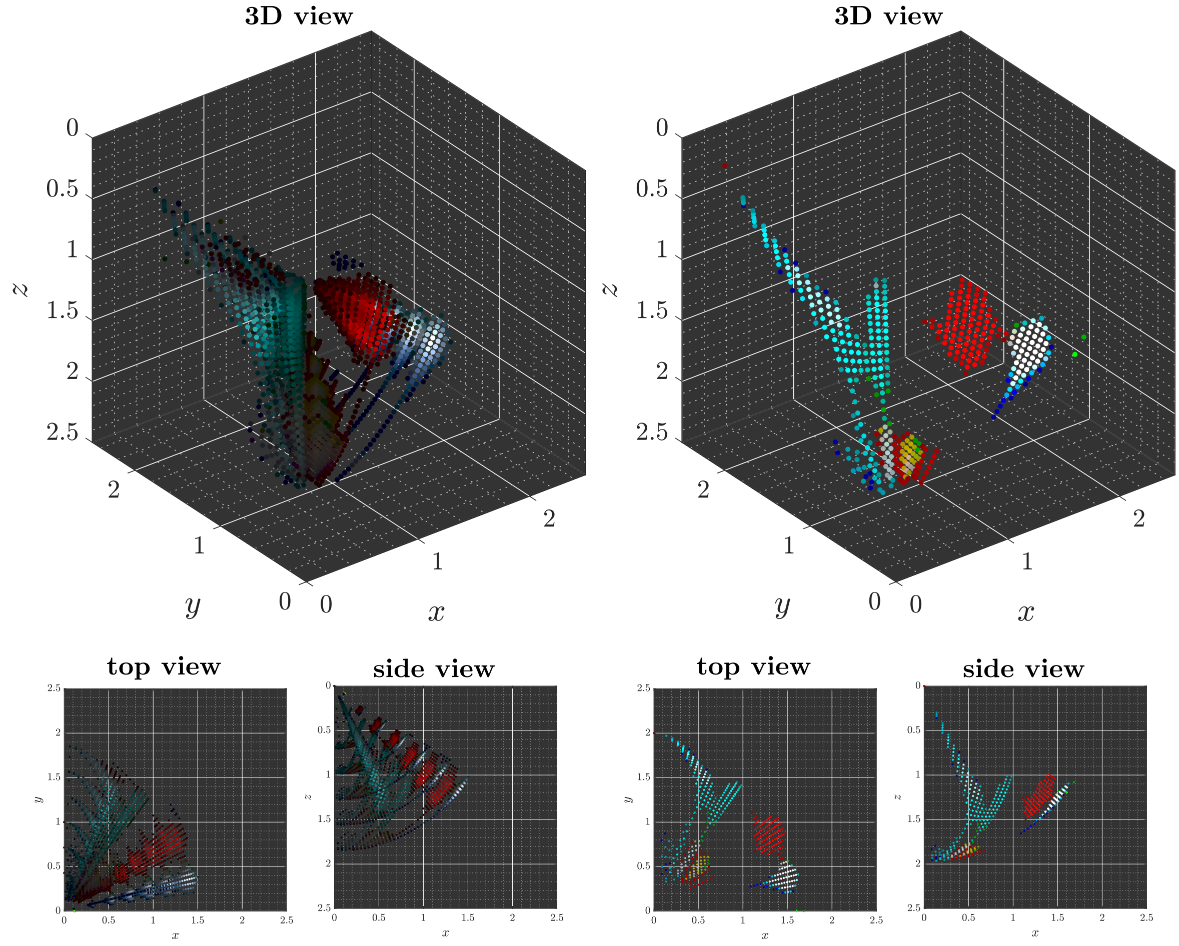

(a) Scene reconstruction. **(b)** Reconstruction averaged along the range axis.

Supplementary Figure 29: Joint non-sequential inversion of the fully linear forward model for USF scene. (a) The scene is reconstructed with significant blurring in range, as it is estimated to occupy many ranges bins (specifically range bins 2 through 7, as shown in Supplementary Figure 30). (b) The reconstruction in (a) is averaged along the range bins, and displayed with an arbitrarily chosen range of $\rho = 2$ m.

In this approach, the model (74) is discretized along the range coordinate as well, such that the entire hidden scene volume is represented as a collection of $N_\rho \times N_\theta \times N_\psi$ surface elements, with equal N_ρ uniformly-spaced range values $\{\rho_r\}_{r=1}^{N_\rho}$ in the interval $[0, R_{\max}]$, where $R_{\max}$ is an *a priori* chosen maximum possible scene range. This uniform discretization in the 3D projected-elevation spherical coordinate system produces the discrete linear model:

$$\mathbf{y} = \mathbf{G}\mathbf{h} + \mathbf{v} + \mathbf{n}, \tag{77}$$

where the (m, i) element of $\mathbf{G} \in \mathbb{R}^{M \times N_\rho N_\theta N_\psi}$ is given by,

$$[\mathbf{G}]_{m,i} = \int_{\theta_i - \delta_\theta}^{\theta_i + \delta_\theta} \int_{\psi_i - \delta_\psi}^{\psi_i + \delta_\psi} \ell(\mathbf{p}_m; \rho_i, \theta, \psi) \frac{\rho_i^2 \sec^2(\theta) \sec^2(\psi)}{(1 + \tan^2(\theta) + \tan^2(\psi))^{\frac{3}{2}}} \, d\psi \, d\theta, \tag{78}$$

the i-th element of $\mathbf{h} \in \mathbb{R}^{N_\rho N_\theta N_\psi}$, i.e. h_i, now represents the radiosity of surface element i whose

center is $(\rho_i, \theta_i, \psi_i)$, while $\mathbf{v}$ and $\mathbf{n}$ model visible ambient light and additive noise contributions, respectively. Note that for this discrete 3D grid, the i-th position $(\rho_i, \theta_i, \psi_i)$, with $i = 1, 2, \ldots, N_\rho N_\theta N_\psi$, now identifies the centre of the potential hidden scene surface element $\mathcal{S}_i = \{(\rho_i, \theta, \psi) : \theta \in [\theta_i - \delta_\theta, \theta_i + \delta_\theta],$ and $\psi \in [\psi_i - \delta_\psi, \psi_i + \delta_\psi]\}$ whose range from the origin is ρ_i. To recover the hidden scene, we solve the following l_1-constrained optimization problem:

$$\arg\min_{\mathbf{h}} \|\mathbf{y} - \mathbf{G}\mathbf{h}\|_2^2 + \|\mathbf{h}\|_1, \tag{79}$$

using the fast iterative shrinkage thresholding algorithm (FISTA) [11]. Because the range and two angular coordinates are reconstructed jointly, this approach is not amenable to clustering. In addition, a good representation needs N_ρ, N_θ, and N_ψ to be large. However, because the range resolution is comparatively lower than the resolution along the two angular coordinates, the range axes cannot be discretized quite as finely. Doing so would otherwise lead to a poorly conditioned forward operator $\mathbf{G}$. Moreover, large N_ρ, N_θ, and N_ψ also increases computational complexity significantly. For computational complexity, we choose $N_\rho = 10$ with ranges uniformly distributed in $[0, 2]$ m, $N_\theta = 45$, and $N_\psi = 45$. The experimental configuration (camera placement, FOV, number of measurements M, and so on) is otherwise the same as the one appearing in the results and methods sections of the main manuscript. Under these conditions, the reconstruction for the USF scene obtained by this alternative non-sequential joint recovery approach is shown in Supplementary Figures 29 and 30, as well as the reconstruction for the two mannequins scene (with no additional ambient light introduced) is shown in Supplementary Figures 31 and 32. The reconstructions are displayed as point clouds, with locations corresponding to the centers of each surface element, and colour represented by the corresponding estimated radiosities rescaled for display. Due to the poor range resolution compared to angular resolution, as seen in Supplementary Figures 29(a) and 30, the range localization is poor and the scene is estimated to occupy many ranges even for each angular bin. (No mutual occlusion is modelled.) This spreading may be potentially alleviated by using sparse group lasso and imposing (one-)sparse groups in each solid angle (i.e., aggressively sparsifying the estimates along the range axis). Supplementary Figure 29(b) visualizes the estimates averaged along the range axis. While the U in USF is recognizable as such, the S and the F are blurred, however. This is partly due to the coarser discretizations in the angular axes used and partly due to naïve reconstruction approach. Because the forward operator is much less well-conditioned, convergence is much slower. The coarser angular discretization also makes the pose of the marching mannequin nearly impossible to discern, and the T-pose held by the other mannequin is much clearer. The colour reconstruction fidelity achieved in both scenes (i.e., Supplementary Figures 29 to 32) is surprisingly accurate. However, more clutter near the origin of the xy-plane is observable in these reconstructions compared to the reconstruction approach used in the main manuscript.

For improved range reconstructions, a weighted average range—with weights based on the estimated intensity along the range axis of each solid angle—could be computed to compute a single overall range estimate per solid angle. This computation can also be extended to clusters of surface elements. Regardless, initial estimates computed non-sequentially will still be plagued by increased computational complexity due to the curse of dimensionality.

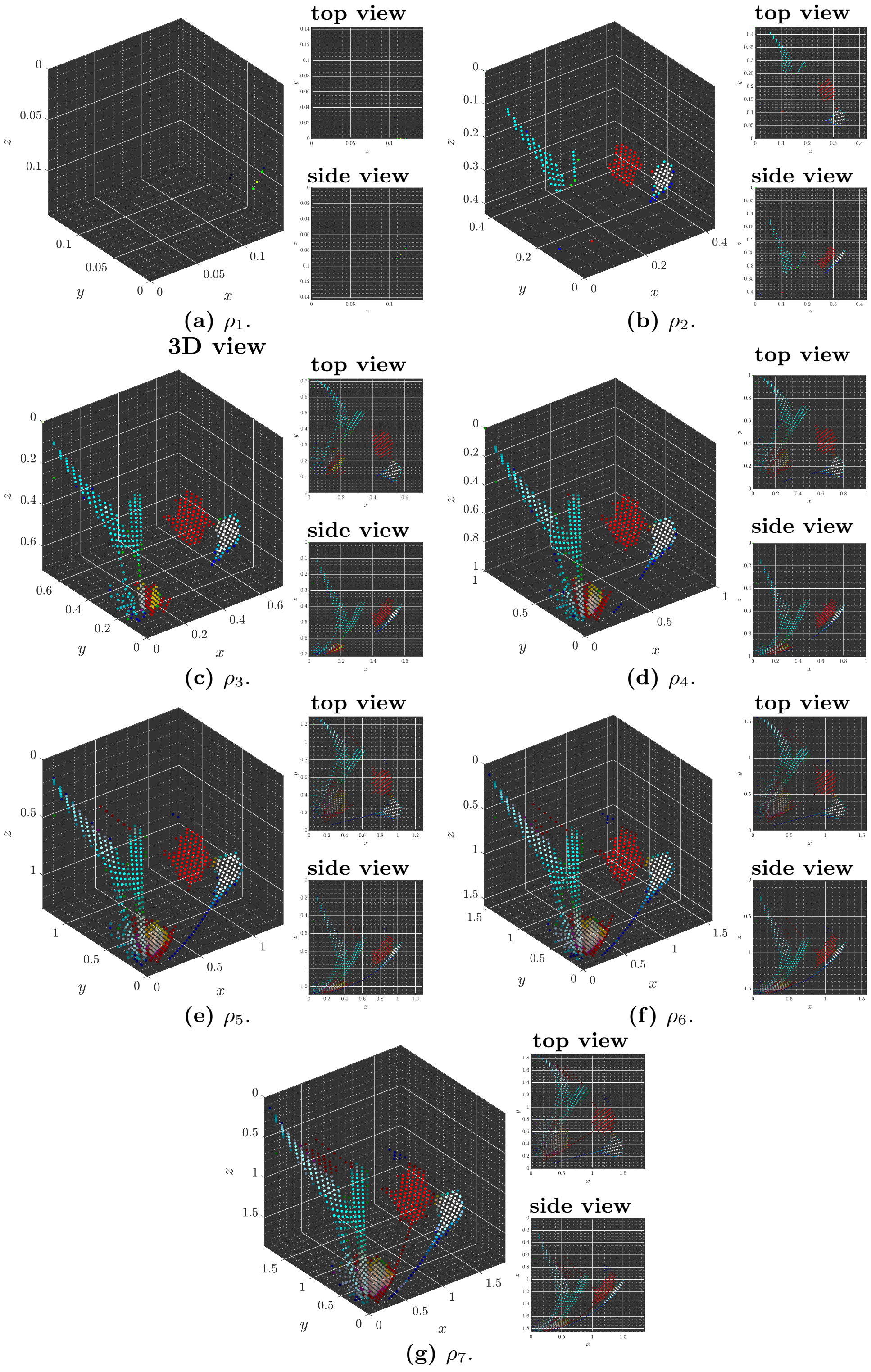

(a) ρ_1. (b) ρ_2. (c) ρ_3. (d) ρ_4. (e) ρ_5. (f) ρ_6. (g) ρ_7.

Supplementary Figure 30: Joint non-sequential inversion of the fully linear forward model for USF scene. The radiosities estimated for range bins $\rho_1, \rho_2, \ldots, \rho_7$ are shown in panels (a) to (g), respectively. All range bins are estimated as having objects present within them, demonstrating the poor condition of range estimation without exploiting carefully crafted priors.

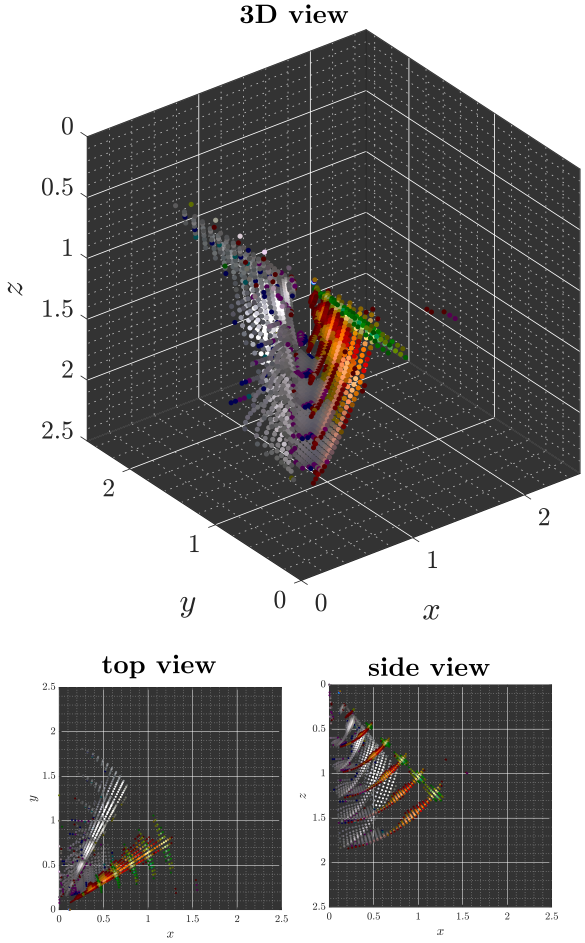

(a) Scene reconstruction.

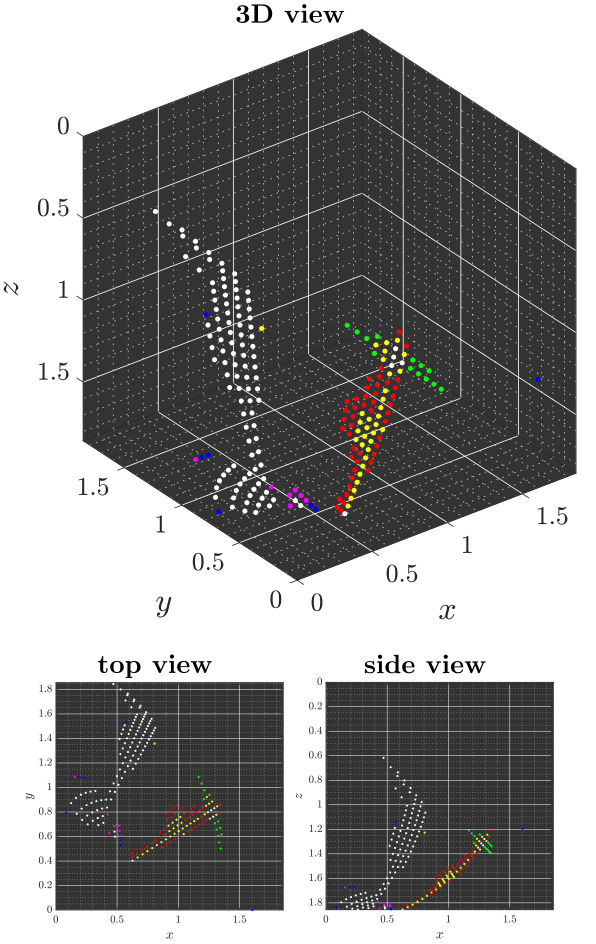

(b) Reconstruction averaged along the range axis.

Supplementary Figure 31: Joint non-sequential inversion of the fully linear forward model for USF scene. (a) The scene is reconstructed with significant blurring in range, as it is estimated to occupy many ranges bins (i.e., range bins 2 through 7, as shown in Supplementary Figure 32). (b) The reconstruction in (a) is averaged along the range bins, and displayed with an arbitrarily chosen range of $\rho = 2$ m.

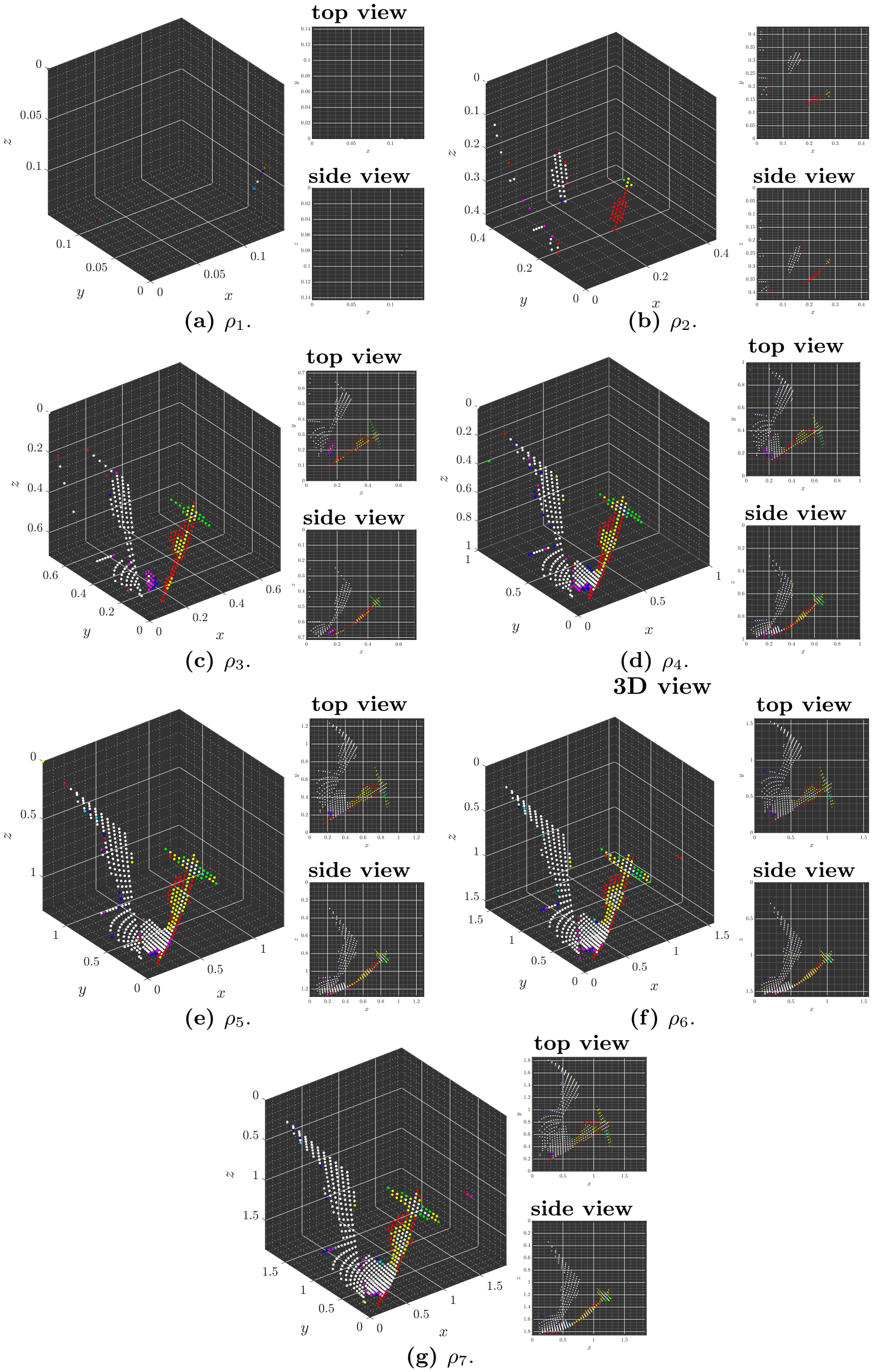

(a) ρ_1. (b) ρ_2. (c) ρ_3. (d) ρ_4. (e) ρ_5. (f) ρ_6. (g) ρ_7.

Supplementary Figure 32: Joint non-sequential inversion of the fully linear forward model for the two-mannequins scene. The radiosities estimated for range bins $\rho_1, \rho_2, \ldots, \rho_7$ are shown in panels (a) to (g), respectively. All range bins are estimated as having objects present within them, demonstrating the poor condition of range estimation without exploiting carefully crafted priors.

Supplementary Note 7

S7 Towards principled cluster refinements: Feasibility of splitting and merging clusters

A simple clustering of surface elements based on proximity might incorrectly reconstruct two objects that are at different ranges as a single cluster of surface elements, when the two objects overlap in azimuth and/or projected elevation angles. Depending on the true sizes of the objects, successively segmenting the objects into smaller sub-clusters can yield a reconstruction with reduced range estimation bias.

To demonstrate the feasibility of successive division of a large cluster into smaller sub-clusters, we consider two scenarios:

- **Single-object scenario (Supplementary Figure 33).** This scenario explores the reconstruction of a single large planar piece of card. The aim is to show that for an object that occupies a single range, segmenting it into smaller sub-clusters has little impact on the range estimate.

- **Two-object scenario (Supplementary Figure 34).** This scenario explores the reconstruction of two planar objects that are separated in range but appear as neighbours in azimuth and projected elevation angles. The aim is to show that subdividing the large cluster yields more accurate (low bias) range reconstructions.

The experimental scenarios used in this feasibility study are configured so that the scene in each of the two scenarios—one object in Supplementary Figure 33 and two objects in Supplementary Figure 34—cover the same angular extent, approximately. Thus, their angular reconstructions (obtained in Step 1) should almost be the same: A single large rectangular cluster of surface elements.

In Supplementary Figure 33, a turquoise-coloured rectangular card is placed at a range of 1.04 m. In Supplementary Figure 34, two turquoise-coloured rectangular cards with equal azimuthal and projected elevation angular extents are placed in the hidden scene such that they are contiguous along the azimuthal axis, but with different ranges from the origin (as shown in Supplementary Figure 34(a)). The nearest object was placed at 1.041 m, while the farthest was at 1.65 m from the origin. For the objects to have equal angular extents, the farther object is larger than the closer one.

For the two scenarios above, we utilize the following iterative algorithm to refine the large cluster returned upon completing STEP I of the proposed TERI reconstruction algorithm (see Section S4).

Preliminary Cluster Refinement Algorithm:

1. *Split clusters:* Divide each cluster computed in STEP I of the TERI reconstruction algorithm, along its center-of-mass in angles θ and ψ, to obtain four sub-clusters per initial cluster.

2. *Update ranges:* Initialize the ranges for all sub-clusters to be the ranges of their parent cluster, and recompute their range estimates by solving the NL range reconstruction problem using Algorithm 1.

3. *Merge clusters:* Recombine sub-clusters that belong to the same parent cluster, if their new range estimates are within 10 cm. (We chose 10 cm because it is roughly the estimated accuracy of the range estimates in our experiments.)

4. *Update ranges:* Recompute range estimates for new clusters by solving the NL range reconstruction problem using Algorithm 1. Initialize with the average range of each new combination of sub-clusters.

5. Repeat steps 3 and 4 until convergence (when the number of sub-clusters stops changing).

For the single-object case, whose final reconstruction (with the TV-based radiosity refinement) is shown in Supplementary Figure 33(e), it can be seen that the ranges of the sub-clusters are relatively

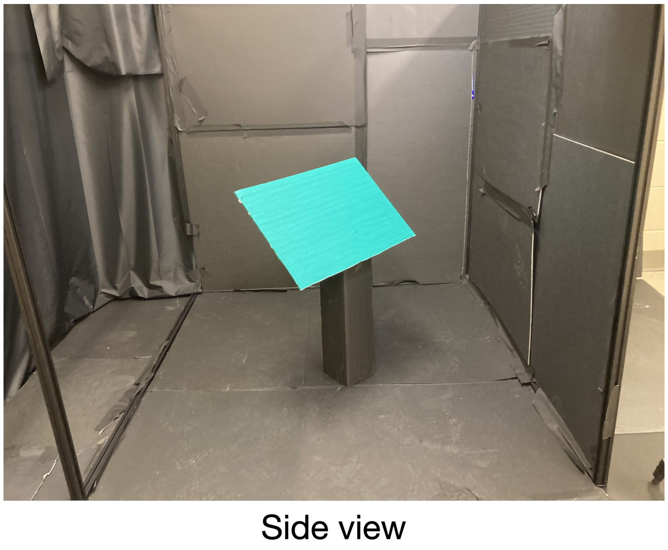

(a) Photograph of the hidden scene.

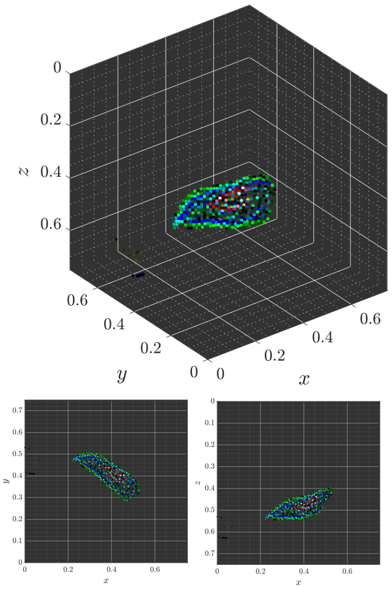

(b) Recon. after Step I of TERI.

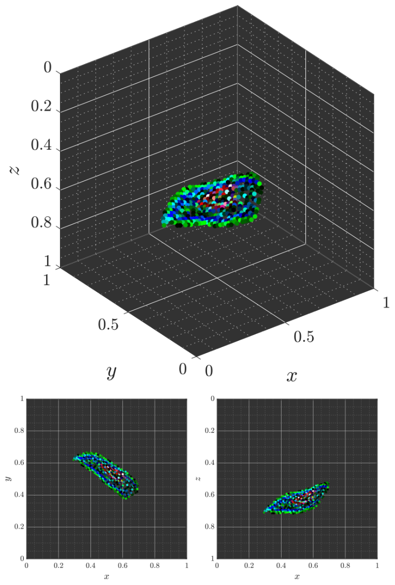

(c) Recon. after Step II of simplified TERI algorithm (estimated range $\widehat{\rho} = 1.00$ m).

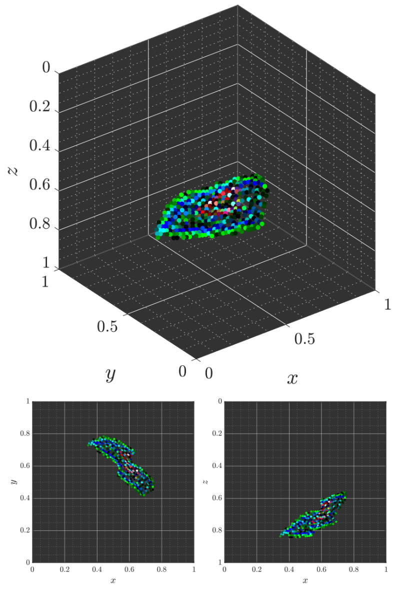

(d) Recon. after line 2 of the prelim. cluster refinement (sub-cluster ranges $1.064, 1.099, 1.150, 1.161$ m).

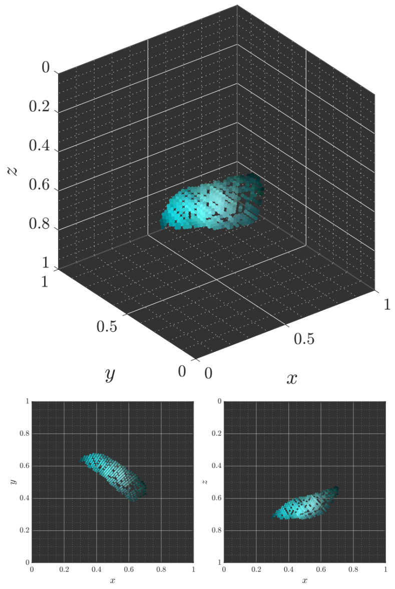

(e) Final recon. after convergence of the prelim. cluster refinement, and TV radiosity refinement.

Supplementary Figure 33: Scene reconstruction with cluster refinement algorithm for single-object scene. (a) The scene is a rectangular card whose measured range is 1.04 m. (b) The estimated shape of the hidden scene after Step I of TERI concludes. (c) The initial reconstruction of the hidden scene after Step II of the simplified (no TV) TERI algorithm; the estimated cluster range is 1.00 m. (d) Intermediate reconstruction after splitting the cluster and updating their range estimates (i.e., after running lines 1 and 2 of the *preliminary cluster refinement algorithm*); estimated sub-cluster ranges are $1.064, 1.099, 1.150, 1.161$ m. (e) Final reconstruction after convergence of the *preliminary cluster refinement algorithm*, followed by TV-regularized scene radiosity update.

close to each other, as well as the ground truth value when estimating their ranges independently. The algorithm correctly returns the original cluster instead of smaller sub-clusters.

The intermediate reconstructions for the two-object experiment are shown in Supplementary Figures 34(b) to 34(d). Although the two objects have different ranges, they are azimuthally contiguous and with equal solid angles; thus, Step 1 of TERI incorrectly fuses them into a single cluster. Step 2 of the TERI algorithm subsequently returns a highly biased range estimate (0.73 m) for the single large cluster (see Supplementary Figure 34(c)). As the preliminary cluster refinement scheme proceeds, sub-clusters are formed and correctly merged based on range proximity, terminating with two distinct sub-clusters. The final reconstruction, shown in Supplementary Figure 34(e), highlights the effectiveness of the preliminary cluster refinement scheme for the two-object experiment. The new ranges are highly accurate with reduced bias.

This preliminary algorithm suggests that there may be enough information in the measurements to split sufficiently large clusters into smaller ones with the aim of reducing bias in the range estimates. With the TV regularized estimate, Supplementary Figure 34(e), we notice there is a clear separation in the brightness of each sub-cluster. This is because the farthest object receives and thus reflects less light, than the nearer one. In addition to the size of a cluster, changes in brightness levels could also be exploited in deciding where and how to segment a larger cluster.

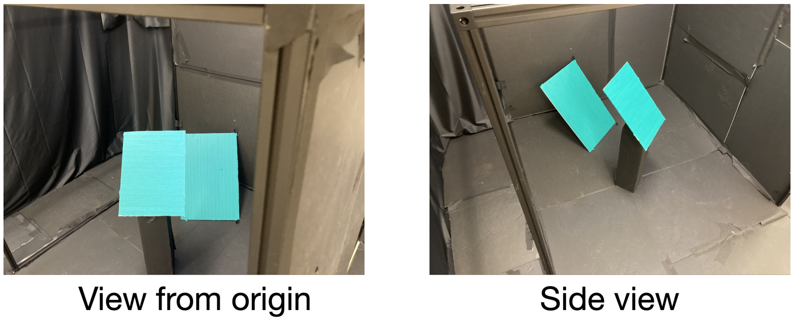

(a) Photographs showing different views of ground truth hidden scene.

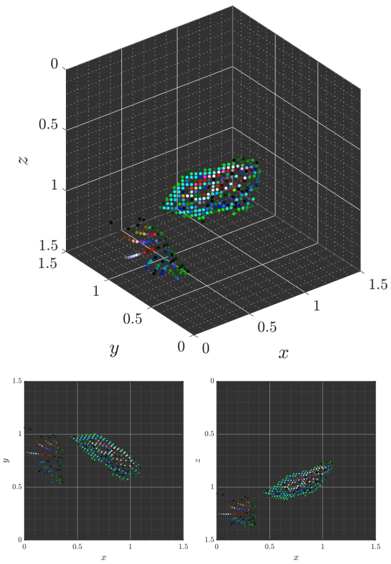

(b) Recon. after Step 1 of TERI.

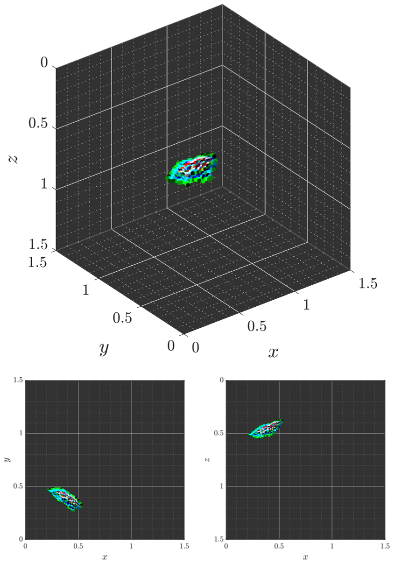

(c) Recon. after Step II of simplified TERI algorithm (estimated range $\widehat{\rho} = 0.73$ m).

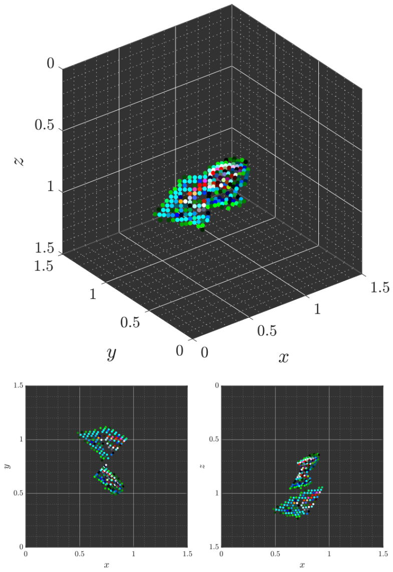

(d) Recon. after line 2 of the prelim. cluster refinement (est. sub-cluster ranges $1.24, 1.32, 1.67, 1.64$ m).

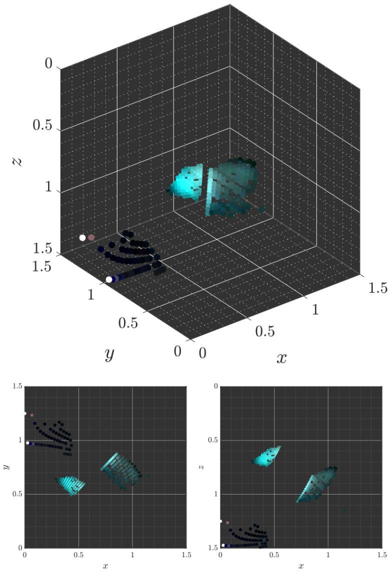

(e) Final cluster recon. after convergence and TV radiosity refinement (est. ranges 0.99 m and 1.48 m).

Supplementary Figure 34: Scene reconstruction with cluster refinement algorithm for a two-object scene. (a) The scene is two rectangular cards whose measured ranges are 1.04 m and 1.55 m. (b) The estimated shape of the hidden scene after Step I of TERI concludes. (c) The initial reconstruction of the hidden scene after Step II of the simplified (no TV) TERI algorithm; the estimated cluster range is 0.73 m. (d) Intermediate reconstruction after splitting the cluster and updating their range estimates (i.e., after running lines 1 and 2 of the *preliminary cluster refinement algorithm*); estimated sub-cluster ranges are $1.24, 1.32, 1.67, 1.64$ m. (e) Final reconstruction after convergence of the *preliminary cluster refinement algorithm*, followed by TV-regularized scene radiosity update.

Supplementary References

[1] Katherine L. Bouman, Vickie Ye, Adam B. Yedidia, Fredo Durand, Gregory W. Wornell, Antonio Torralba, and William T. Freeman. Turning corners into cameras: Principles and methods. In *Proc.*

IEEE Int. Conf. Comput. Vis., pages 2270–2278, 2017.

[2] William Krska, Sheila W. Seidel, Charles Saunders, Robinson Czajkowski, Christopher Yu, John Murray-Bruce, and Vivek Goyal. Double your corners, double your fun: The doorway camera. In *Proc. IEEE Int. Conf. Comput. Photogr.*, pages 1–12, 2022.

[3] Sheila W. Seidel, Yanting Ma, John Murray-Bruce, Charles Saunders, William T. Freeman, Christopher C. Yu, and Vivek K Goyal. Corner occluder computational periscopy: Estimating a hidden scene from a single photograph. In *Proc. IEEE Int. Conf. Comput. Photogr.*, pages 25–33, 2019.

[4] Sheila W. Seidel, John Murray-Bruce, Yanting Ma, Christopher Yu, William T. Freeman, and Vivek K Goyal. Two-dimensional non-line-of-sight scene estimation from a single edge occluder. *IEEE Trans. Comput. Imaging*, 7:58–72, 2021.

[5] Joshua Rapp, Charles Saunders, Julián Tachella, John Murray-Bruce, Yoann Altmann, Jean-Yves Tourneret, Stephen McLaughlin, Robin MA Dawson, Franco NC Wong, and Vivek K Goyal. Seeing around corners with edge-resolved transient imaging. *Nature Commun.*, 11(1):5929, 2020.

[6] Sheila Seidel, Hoover Rueda-Chacon, Iris Cusini, Federica Villa, Franco Zappa, Christopher Yu, and Vivek K Goyal. Non-line-of-sight snapshots and background mapping with an active corner camera. *Nature Commun.*, 14(1):3677, 2023.

[7] Ole E Barndorff-Nielsen and David Roxbee Cox. *Inference and asymptotics*, volume 13. Springer, 1994.

[8] G. B. M. Zerr. Biangular coordinates. *The American Mathematical Monthly*, 17(2):34–38, 1910.

[9] Charles Saunders, John Murray-Bruce, and Vivek K Goyal. Computational periscopy with an ordinary digital camera. *Nature*, 565(7740):472–475, January 2019.

[10] Stephen Boyd, Neal Parikh, Eric Chu, Borja Peleato, Jonathan Eckstein, et al. Distributed optimization and statistical learning via the alternating direction method of multipliers. *Foundations and Trends® in Machine learning*, 3(1):1–122, 2011.

[11] Amir Beck and Marc Teboulle. A fast iterative shrinkage-thresholding algorithm for linear inverse problems. *SIAM J. Imaging Sci.*, 2(1):183–202, 2009.
