# Supplementary material for: Two-edge-resolved three-dimensional non-line-of-sight imaging with an ordinary camera
Source: Nat Commun. 2024 Feb 7;15:1162. doi: 10.1038/s41467-024-45397-7 (PMC11258226; doi:10.1038/s41467-024-45397-7)
Supplement: Supplementary file 3 — Description of Additional Supplementary Files [file 41467_2024_45397_MOESM3_ESM.docx]

**Description of Additional Supplementary Files**

**Supplementary Movie 1:** **Overview of two-edge-resolved imaging method.** This movie provides a narrated description of the acquisition configuration, hidden scene representation, and the reconstruction algorithm, and shows revolving views of the 3D reconstructions presented in the main manuscript.

**Supplementary Movie 2:** **Visualisation of the projected-elevation spherical coordinate surface elements.** This movie provides an animated visualization of surface elements in the proposed projected-elevation spherical coordinate system.
