# Supplementary figures and images for: Two-edge-resolved three-dimensional non-line-of-sight imaging with an ordinary camera
Source: Nat Commun. 2024 Feb 7;15:1162. doi: 10.1038/s41467-024-45397-7 (PMC11258226; doi:10.1038/s41467-024-45397-7)

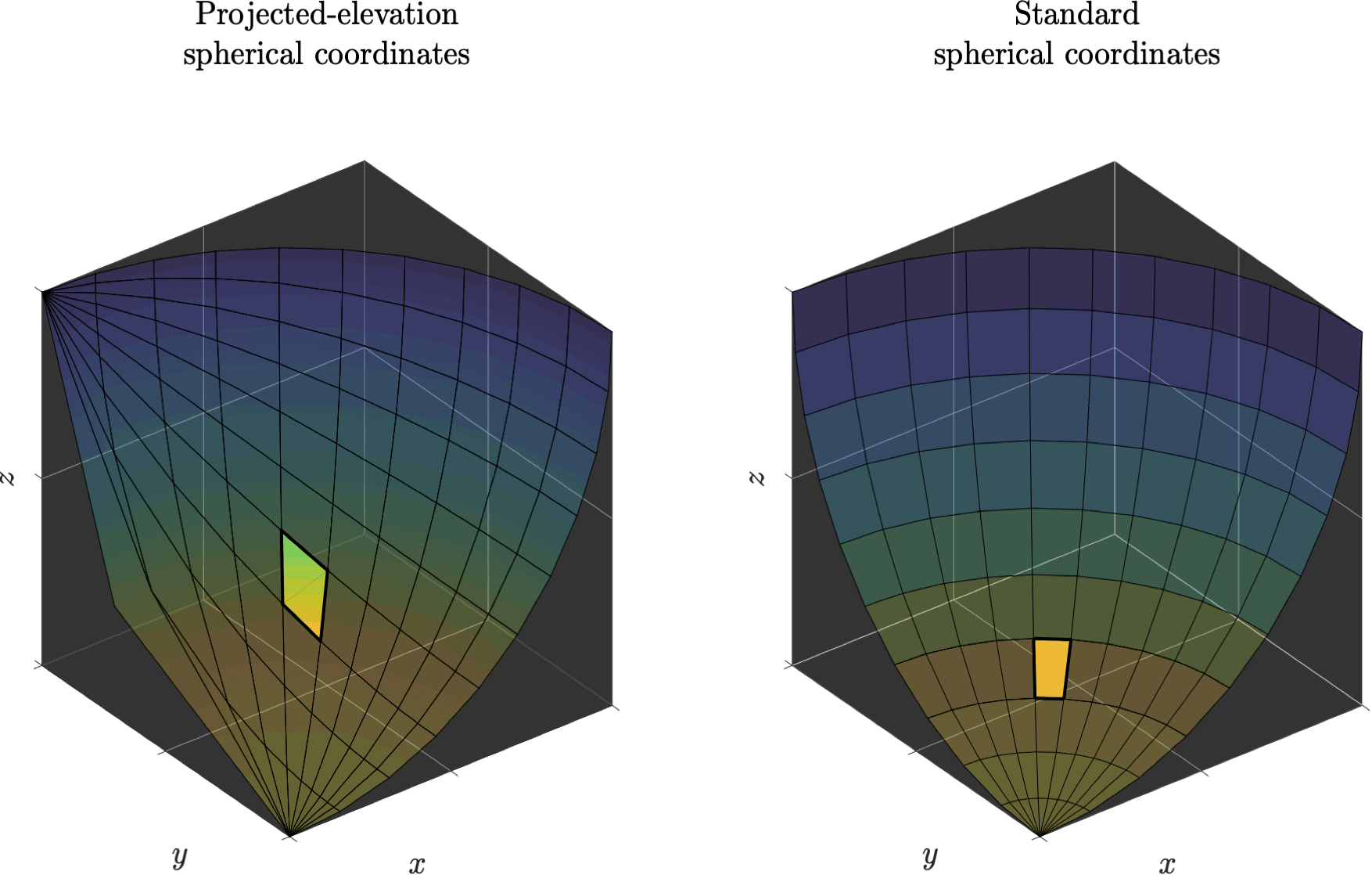

Supplement: Supplementary file 5 — Supplementary Movie 2 [file 41467_2024_45397_MOESM5_ESM.gif]
